# Overconfidence is universal? Elicitation of Genuine Overconfidence (EGO) procedure reveals systematic differences across domain, task knowledge, and incentives in four populations

**DOI:** 10.1371/journal.pone.0202288

**Published:** 2018-08-30

**Authors:** Michael Muthukrishna, Joseph Henrich, Wataru Toyokawa, Takeshi Hamamura, Tatsuya Kameda, Steven J. Heine

**Affiliations:** 1 Department of Psychological and Behavioural Science, London School of Economics and Political Science, London, United Kingdom; 2 Department of Human Evolutionary Biology, Harvard University, Cambridge, Massachusetts, United States of America; 3 Canadian Institute for Advanced Research, Toronto, Ontario, Canada; 4 School of Biology, University of St Andrews, St Andrews, United Kingdom; 5 School of Psychology and Speech Pathology, Curtin University, Perth, Australia; 6 Department of Social Psychology, The University of Tokyo, Tokyo, Japan; 7 Department of Psychology, University of British Columbia, Vancouver, Canada; University of Wuerzburg, GERMANY

## Abstract

Overconfidence is sometimes assumed to be a human universal, but there remains a dearth of data systematically measuring overconfidence across populations and contexts. Moreover, cross-cultural experiments often fail to distinguish between placement and precision and worse still, often compare population-mean placement estimates rather than individual performance subtracted from placement. Here we introduce a procedure for concurrently capturing both placement and precision at an individual level based on individual performance: The Elicitation of Genuine Overconfidence (EGO) procedure. We conducted experiments using the EGO procedure, manipulating domain, task knowledge, and incentives across four populations—Japanese, Hong Kong Chinese, Euro Canadians, and East Asian Canadians. We find that previous measures of population-level overconfidence may have been misleading; rather than universal, overconfidence is highly context dependent. Our results reveal cross-cultural differences in sensitivity to incentives and differences in overconfidence strategies, with underconfidence, accuracy, and overconfidence. Comparing sexes, we find inconsistent results for overplacement, but that males are consistently more confident in their placement. These findings have implications for our understanding of the adaptive value of overconfidence and its role in explaining population-level and individual-level differences in economic and psychological behavior.

## Introduction

Overconfidence has been described as “one of the most consistent, powerful and widespread (psychological biases)” [1], with “no problem… more prevalent and more potentially catastrophic” [2]. Overconfident CEOs make poorer investment and merger decisions [3, 4], overconfident traders increase trade volume and lead markets to underreact to relevant information and overreact to anecdotal information [5], overconfident leaders are more likely to go to war even when the odds are stacked against them [6], and overconfident people are more likely to start a business, even though most businesses fail [7]. On the other hand, overconfident people take on more ambitious projects, persist in the face of adversity [8], and have better mental and physical health [9, 10]. Regardless of whether overconfidence has a net benefit or cost, the common assumption underlying all these claims is that although there exist individual differences and perhaps differences by domain, overconfidence, at least on average, is universal.

Illustrating this assumption, Johnson and Fowler [1] published a model of the evolution of overconfidence. Despite the fact that two equilibria emerged under most conditions in the model—for either underconfidence or overconfidence, depending on the ratio of benefits to costs—Johnson and Fowler speculated that humans may have faced a sufficiently high benefit to cost ratio over the course of human history, such that overconfidence has become a genetic predisposition.

Researchers in the psychological and the economic sciences have been studying overconfidence somewhat independently, often not citing work in the others’ discipline. In psychology, research on the cognitive biases underlying overconfidence goes back to at least the early 1960s [11], continuing through the work of researchers such as Frank Yates and George Wright [12–15]. A separate but overlapping body of psychological research focused on the motivational aspects of overconfidence. This motivational bias, referred to as *self-enhancement*–the bias toward viewing the self positively–has its roots in early research on the self and self-esteem [10, 16, 17]. More recently, economists have been drawing on and extending the early psychological work on the cognitive biases underlying overconfidence, but have largely ignored this parallel research on self-enhancement and motivational biases underlying overconfidence.

Rather than universal, the broad body of research on this topic suggests that overconfidence is highly variable, varying by age [18], sex [18–23], population [12–15, 17, 24–26], domain content [20, 23, 27, 28], and domain context [21, 29], sometimes disappearing altogether or being replaced by underconfidence [24, 30, 31] and with interactions across several of these predictors.

For population differences, much research has suggested that East Asian populations are far less overconfident than Westerners, and sometimes even demonstrate striking underconfidence or self-criticism as opposed to self-enhancement [32, 33]. Moreover, these population differences have also been identified using measures that have employed hidden behavioral measures, or measure the overly positive assessments indirectly, indicating that the population difference is not merely the product of self-presentation motives [33, 34]. In sum, the universality of overconfidence is difficult to assess, given that its magnitude appears so differently across studies.

Part of the difficulty in interpreting these results is that although researchers regularly use the term “overconfidence”, they often mean very different things. Moore and Healy [35] provide a useful set of definitions for different overconfidence concepts:

1. *Overestimation* is the belief that you are better than you really are compared to an objective standard (e.g., believing you can consistently perform a flawless parallel park, when in reality you get it right 3 times out of 10).
2. *Overplacement* is the belief that you are better than more people than you really are (e.g., believing you are in the 90^th^ percentile of drivers, when in reality you are in the 10^th^ percentile).
3. Finally, *overprecision* is having more confidence in your beliefs than is justified (e.g., being 90% certain that you’re a better driver than average when you don’t have enough data to ascribe that level of certainty).

Each of these forms of overconfidence may be driven by both motivational factors (such as wanting to view yourself positively; e.g. [10, 36]) and cognitive factors (such as the availability bias or an inability to represent distributions; e.g. [37, 38]). The term *self-enhancement*, which generally refers to the motivation to view oneself positively rather than negatively, particularly compared to other people [17], may underlie overestimation and overplacement. The relationship between overprecision and self-enhancement is less clear. The self-enhancement literature has largely focused on how positively people compare themselves to others and not on the confidence that they have in the precision of their evaluations. In principle, viewing oneself as better than most other people *and* being confident in this belief may increase self-enhancement, but overplacement and overprecision are mutually separable concepts. That is, you may view yourself as better than other people and be confident in this belief, but equally you may view yourself as much worse than other people and be confident in this belief. In both cases, overprecision is high, but the latter case is unlikely to increase self-enhancement.

Although different definitions, and sometimes equivocation, may help explain some of the diverse results found in the literature, a related and equally challenging issue is that the same concepts may be operationalized in very different ways. Measuring overconfidence can be difficult and many researchers choose to use aggregate comparisons to judge overconfidence. For example, in the classic *Better than Average effect*, researchers claim high overconfidence when “93% of drivers claim to be above average”. But of course, many of those who claim to be better than average may actually be better than average (this may even be true of most of the population given a skewed distribution, see [39]), and conversely these results may hide some underconfidence, where those who are truly better than average claim less confidence than those who are worse than average [40]. Further, people don’t really seem to be able to conjure up what “average” means in the first place [37].

Despite these difficulties, the broader literature presents the intriguing possibility that overconfidence may vary across populations, perhaps due to the differential costs and benefits created by the specific physical and social environments [1]. Many factors can create psychological differences between populations [41–43] and these factors may moderate many of the predictors of overconfidence. For example, in competition, a fomenter of overconfidence, sex differences in choosing to compete were opposite between patrilineal and matrilineal social structures (women in matrilineal societies were more competitive than men, see [44]). If populations do systematically vary in overconfidence, this may help explain differences in innovation rates [45, 46]. From a different angle, even when overconfidence is costly for the individual, whose business is likely to fail, it may be beneficial for the society, since the businesses that do succeed give the society a competitive advantage against other societies, allowing overconfidence (or underconfidence) to evolve via cultural evolution driven by intergroup competition.

In the present set of studies, we developed a special case of the *Subjective Probability Interval Estimates* (SPIES) method [47, 48] and used it to test several theoretical and empirical claims and bring some order to the literature using specific and precise operationalizations of overconfidence. The SPIES method involves presenting participants with the range of possible outcomes separated into intervals. Participants then assign a probability estimate to each interval where estimates sum to 100%. The method allows the researcher to capture overprecision using some measure of the spread, such as a confidence interval. In the present study, we develop a special case of SPIES to capture both estimated placement and estimated precision with a concrete method of creating the probability distribution that can be used to incentivize for accuracy. This *Elicitation of Genuine Overconfidence* (EGO) method involves giving participants tokens or currency (e.g. ten $1 coins) which they can distribute in placement deciles (e.g. 0-9^th^ percentiles, 10-19^th^ percentile, 20-29^th^ percentiles, and so on.) By allowing participants to keep the currency in the correct decile, we incentivize them to give a genuine estimate of both their estimate placement and their precision in that placement without having to think about percentages or distributions. Overprecision is captured using a measure of spread, as in SPIES (e.g. standard deviation) and overplacement is captured by a measure of central tendency (e.g. mean) greater than actual placement based on performance. We ran large, cross-cultural studies in Japan, Hong Kong, and Canada, using the EGO method and also measured several variables which have been previously found to predict overconfidence. The EGO method allowed us to compare people’s self-assessments to their actual performance under conditions where they were or were not incentivized for accuracy—by using tokens or currency. The EGO method was used in both concrete (math test using questions from the Graduate Record Examinations; GRE) and ambiguous (empathy using the “Revised Reading the Mind in the Eyes” test which involved identifying the emotions expressed in images of only eyes from Western and East Asian individuals) tasks, and we measured judgments both before and after participants completed the tasks. We will distinguish *Overplacement* as traditionally measured—predicted placement above the population mean, from *True Overplacement*—predicted placement above actual placement. We will operationalize overprecision as the standard deviation in the token/coin distribution. It is worth noting that we have no way of measuring what accurate precision would be without going through the procedure multiple times–e.g. would a 90% confidence interval capture the performance mean 90% of the time? However, we will refer to a smaller standard deviation as greater overprecision. These concepts and their corresponding operationalizations are summarized in Table 1.

**Table 1:** Key overconfidence definitions for concepts and their corresponding operationalizations.

Concept	Operationalization
Overplacement: Belief that you are better than more people than you really are.	True Overplacement: Predicted placement minus actual placement. This is both an intuitive and correct operationalization of overplacement, but is rarely measured because it requires measuring actual performance. Overplacement: Predicted placement minus 50%. This is the common operationalization of overconfidence, but hides individual differences in ability and performance, which may vary independently.
Overprecision: More confidence in your beliefs than is justified by evidence.	The standard deviation of placement estimates, with smaller values indicating more overprecision.

The EGO method allowed us to test for population-level variation in these concepts, as well as study the effects of: (1) financial incentives (money vs. tokens), (2) type of task (math vs. empathy) and (3) updating based on perceived performance (before vs. after tasks) across all our populations. Our approach is also motivated by the Johnson-Fowler model. An individual’s decision to compete with others (e.g., by starting a business) is motivated by both their belief in placement and precision about this belief. For example, someone with high overplacement and high precision is more likely to start a business (an entrepreneur) than someone high in overplacement, but low in precision (a “wantrepreneur”), who may not risk as much. On the other hand, someone low in overplacement and high in precision would almost certainly not start a business (salaried worker), but someone low in both overplacement and precision may seek out more information to reduce their uncertainty before making the decision, or may not risk as much.

Our operationalizations are arguably closer to their underlying concepts than previous work. However, we have included the more commonly used operationalizations so as to compare our findings to this previous research. To the degree that our operationalizations concord with the operationalizations used in earlier work, we can use previous findings to guide our expectations. These hypotheses based on previous work are as follows:

1. Earlier work suggests that European Canadians/Americans would show higher overplacement compared to our Japanese sample and Hong Kong Chinese sample [24], but lower overprecision than the Hong Hong Chinese sample [13], which we expected to replicate here. Although there may be differences between Hong Kong Chinese, Taiwanese (as in Yates et al. 1998), and mainland Chinese samples, or indeed within China (e.g. [49]), Heine and Hamamura’s [24] meta-analysis reveals that these results have been found in Hong Kong Chinese samples, as used in our study.
2. We expected that participants would show less overplacement after taking the test [21] with the increase in knowledge of their performance.
3. We expected that participants would show more overplacement for the more uncertain and ambiguous task (empathy) compared to the more concrete task (math) [50].
4. We expected that incentives would increase the motivation for accuracy at the expense of motivations to feel positive about the self, motivations for self-presentation, or motivations for self-improvement. The few incentivized past results have suggested that Chinese were unaffected by incentives and Americans became more overconfident [14] or were unaffected [51] and that Japanese men and both Japanese and American women became overconfident, but American men were unaffected [29]. However, these were single studies with very different operationalizations, and in the case of Yamagishi et al. [29], population-level aggregates were used in one study, and in the other study the incentivized measurements were taken 8 months later, making it difficult to disentangle temporal changes from the effect of incentives and compare it to the present study. Past research with behavioral and indirect measures of overplacement largely replicates the population differences found in explicit self-report measures (for a review, see [34]).
5. Finally, based on past work, we expected that males [18–23] would show more overplacement, as would older people [18].
6. We had fewer expectations about overprecision, since it is less commonly measured, although recent work suggests it increases with age, consistent with increased self-knowledge over the lifespan [52, 53].

Our findings reveal that, rather than universal, levels of overplacement and overprecision vary considerably by task, population, feedback from taking the test, incentives, age, and sex, with interactions between these variables. In some cases, results differ depending on whether Overplacement or True Overplacement is measured, highlighting the importance of not using aggregate-level measures.

## Method

Ethical approval was granted specifically for this study by the University of British Columbia Behavioural Research Ethics Board under the title “Overconfidence Study” (ID: H12-02259). Informed consent was ensured by participants reading and signing a written consent form prior to beginning the study. A copy of this consent form has been included in S1 File.

Undergraduate students at the University of Hokkaido, Japan; the Chinese University of Hong Kong; and the University of British Columbia in Canada predicted their performance relative to other participants at their university. We compared these predictions to their actual performance (as well as to the 50th percentile, to compare with past research). Participants took two tests: a math test, which past research indicates higher self-knowledge and where performance should be less ambiguous due to familiarity and feedback through the educational system [54], and an empathy test, for which they should have less self-knowledge and where performance should be more ambiguous. Participants made predictions for their relative performance before and after taking the tests. In addition, participants were also randomly assigned to either be incentivized (using coins) or not incentivized (using tokens) for the accuracy of placement estimates. Participants estimated their relative placement using the Elicitation of Genuine Overconfidence (EGO) procedure. The EGO procedure involved distributing 10 coins or 10 tokens over 10 deciles; a more implicit method of eliciting placement than simply asking for a percentile judgment, which is less concrete and intuitive. In the incentivized condition, participants kept coins that were in the true performance decile. These methods allowed us to measure Overplacement (based on the coin/token central tendency or point estimates compared to the average performance), True Overplacement (based on the coin/token central tendency or point estimates compared to actual performance) and overprecision (based on the coin/token spread).

The EGO procedure can be performed with pen and paper, but we have made EGO software available on MM’s website: http://muth.io/ego-soft. The experimental protocol is available in S1 File. If you require assistance using or modifying the software, please contact MM.

### Procedure

All instructions were provided using a standardized script to ensure that all participants received the same information in the same way (the script is available in S1 File). We translated the Chinese and Japanese scripts from the English scripts using a back-translation method [55].

For each population, we began by collecting 20 pilot participants in the unincentivized (token) condition. These participants were not used in our analyses, but were used as a baseline to calculate payments for subsequent real participants. We split our Canadian sample into those of European and East Asian origin, however, these participants were told that they were competing against other participants in the experiment, which included Canadians of both ethnic backgrounds. Accordingly, we calculated performance relative to all Canadians rather than within their ethnic group, although results did not differ when performance was measured relative to co-ethnics. Our Canadian sample was prescreened for these two ethnic backgrounds to exclude other ethnicities.

All non-pilot participants were randomly assigned to either the incentivized (money) or unincentivized (token) condition. The order in which the two tests were administered (math first vs. empathy first) was also randomized, as were the questions within these computerized tests.

**Fig 1:**
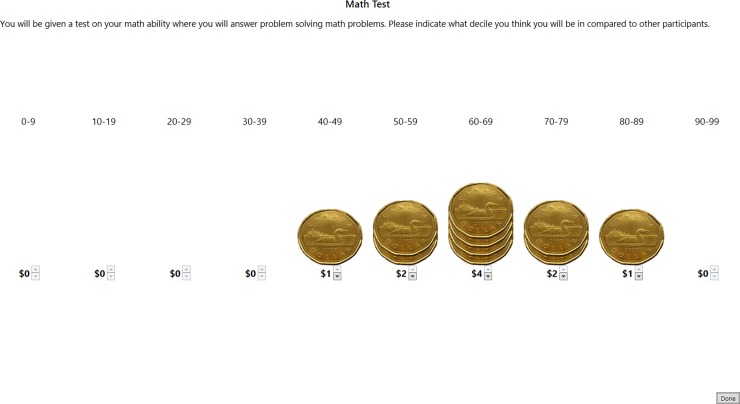
Screenshot from the EGO method money condition in Canada before the math test. Participants could choose how to distribute their coins across the 10 deciles. This particular participant indicates a belief that their performance will be around the 65^th^ percentile, with a tapering range that extends to just below average (40–49) to one of much above average (80–89), with a mean of 60–69.

Participants in all conditions were informed that they would get an entry into a lottery for every answer they got correct in both tests. The winner of the lottery was paid CAD100/HKD1000/JPY10,000. Participants in the money condition were further incentivized for accuracy in their relative performance on the tests. Participants in each country were given 10 coins of roughly comparable value (i.e. 10 CAD1/HKD10/JPY100 coins). To win this money, participants could place their 10 coins in any way they wanted across the 10 deciles (see Fig 1). They performed this task with 10 coins both before and after each test and were told that they would be paid the money in the decile that matched their relative performance for only 1 of these 4 occasions (before vs after for math vs empathy). By randomly paying for only 1 of these 4 occasions, participants were incentivized to maximize payoffs on all occasions, without “wealth effects”, where participants behaved differently later in the experiment based on their estimates of how much they had already won, reducing incentives. At the end of the study, participants drew a number from a box, which corresponded to 1 of these 4 times a placement estimate was made. So in the case of the example in Fig 1, this participant would win $4 if their performance was actually in the 60–69 decile, $2 if it was in either the 50–59 or 70–79 deciles, $1 if it was in either the 40–49 or 80–89 deciles, and zero if their performance was less than the 40–49 decile or in the 90–99 decile. This incentive for accuracy in placement was in addition to the incentive for performing well on the tests. For the purposes of paying participants, relative performance was calculated using the data from all prior participants, including the pilot group. For the purposes of analysis, relative performance was calculated on the complete sample of non-pilot data after exclusions. Participants were thus incentivized to perform as well as possible in the two tests in both the money and token conditions, and were incentivized to give an accurate estimate of their relative performance in the money condition. In the token condition, coins were replaced with 10 tokens and no mention was made about winning money in this way. The EGO method allowed us to measure both (1) how participants believed they compared to their peers (an index of overplacement) and (2) how confident they were in this belief (an index of overprecision), by looking at the mean and standard deviation of the decile distribution, respectively.

Since the decile measure was novel, participants were trained using a cardboard decile grid and 10 real coins or tokens. After training participants were asked questions, which they had to get correct to continue. These included: (1) who are you competing with? (2) What is a decile? And (3) How can you win money? If the participant got any of these questions wrong, the relevant part of the script was re-read and the questions were asked again. The full script is available in S1 File.

When participants began the experiment, the instructions were re-iterated and then a further training test was administered to check that participants knew how much they could win in the money condition in different situations. In both the explanation by the experimenter and this additional training, examples were balanced (i.e., both an example of a high mean and an example of a low mean; both an example of low uncertainty and an example of high uncertainty), to ensure that the instructions did not influence participant behavior in any particular direction. Participants then indicated their placement using the EGO method before taking the first test.

The math test consisted of 30 multiple choice word problems taken from the quantitative section of practice Graduate Record Examinations (GREs), presented in a random order. Participants were given 20 minutes to complete this task. The empathy test consisted of the 72 questions comprised of the 36 question “Revised Reading the Mind in the Eyes” test [56], which had European eyes, and the 36 question Asian (Japanese) eyes version of this test [57]. Questions were presented in random order. Thus, all participants judged the eyes of both their own-race and the other race (at least at the coarse level of European vs. Asian eyes). The empathy test was untimed.

After the first test and corresponding placement estimates, participants were given several measures. They were given two measures that have reliably distinguished East Asian and Western samples in past research in terms of their self-enhancement: the Rosenberg Self-Esteem scale [58], which assesses overall positivity of the self-concept, and the False Uniqueness Task [59], which assesses how people evaluate their placement compared to their same-sex peers from their university, in terms of 10 abstract traits. These two tasks will allow us to compare how the present samples compare with those used in previous studies. Participants also completed the Big 5 Personality Inventory [60, 61] and the Prestige and Dominance scale [62]. Participants then took the second test with corresponding placement measurements, after which they completed further measures: the Self-Construal scale [63], and several demographic questions. In the Canadian sample, made up of two subsamples, the demographic questions included measures of length of time in Canada and acculturation (Identity Fusion Scale [64]; Vancouver Index of Acculturation [65]).

Participants were then debriefed and those in the money condition were paid. The winners of the CAD100, HKD1000, and JPY10,000 were paid after data collection was completed.

### Participants

The sample consists of undergraduate students at the University of Hokkaido, the Chinese University of Hong Kong, and the University of British Columbia. The Canadian sample was further divided into those who were of European or East Asian ancestry. All data herein refers to non-pilot data (i.e. those collected after the first 20 from each university). Participants were excluded for one of three reasons: (1) Technical errors, when data wasn’t saved or the participant accidentally started the tests without receiving instructions; (2) Failed vigilance checks, when participants failed to correctly answer a vigilance check question such as “Please click ‘Not at all’”; and (3) Exploiting the system, defined as putting all their money in the lowest decile and then performing at levels significantly below chance. Incentivizing performance on the two tests (independent of accurate performance predictions) was expected to prevent participants from exploiting the game in this way and this was true for all but 4 participants. Table 2 reports the total data collected, all exclusions, and age and sex information. Canadian exclusions are reported together as technical issues prevented us from determining ethnicity in some cases.

**Table 2:** Demographic details for all non-pilot participants.

		European Canadian	East Asian Canadian	Hong Kong Chinese	Japanese
**Total**	**Collected**	145		128	100
**Excluded**	**Technical**	6		5	6
**Excluded**	**Vigilance**	10		13	10
**Excluded**	**Exploit**	0		1	3
**Total**	**Analyzed**	66	63	109	81
**Age**	**Mean**	20.52	20.43	20.55	19.10
	**SD**	2.69	4.13	1.83	0.93
**Sex**	**Female**	34	33	59	28
	**Male**	32	30	50	53

## Results

### Comparability of present samples to past samples of self-enhancement measures

First, we note how our samples compared with those used in past research on self-enhancement, to discern whether our samples are unusual on relevant variables. The Rosenberg Self-Esteem scale and the False Uniqueness Task are routinely used in the self-enhancement literature and so we included these to give us a basis to compare our samples (insofar as exposure to our measures don’t change behavior). A meta-analysis of past research has found that Western and East Asian samples differ on these two measures with effect sizes of *d* = .94 and *d* = 1.2 (whereas Westerners differ from East Asian *Americans* with effect sizes of *d* = .32 and *d* = .53), for the Rosenberg and False Uniqueness Tasks, respectively [24]. For comparison, we regress these same measures on the dummy codes of each sample with European Canadians set as the reference group. We report the beta coefficients in Table 3 below.

**Table 3:** Standardized differences between Western population (European Canadians in our experiment) and other populations.

Self-enhancement Measure	East Asian–Western (Meta-analysis)	East Asian Americans–Western (Meta-analysis)	East Asian Canadians–Euro- Canadians	Hong Kong Chinese–Euro- Canadians	Japanese–Euro-Canadians
**Rosenberg Self-Esteem**	-0.94	-.32	-0.19 [-0.50, 0.12], p = .231	-0.56 [-0.83, -0.27], p < .001	-1.21 [-1.51, -0.92], p < .001
**False Uniqueness Task**	-1.16	-.53	0.21 [-0.11, 0.52], p = .194	0.50 [0.23, 0.78], p < .001	-0.64 [-0.93, -0.34], p < .001

*Note*. Based on a meta-analysis and in our sample. Self-esteem results are largely in the same direction as the meta-analysis, although East Asian Canadians are not significantly different to European Canadians. False Uniqueness results are in the opposite direction to past results for East Asian Canadians and Hong Kong Chinese, significantly so in the latter, but comparable to past results for the Japanese.

Our East Asian Canadian population are more self-enhancing than is typically measured and are mostly indistinguishable from the European Canadians. The Hong Kong Chinese are somewhere in-between typical self-esteem measures for East Asian Americans and East Asians compared to Westerners, but in the same direction, but are higher on False Uniqueness, a reversal of past results. The Japanese have self-esteem and false uniqueness results in the same direction, and of roughly the same magnitude. These results make it difficult to compare our East Asian Canadian and Hong Kong Chinese sample to previous self-enhancement results, but our Japanese sample is quite similar to past samples, increasing our confidence in the generalizability of those findings. In the next section, we correlate all of our different measures of self-enhancement, overplacement, and overprecision.

### Correlation between self-enhancement, overplacement, and overprecision

Here we correlate self-esteem, false uniqueness, Overplacement, True Overplacement, and Overprecision. We use the mean values for each participant to avoid inflating power. These correlations are reported in Table 4 below and are reported separately for each sample in the supplementary (S1–S4 Tables).

**Table 4:** Correlation between overplacement, overprecision, and self-enhancement measures.

	1: Self-esteem	2: False Uniqueness	3: Overplacement	4: True Overplacement	5: Overprecision
**1**	1				
**2**	0.26***	1			
**3**	0.13*	0.31***	1		
**4**	-0.01	0.20***	0.33***	1	
**5**	0.03	0.00	-0.15**	0.00	1

*Note*. N = 319; p < .001

** p < .01

* p < .05. Lower values of overprecision suggest greater precision.

These results reveal small correlations between false uniqueness and self-esteem, between self-esteem and Overplacement, and between false uniqueness and Overplacement and to a slightly lesser extent, false uniqueness and True Overplacement. Overplacement and True Overplacement are moderately correlated with Overplacement showing a small negative correlation with overprecision (i.e. lower values are associated with higher precision). This indicates two important things. First, our measures of overplacement and overprecision reveal only a weak relationship with standard measures used for self-enhancement. Our measures of overprecision and overplacement are theoretically distinguishable constructs and show no correlation here. Though theoretically, overprecision and overplacement may be independent, more work is required to statistically demonstrate this (see [66]). In the next section we discuss our main approach to analyzing these data.

### Analysis of primary measures

Here we present our strategy for analyzing how our key predictors—task type (math vs. empathy), incentives (money vs. tokens), feedback (before vs. after), and population (European Canadian, East Asian Canadian, Hong Kong Chinese, and Japanese)—affect Overplacement, True Overplacement, and overprecision. We also calculated a “reward for accuracy”—which assesses how effective the combinations of True Overplacement and overprecision were in generating payoffs.

Predicted placement was defined as the mean of the distribution of coins or tokens in deciles. Overplacement, consistent with past operationalizations, is 50% subtracted from this mean placement estimate. In contrast, True Overplacement, is the actual individual relative performance placement, subtracted from this mean placement estimate. That is:

Overplacement=Mean[DecileDistribution]−0.5

TrueOverplacement=Mean[DecileDistribution]−ActualPlacement

By these measures 0 would indicate no bias, a negative value indicates an underplacement bias, and a positive value an overplacement bias. We operationalize overprecision as the standard deviation of the decile spread. Lower values of the decile spread indicate more overprecision. That is:

Overprecision=SD[DecileDistribution].

To analyze the effect of our manipulations, we regressed Overplacement, True Overplacement and overprecision on our key predictors–task type (math vs. empathy), incentives (money vs. tokens), and feedback (before vs. after) using a multilevel regression model, looking at each population separately. We used random intercepts to control for the common variance associated with each participant providing us with 4 data points (before and after the two tasks). Note that differences between treatments for a given outcome variable are not multiple tests; they come from the coefficients of a single regression model for each outcome variable:

$$Overplacement=\beta_{0}+\beta_{1}∙Math+\beta_{2}∙After+\beta_{3}∙Incentives+\epsilon$$

$$True\;Overplacement=\beta_{0}+\beta_{1}∙Math+\beta_{2}∙After+\beta_{3}∙Incentives+\epsilon$$

$$Overprecision=\beta_{0}+\beta_{1}∙Math+\beta_{2}∙After+\beta_{3}∙Incentives+\epsilon$$

Since our condition predictors (task, feedback, and incentives) and populations are factors, the intercepts of our regression are the confidence and uncertainty of the reference group (the predictor set to 0). For example, in our default coding, the reference group is unincentivized empathy test before feedback. In this condition, for example, *Overplacement* = *β*_0_ (all other coefficients are multiplied by 0. The regression coefficient of each predictor tells us how the predictor increases or decreases overplacement compared to this baseline condition. For example, *β*_1_ is the increase in overplacement among those in the math condition compared to the empathy condition before taking the test and without incentives. *β*_0_ + *β*_1_ is the Overplacement value for this math, before the test, unincentivized group. In this way, we can use the same three regression models and simply switch the reference group and look at the value and significance of the intercept *β*_0_ to know the Overplacement, True Overplacement, and Overprecision of that condition where 0 is accuracy in placement or maximum overprecision.

Note that this analytic strategy of changing the reference group and looking at the intercept to look for overplacement would not work if you added controls, since now the intercept would represent overplacement when these controls were also equal to 0. However, this is what we want in looking at the effect of each sex within each condition. As with other conditions, we can look at the intercept coefficient and significance when we change the reference sex. These are not running separate tests to compare conditions–each has one regression model. By using a single regression model for each outcome, we avoid inflating our Type I error.

We report these regression models in Table 5 (Overplacement), Table 6 (True Overplacement), Table 7 (overprecision), and Table 8 (Reward for Accuracy). Rather than report multiple versions of the same model with reference groups changed, we instead plot the raw means and raw confidence intervals and then discuss the results by gradually unfolding the complexity. The statistics reported are statistical tests from the regression models reports in Tables 5, 6, 7 and 8.

**Table 5:** Multilevel model regression of Overplacement on the binary variables for task type (math), updating (after) and incentives (incentive). The intercept here is meaningful and tells us the level of True Overplacement when all other variables are 0, i.e. True Overplacement in empathy, before taking the test, without incentives for accuracy. We control for common variance from repeated measures using random intercepts for participants.

	European Canadians	East Asian Canadians	Hong Kong Chinese	Japanese	All Populations
Intercept	11.14*** (6.01, 16.26)	10.33*** (4.20, 16.46)	9.00*** (5.17, 12.84)	2.24 (-2.47, 6.94)	11.14*** (5.50, 16.77)
Math	-9.36*** (-13.31, -5.41)	-4.04* (-7.47, -0.61)	-0.74 (-3.82, 2.33)	1.52 (-1.48, 4.51)	-9.36*** (-13.04, -5.67)
After	-6.78*** (-10.73, -2.83)	-8.07*** (-11.50, -4.64)	-4.28** (-7.36, -1.21)	-1.98 (-4.97, 1.02)	-6.78*** (-10.47, -3.10)
Incentive	1.86 (-3.66, 7.38)	2.92 (-5.23, 11.07)	-1.23 (-5.81, 3.35)	3.71 (-1.75, 9.16)	1.86 (-4.55, 8.27)
EA Can					-0.81 (-8.45, 6.84)
HK					-2.13 (-9.00, 4.74)
JP					-8.90* (-16.44, -1.36)
EA Can:Math					5.32* (0.04, 10.59)
HK:Math					8.61*** (3.94, 13.28)
JP:Math					10.87*** (5.91, 15.84)
EA Can:After					-1.29 (-6.56, 3.98)
HK:After					2.50 (-2.17, 7.17)
JP:After					4.80+ (-0.16, 9.77)
EA Can:Incent					1.06 (-8.01, 10.14)
HK:Incent					-3.09 (-11.15, 4.97)
JP Incent					1.85 (-6.77, 10.47)
*N*	264 (66 Clusters)	252 (63 Clusters)	436 (109 Clusters)	324 (81 Clusters)	1276 (319 clusters)
*R*^*2*^ *Fixed*	.095	.051	.014	.016	.042
*R*^*2*^ *Total*	.256	.560	.244	.366	.350

^+^ *p* < .10

* *p* < .05

** *p* < .01

*** *p* < .001

**Table 6:** Multilevel model regression of True Overplacement on the binary variables for task type (Math), updating (After) and incentives (Incentive). The intercept here is meaningful and tells us the level of True Overplacement when all other variables are 0, i.e. True Overplacement in empathy, before taking the test, without incentives for accuracy. We control for common variance from repeated measures using random intercepts for participants.

	European Canadians	East Asian Canadians	Hong Kong Chinese	Japanese	All Populations
Intercept	10.49* (1.40, 19.59)	11.09** (3.27, 18.92)	6.28* (0.71, 11.85)	-2.52 (-10.25, 5.20)	10.49* (1.98, 19.01)
Math	-7.69** (-13.46, -1.92)	-4.36+ (-9.47, 0.75)	-0.56 (-4.46, 3.34)	1.64 (-3.31, 6.60)	-7.69** (-12.98, -2.40)
After	-6.78* (-12.55, -1.01)	-8.07** (-13.18, -2.96)	-4.28* (-8.18, -0.39)	-1.98 (-6.93, 2.98)	-6.78* (-12.07, -1.49)
Incentive	3.17 (-7.28, 13.61)	-0.19 (-10.25, 9.87)	3.80 (-3.20, 10.81)	11.42* (2.48, 20.37)	3.17 (-6.66, 12.99)
EA Can					0.60 (-10.95, 12.15)
HK					-4.21 (-14.59, 6.17)
JP					-13.02* (-24.42, -1.61)
EA Can:Math					3.33 (-4.24, 10.91)
HK:Math					7.13* (0.42, 13.84)
JP:Math					9.33* (2.20, 16.46)
EA Can:After					-1.29 (-8.87, 6.28)
HK:After					2.50 (-4.21, 9.20)
JP:After					4.80 (-2.33, 11.94)
EA Can:Incent					-3.35 (-17.26, 10.56)
HK:Incent					0.64 (-11.71, 12.99)
JP Incent					8.26 (-4.95, 21.47)
*N*	264 (66 Clusters)	252 (63 Clusters)	436 (109 Clusters)	324 (81 Clusters)	1276 (319 clusters)
*R*^*2*^ *Fixed*	.032	.028	.012	.040	.028
*R*^*2*^ *Total*	.369	.434	.365	.375	.382

^+^ *p* < .10

* *p* < .05

** *p* < .01

*** *p* < .001

**Table 7:** Multilevel model regression of overprecision (standardized standard deviation) on the binary variables for task type (Math), updating (After) and incentives (Incentive). We control for common variance from repeated measures using random intercepts for participants.

	European Canadians	East Asian Canadians	Hong Kong Chinese	Japanese	All Populations
Intercept	-0.22 (-0.50, 0.07)	-0.33* (-0.64, -0.03)	0.28* (0.05, 0.52)	-0.06 (-0.34, 0.21)	-0.22 (-0.54, 0.11)
Math	-0.05 (-0.20, 0.10)	-0.17* (-0.30, -0.04)	-0.35*** (-0.46, -0.24)	-0.24*** (-0.38, -0.10)	-0.05 (-0.19, 0.09)
After	-0.02 (-0.17, 0.13)	-0.04 (-0.17, 0.09)	-0.12* (-0.23, -0.01)	-0.04 (-0.18, 0.10)	-0.02 (-0.16, 0.13)
Incentive	0.12 (-0.22, 0.46)	0.22 (-0.20, 0.65)	0.54*** (0.22, 0.86)	0.33+ (-0.004, 0.66)	0.12 (-0.27, 0.51)
EA Can					-0.12 (-0.55, 0.32)
HK					0.50* (0.11, 0.89)
JP					0.16 (-0.28, 0.59)
EA Can:Math					-0.12 (-0.33, 0.09)
HK:Math					-0.30** (-0.48, -0.12)
JP:Math					-0.19+ (-0.38, 0.01)
EA Can:After					-0.02 (-0.23, 0.19)
HK:After					-0.10 (-0.29, 0.08)
JP:After					-0.02 (-0.22, 0.17)
EA Can:Incent					0.10 (-0.46, 0.66)
HK:Incent					0.42+ (-0.08, 0.91)
JP Incent					0.21 (-0.32, 0.74)
*N*	264 (66 Clusters)	252 (63 Clusters)	436 (109 Clusters)	324 (81 Clusters)	1276 (319 clusters)
*R*^*2*^ *Fixed*	.001	.021	.099	.044	.111
*R*^*2*^ *Total*	.487	.706	.696	.540	.647

^+^ *p* < .10

* *p* < .05

** *p* < .01

*** *p* < .001

**Table 8:** Multilevel model regression of Reward for Accuracy on the binary variables for task type (Math), updating (After) and incentives (Incentive). We control for common variance from repeated measures using random intercepts for participants.

	European Canadians	East Asian Canadians	Hong Kong Chinese	Japanese	All Populations
Intercept	0.67* (0.09, 1.25)	1.04*** (0.44, 1.64)	1.10*** (0.73, 1.46)	0.89*** (0.44, 1.33)	0.67* (0.12, 1.22)
Math	1.32*** (0.86, 1.78)	0.94*** (0.45, 1.44)	0.38* (0.07, 0.69)	0.50** (0.15, 0.85)	1.32*** (0.89, 1.75)
After	0.15 (-0.31, 0.61)	0.40 (-0.09, 0.90)	0.14 (-0.17, 0.45)	0.29 (-0.06, 0.64)	0.15 (-0.28, 0.58)
Incentive	-0.19 (-0.81, 0.43)	-0.29 (-1.00, 0.42)	-0.07 (-0.49, 0.35)	-0.05 (-0.52, 0.42)	-0.19 (-0.79, 0.40)
EA Can					0.43 (-0.25, 1.11)
HK					0.37 (-0.39, 1.12)
JP					0.22 (-0.52, 0.96)
EA Can:Math					-0.94*** (-1.48, -0.40)
HK:Math					-0.37 (-0.99, 0.24)
JP:Math					-0.82** (-1.39, -0.24)
EA Can:After					-0.01 (-0.56, 0.53)
HK:After					0.25 (-0.36, 0.87)
JP:After					0.14 (-0.44, 0.71)
EA Can:Incent					0.12 (-0.63, 0.87)
HK:Incent					-0.10 (-0.94, 0.75)
JP Incent					0.14 (-0.66, 0.94)
*N*	264 (66 Clusters)	252 (63 Clusters)	436 (109 Clusters)	324 (81 Clusters)	1276 (319 clusters)
*R*^*2*^ *Fixed*	.032	.028	.012	.040	.028
*R*^*2*^ *Total*	.369	.434	.365	.375	.382

^+^ *p* < .10

* *p* < .05

** *p* < .01

*** *p* < .001

Tables 5, 6, 7 and 8 use unincentivized empathy before feedback as the baseline since this most closely resembles the conditions in which overplacement or self-enhancement has been measured in the past—an ambiguous task, without incentives for accuracy, and without immediate feedback. From this starting point, we will then gradually add layers of complexity as we examine the effect of feedback, task type, and incentives in four different populations. After going through these main results, we calculate “reward for accuracy”—how the strategies found in each population affected payoffs. Finally, we discuss age and sex effects by adding these to the regression models.

This strategy of changing the reference group and focusing on the intercept allows us to look for patterns of overplacement and overprecision within each population and see if these statistically differ from the 0 point of accuracy. We can employ the same strategy to test if populations differ from each other within each condition by adding dummy coded population variables. We report this regression with all populations included in the final column of Tables 5, 6, 7 and 8.

Note: Immediately after participants made their decile estimates, we asked them what percentile they thought they would score in, how certain they were that this was the percentile that they would score in, and, then for a comparable decile measure, how certain they were that they would score 5% on either side of this percentile. We used the percentile estimate (which was not incentivized) to calculate a True Percentile Overplacement by subtracting the participant’s percentile based on performance. The correlation between True Overplacement and True Percentile Overplacement was large and significant (range from .94 to .97 within each sample). The correlation between overprecision and the point estimate equivalent were in the right direction, but much smaller (range from -.03 to -.25; it was a significant correlation for all groups except the East Asian Canadians). This suggests that people may find it difficult to assign a probability to their overprecision. For the East Asian Canadians it was effectively uncorrelated. Since the point estimate and decile estimate of True Overplacement were so highly correlated, and because the percentile estimate was not incentivized and is thus harder to interpret, we focus our analyses on the richer and less explicit measure of both overplacement and overprecision given by the decile measures.

### Multiple regression analysis

We used a multilevel regression model to understand the effects of each of our key variables of interest, regressing Overplacement, True Overplacement, overprecision, and Reward for Accuracy on each variable for each population separately. Since we get 4 data points from every participant, we use random intercepts for participant to account for the common variance. The intercept of the regression reveals the level of True Overplacement when all other variables are 0, i.e., True Overplacement in the empathy test, before taking the test and without incentives. All other variables are compared to this base condition. To avoid difficult to interpret interactions between the samples and our key predictors, we instead report regressions for each sample separately, but also present the regression with all populations in the same model.

The regression on True Overplacement in Table 6 reveals that in this base condition (empathy, before the test, unincentized), all but the Japanese are significantly overconfident in placement, with the two Canadian samples the most overplaced. The Japanese, if anything, are accurate or slightly underplaced–their placement is statistically indistinguishable from zero (accurate). Moreover, the Japanese are significantly less overplaced than the Euro Canadians (-13.02%, *p* = .026) and East Asian Canadians (-13.62%, *p* = .015), and marginally significantly less overconfident than Hong Kong Chinese (-8.80%, *p* = .074). See Jupyter file in S1 File for more details on this analysis and all analyses to follow.

All groups become more accurate after taking the empathy test unincentivized and become similar in overplacement. Indeed, they all become statistically indistinguishable from zero (accurate) and similar in value (though the Japanese err on the side of underplacement).

The math test varies by population, with both Canadians becoming significantly and marginally less overplaced, respectively, and Hong Kong Chinese and Japanese staying largely the same. Incentives seem to *increase* overplacement in the Japanese, who become significantly more overplaced and reach levels of overplacement comparable to all other groups. True Overcplacement is maximized by being a Euro-Canadian under incentives before the empathy test, whereas it’s minimized by being Japanese, not incentivized after the empathy test.

These results are complicated and we have yet to discuss overprecision and reward for accuracy. Instead of describing all key results with words alone, it’s perhaps easier to show all results by unpacking them slowly by graphing the raw values. When discussing the key findings, we’ll refer back to the regressions above to discuss the size of the results and whether they’re significant.

### Overplacement and true overplacement

We begin by looking at how Overplacement and True Overplacement differ by task and incentives, before and after performing the task. We plot the raw means for each sample, for each cell of our design with 95% confidence intervals to allow for visual comparisons between means. Let’s start with our baseline condition—empathy with incentives for accurate predicted placement.

#### Unincentivized empathy

**Fig 2:**
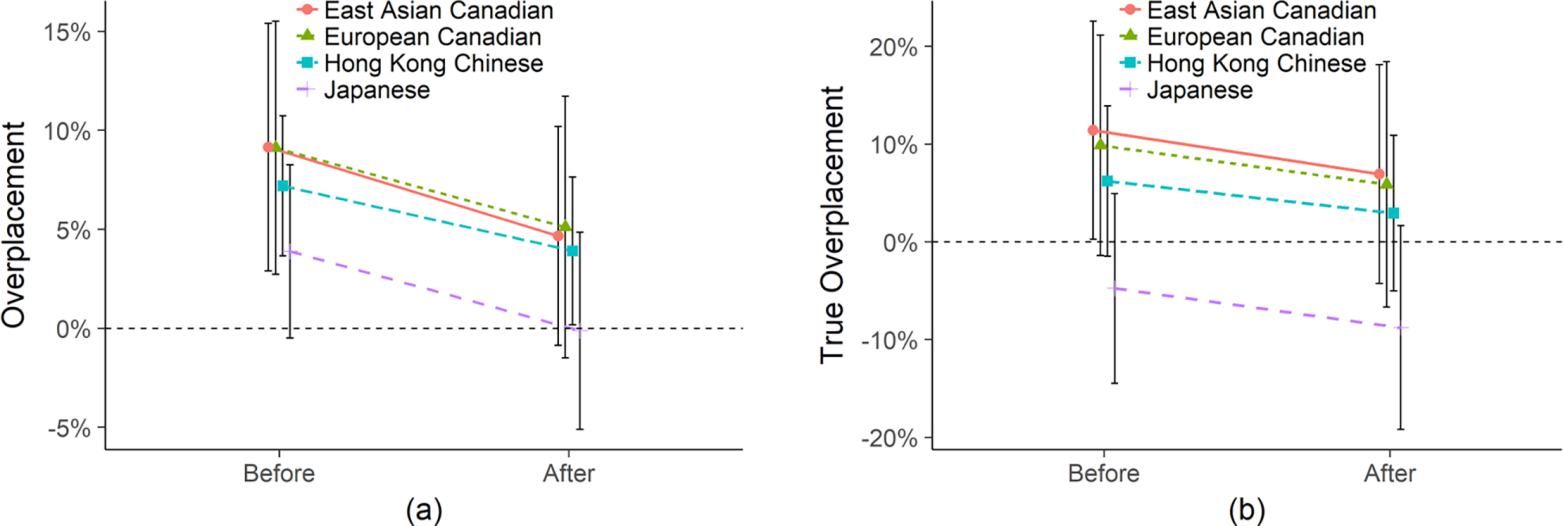
Empathy (a) Overplacement and (b) True Overplacement without incentives for placement accuracy. Error bars are 95% confidence intervals on the raw values. Note that the y-axis range is different to visually maximize the differences between lines.

In Fig 2 below, we plot Overplacement—predicted performance minus 50%—and True Overplacement—predicted performance minus true performance—for each sample before and after taking the empathy test with no incentives for accuracy in placement. By both measures of overplacement, we find that all samples update their predictions after taking the tests, with almost identical slopes towards less confidence. By the traditional overplacement measure (Fig 2A), all but the Japanese appear to be significantly overplaced. The Japanese also appear slightly overplaced, but are statistically indistinguishable from unbiased estimates (0%). After taking the test, all groups update towards less confidence, statistically indistinguishable from accurate, though the Euro Canadians (4.36%, *p* = .098) and the Hong Kong (4.72%, *p* = 0.017) participants are still significantly or marginally overplaced. However, these interpretations are misleading and reveal the danger in using population-level estimates. When we consider actual performance in the True Overplacement measure via the EGO method, the order of the samples is the same, but the measures are different. Here, as mentioned before, the Japanese are indistinguishable from accurate and erring on the side of slightly *underplaced* (though not significantly so; -4.50%, *p* = .256 after taking the empathy test) and are significantly less overplaced than the Euro Canadians (-13.02%, *p* = .026) and East Asian Canadians (-13.62%, *p* = .015), and marginally significantly less overplaced than Hong Kong Chinese (-8.80%, *p* = .074).

To summarize, Fig 2 shows that by the traditional measure, all samples appear to be overplaced before taking the test, except for the Japanese, who are statistically indistinguishable from accuracy. After taking the test, all samples become less overconfident, with all but the Hong Kong Chinese and perhaps the Euro Canadians, statistically indistinguishable from accurate. The True Overplacement measure tells a slightly different story–all samples are initially overplaced, except the the Japanese sample who are indistinguishable from accurate, erring on the side of underconfidence, and significantly less confident than all other samples. After taking the test, all populations are statistically indistinguishable from accurate, though the Japanese are even more underconfident (though still not statistically distinguishable from accurate).

We next look at how the results compare for the task that participants should have more self-knowledge about: math ability.

#### Does unincentivized math differ from unincentivized empathy?

**Fig 3:**
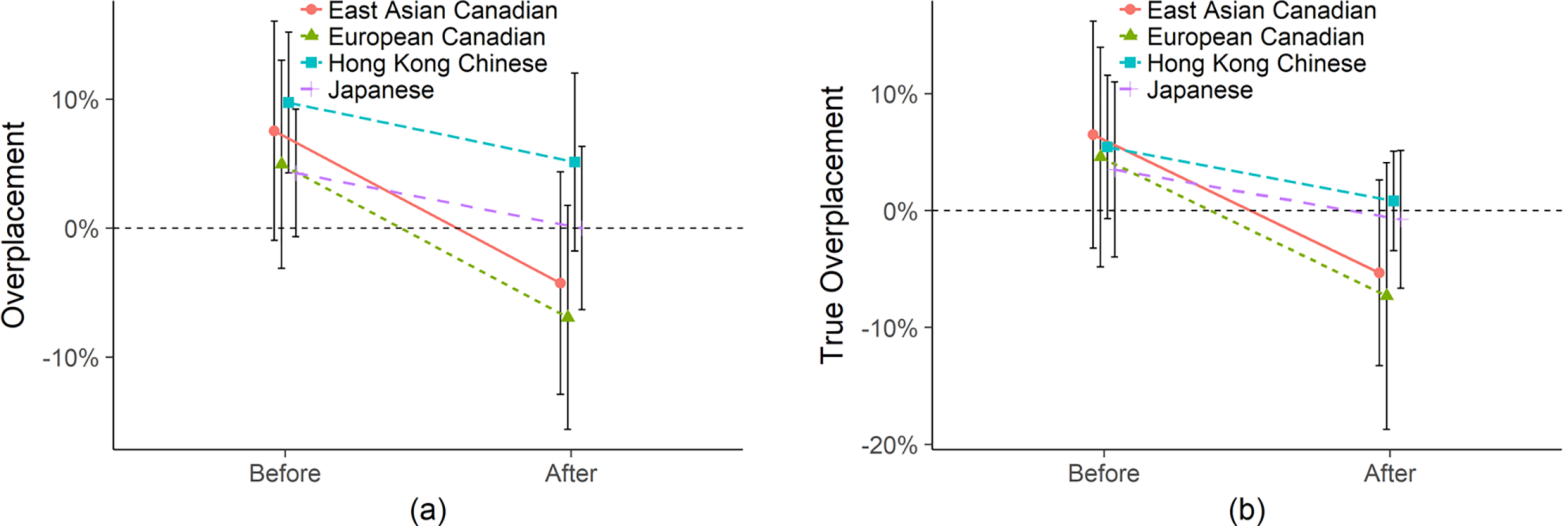
Math (a) Overplacement and (b) True Overplacement without incentives for placement accuracy. Error bars are 95% confidence intervals.

In Fig 3 below, we plot the same two graphs, without incentives for placement accuracy, but this time for the math test, an ability for which people should have more self-knowledge. Fig 3 shows several key differences in confidence on the math test compared to the empathy test. The most obvious difference is that by both Overplacement and True Overplacement, the two Canadian samples and the Hong Kong Chinese have much steeper updating, going from overplaced to accurate or even slightly underconfident and significantly less than their pre-test estimates. By the traditional Overplacement, Euro Canadians and East Asian Canadians appear to go from 1.78%, p = .497 to -5.00%, *p* = .058 (this difference is a significant decrease; -6.78%, *p* < .001) and 6.29%, *p* = .047 to 1.78%, *p* = .570 (this is a significant decrease as well; -4.04%, *p* = .022) respectively. Hong Kong Chinese go from 8.26, *p* < .001 to 3.98%, *p* = .044 (this is a significant decrease, -4.28%, p = .007). The Japanese appear to be more accurate and become slightly more accurate, though this decrease is not significant: 3.75%, *p* = .120 to 1.78%, *p* = .460 (the decrease is -1.98%, *p* = .198).

However, taking placement into consideration with True Overplacement, we see steeper updating among the two Canadian samples (though the difference between these slopes and the slopes of the other two populations is not statistically significant). All samples end up indistinguishable from accurate after taking the math test (as might be expected for a task with more self-knowledge). Euro Canadians go from 2.81%, *p* = .547 to -3.98%, *p* = .394 (decrease is -6.78%, *p* = .022);; East Asian Canadians go from 6.74%, *p* = .096 to -1.34%, *p* = .739 (decrease is -8.07%, *p* = .002), Hong Kong Chinese go from 5.72%, *p* = .046 to 1.44%, *p* = .614 (decrease is -4.28%, *p* = .032); and Japanese go from -0.88%, *p* = .824 to 2.86%, *p* = .470 (decrease is -1.98%, *p* = .435). Though the slope differences may suggest population-level differences in updating, these slopes are not significantly different from one another.

To summarize, Fig 3 shows that compared to the empathy results, before taking the tests, the four populations are much closer to each other in the size of overconfidence. Before and after taking the test, the Japanese are accurate by both measures, but all groups are indistinguishable from accurate after taking the test. We see a substantial drop in confidence by both measures among both Canadian groups to underconfidence and true underconfidence, though these are not statistically distinguishable from accurate.

Under no incentives for accurate placement estimates, the pattern of results in the math test compared to the empathy test are largely similar, except that the Euro Canadians are significantly more accurate about their math placement, *p* = .010 and more so than the Hong Kong Chinese and Japanese (*p* = 0.37 and *p* = .010). Without incentives, the Japanese are more accurate about both tests before and after. We next look at whether incentives for accuracy affect these results in both tests.

#### Do incentives affect overconfidence?

**Fig 4:**
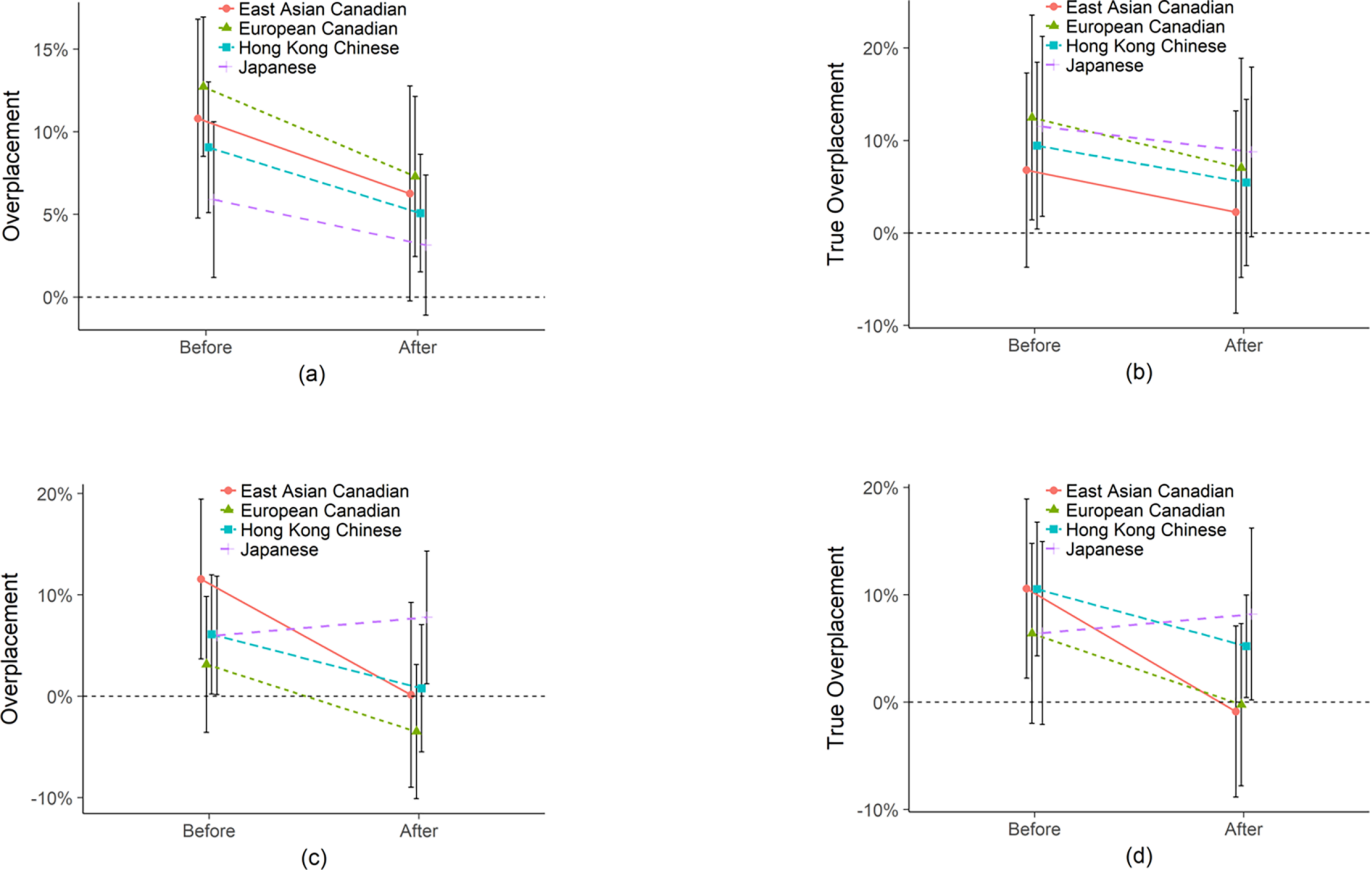
Empathy (a) Overplacement and (b) True Overplacement and Math (c) Overplacement and (d) True Overplacement. All with incentives for placement accuracy. Error bars are 95% confidence intervals. Note that the y-axis range for empathy is different so as to visually maximize differences between lines.

In Fig 4, we plot both the empathy and math test when participants were incentivized for accurate placement estimates. For a side-by-side comparison with the unincentivized condition, see S1 Fig. Under incentives, Fig 4 reveals quite different patterns. To begin with, all groups are significantly *more* overplaced by True Overplacement when incentivized before taking the empathy test, not less as some expect (Euro Canadians at 13.66%, *p* < .001; East Asian Canadians at 10.91%, *p* = .010; Hong Kong Chinese at 10.09%, *p* < .001; Japanese at 8.90%, *p* = .010). These results are exaggerated by the traditional Overplacement metric ranging from 5.94% to 13.25%, *p* < .01.

After taking the test, by True Overplacement, all but the Japanese significantly decrease their estimates (-6.78%, *p* = .022; -8.07%, *p* = .002; -4.28%, *p* = .032; and 1.98%, *p* = .435, respectively), but all remain overplaced, significantly so for the Hong Kong Chinese (5.80%, *p* = .050) and Japanese (6.93%, *p* = .045) and marginally significant for the European Canadians (6.88%, *p* = .083). The East Asian Canadians are indistinguishable from accurate (2.83%, *p* = .496).

Note the difference between Overplacement as traditionally measured and True Overplacement by considering performance: after taking the empathy test, the Japanese appear to be the least overconfident of the four groups by the Overplacement measure, but the most overconfident by the True Overplacement measure. This reversal highlights the need to consider performance and operationalize True Overplacement and not just population-level Overplacement. These results occur because the Japanese perform worse under incentives (see S2 Fig for performance differences).

In the math test, while the combination of incentives and feedback seems to reduce bias among the Canadians (by True Overplacement, Euro Canadians go from 5.97%, *p* = .132 to -0.81%, *p* = .837, decrease of -6.78%, *p* = .022, difference with empathy of 7.69%, *p* = .010; East Asian Canadians go from 6.55%, *p* = .118 to -1.52%, *p* = .714, decrease of -8.07%, *p* = .002, difference with empathy of 4.36%, *p* = .096), the Hong Kong and Japanese participants are fairly similar to the empathy test, remaining overplaced (Hong Kong Chinese go from 9.53%, *p* = .001 to 5.24%, *p* = .077, decrease of -4.28%, *p* = .032, difference with empathy of 0.56%, *p* = .779; Japanese go from 10.54%, *p* = .002 to 8.57%, *p* = .013, decrease of -1.98%, *p* = .435, difference with empathy of 1.64%, *p* = .517; see S5 and S6 Tables for supporting group comparisons). The combination of money and feedback in math actually makes the Japanese *more* overconfident, (11.42%, *p* = .014 higher than without incentives), perhaps because the Japanese found the math test easier than they expected—Japanese performance was better than every other group and significantly so under incentives (see S2 Fig).

To summarize, Fig 4 reveals that the pattern we see under incentives is quite different to the pattern with no incentives. Thus far, updating toward less overplacement after taking the test has seemed to be universal, but here we find that when it comes to math, the Japanese sample go from overconfident to even more overconfident. The importance of using True Overplacement is underscored by the empathy results where the Japanese go from the least overconfident group to the most overconfident group. This reversal occurs due to poorer performance under incentives. For the math test, both Overplacement and True Overplacement largely tell the same story, although Overplacement suggests that European Canadians are underconfident after taking the test, which is not true when you consider actual performance. European Canadians are nonetheless the least overconfident in math when incentivized for accuracy.

### Overprecision

**Fig 5:**
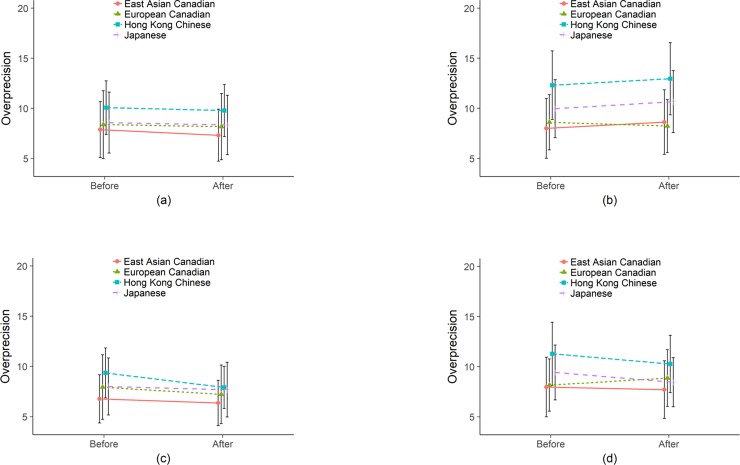
Uncertainty in Placement for Empathy (a) without incentives and (b) with incentives and for Math (c) without incentives and (d) with incentives. Overall, the Hong Kong Chinese and Japanese show more uncertainty than the Canadians (controlling for demographics) and we find more uncertainty in general when incentivized. Incentives increase uncertainty and uncertainty is greater for the more uncertain task, empathy. Error bars are 95% confidence intervals.

Overprecision, captured as the standardized standard deviation of the decile spread, aims to measure how much confidence participants had in their placement estimates. In Fig 5, we plot this standard deviation as we did for Overplacement and True Overplacement (a smaller value indicates more overprecision). Fig 5 suggests that East Asian Canadians are more overprecise than Euro Canadian participants who are more overprecise than the Japanese, who are in turn more overprecise than the Hong Kong Chinese. Compared to the Hong Kong Chinese, the Japanese are more overprecise by -0.35, *p* = .062, the Euro Canadians are more overprecise by -0.50, *p* = .013, and the East Asian Canadians are more overprecise by -0.62, *p* < .001. When money is on the line, all groups become less overprecise, though this difference is only significant for the Hong Kong Chinese (0.54, *p* = .001) and marginally significant for the Japanese (0.33, *p* = .056). All groups are also more overprecise for the concrete task (math) compared to the ambiguous task (empathy) and this difference is significant for the East Asian Canadians (-0.17, *p* = .012), Hong Kong Chinese (-0.35, *p* < .001), and Japanese (-0.24, *p* = .001), but not the Euro Canadians (-0.05, *p* = .519). Thus we again see a difference in reaction to incentives.

These results are supported by a regression analysis (Table 7). In the True Overplacement analysis, we were able to meaningfully interpret coefficients as percentages and use our intercept to indicate the presence of overconfidence (positive) compared to accuracy (zero) or underconfidence (negative). Here, our outcome variable is less meaningful.

These results indicate that populations are employing different strategies under different conditions along two dimensions—placement and precision. Both Canadian samples took more of a “go big or go home” strategy, putting more coins or tokens in fewer deciles, while the Hong Kong Chinese and Japanese took a more risk averse strategy, and the Hong Kong Chinese particularly so when real money was on the line. In terms of effect size, the influence of monetary incentives was comparable to being from Hong Kong. Being an East Asian Canadian (relative to Japanese) is comparable to being male. In the next section we measure how these strategies translate to payoffs in terms of how much money participants could have potentially taken home.

### Reward for accuracy

Here we consider how potential payoffs, that is how much money participants would have taken home if or when they were paid for that condition. In reality, participants were not paid for accuracy when using tokens (unincentivized) and were paid for one of the four stages of reporting placement (before and after each task) in the incentivized condition. We refer to this potential payoff as the “reward for accuracy”.

**Fig 6:**
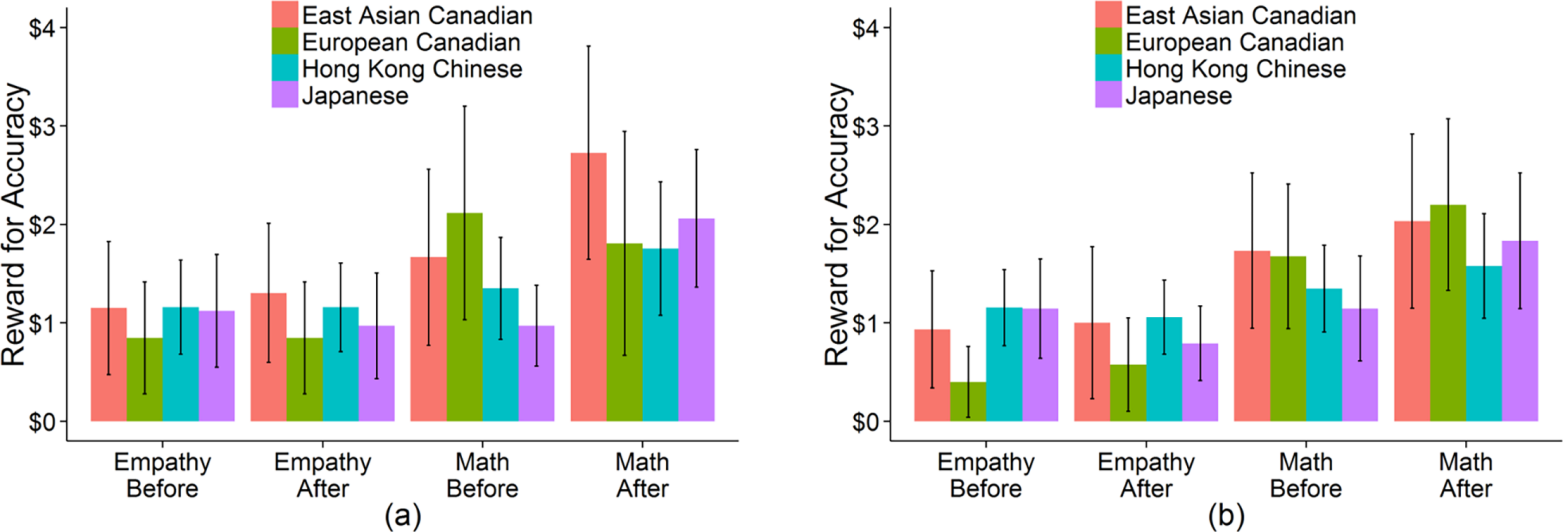
The mean number of (a) tokens or (b) money in the correct decile. Overall people did quite poorly getting close to chance ($1) in how much money they made. The mean was not substantially higher for real money compared to tokens and was in fact generally lower when using real money. The mean was higher in the task for which participants had more self-knowledge–math and in this task, taking the math test increased returns. The European Canadians mean was particularly low for empathy in with real money. Error bars are 95% confidence intervals.

Fig 6 and Table 8 reveals that despite the distinct strategies employed by different populations (e.g. “go big or go home” vs risk averse) little difference emerged in terms of payoffs. Furthermore, these payoffs were close to chance performance, indicating that participants had little in the way of accurate self-knowledge about these tasks. Perhaps surprisingly, using real money was not substantively different to using tokens, and if anything resulted in slightly lower payoffs. Unsurprisingly, participants had significantly higher payoffs in the task in which they had more knowledge—math and were generally able to update their estimates and increase their payoffs after taking the math test (though this increase was not significantly higher). Feedback from having taken the empathy test did very little to increase payoffs. Although these differences were not significant when money was on the line. The Canadian strategy of “go big or go home” paid off significantly better for the task for which they had more knowledge—math.

Overprecision differs by sex and age, with older individuals (-0.10, p = .028) and males (-.30, p < .001) showing more overprecision. Though sexes differed in precision, they did not differ in placement. Did this precision pay off? For Reward for Accuracy, males had slightly higher payoffs (27 cents, *p* = .047; see S5 and S6 Tables) controlling for other main effects, but also significantly higher variance (*ΔSD* = .38, *p* < .001; i.e., more winners and losers). In the next section, we discuss sex and age differences more broadly.

### Sex and age

Sex predicts both placement, precision, and performance. In general our results suggest that in contexts when males are more overplaced, the difference is much larger than in cases when females are more overplaced. However, females are often as overplaced or slightly more placed than males. So rather than males being consistently more overplaced than females, as is sometimes suggested by the literature, overplacement and performance differences between the sexes varies by incentives, task, and population. These results do not change the overall pattern of results so far reported.

Here we look at the difference between males and females in performance, Overplacement, True Overplacement, and overprecision within each sample for each test, with and without incentives, and for the estimates of performance, before and after each test. These patterns are complex, so we regress each outcome on sex and plot the coefficient of sex as a color ranging from red (females higher, shown by negative) to blue (males higher, shown by positive), where white indicates neither is higher. Significant differences are bolded and outlined in a darker black. Marginally significant are just bolded. We begin by looking at performance.

#### Performance

**Fig 7:**
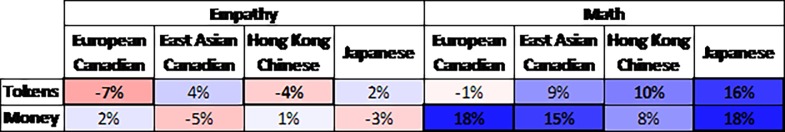
Performance as a percentage difference in raw score on test between males and females. Positive values (blue) indicate that males performed better. Negative values (red) indicate that females performed better. The color ranges from -20% to 20%. Statistically significant values are bolded and surrounded by a darker border. Marginally significant values are bold.

These results in Fig 7 indicate that men and women perform differently on the different tests between populations and under incentives. On the empathy test without incentives, European Canadian women and Hong Kong Chinese women did better, but under incentives there was only a small difference with men performing a bit better. The pattern was the opposite, but not significant among East Asian Canadians and Japanese.

Men in general performed better on the math test. European Canadian men performed significantly better under incentives, but there was no sex difference without incentives. Japanese men were consistently better at math, whereas East Asian Canadians were part way between these groups–men performing better overall, but more so under incentives.

In assessing overplacement, it is critical to take these performance differences into consideration, but first we’ll look at what you would find if you didn’t take these performance differences into account.

#### Overplacement

**Fig 8:**
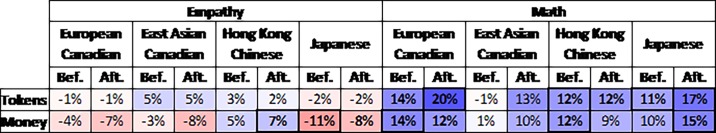
Traditional overplacement difference between males and females. Positive values and blue indicate that males had higher overplacement. Negative values and red indicate that females had higher overplacement. The color ranges from -30% to 30%. Statistically significant values are bolded and surrounded by a darker border. Marginally significant values are bold.

The results in Fig 8 highlight that men and women behave differently, but this is mostly based on test type. Without considering performance differences, men in all populations and conditions, except East Asian Canadians before taking the math test, show more overplacement (values above the mean) than women. For empathy, the predictions are more balanced, with Japanese women under incentives showing more overplacement. However, since we know that performance differs, these results are only meaningful in so far as they replicate past research.

At least for math, these results replicate past research, suggesting that men are more overplacement than women. But these results don’t take into consideration the performance difference previously discussed. Do the results change when we consider performance?

#### True overplacement

**Fig 9:**
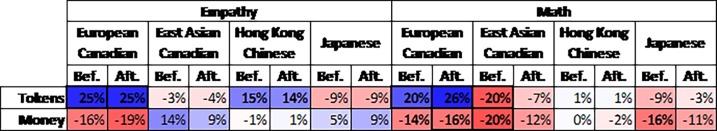
True Overplacement difference between males and females. Positive values (blue) indicate that males had higher true overplacement. Negative values (red) indicate that females had higher overplacement. The color ranges from -30% to 30%. Statistically significant values are bolded and surrounded by a darker border. Marginally significant values are bold.

When True Overplacement is measured, as shown in Fig 9, the strong sex differences disappear. In fact, only among European Canadians are they somewhat consistent, with unincentivized men showing more overplacement for both empathy and math. When incentivized, European Canadian women show more overplacement. East Asian Canadian women show more overplacement in math than East Asian Canadian men. This bias is driven by them not predicting their poorer performance. On empathy, both sexes are roughly the same, but East Asian Canadian men seem to be a bit more overconfident compared to women when incentivized. Results are mixed among the Hong Kong Chinese and Japanese with only marginally or not significant results.

Next, we consider sex differences in overprecision.

#### Overprecision

**Fig 10:**
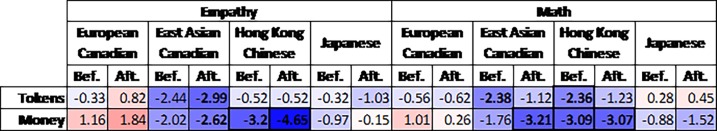
Overprecision difference between males and females as measured by differences in standardized standard deviations in the decile distribution. Positive values (red) indicate that males had higher uncertainty. Negative values (blue) indicate that females had higher uncertainty. The color ranges from -5 to 5. Statistically significant values are bolded and surrounded by a darker border. Marginally significant values are bold.

The results in Fig 10, coded so that more blue suggests males have more certainty suggest that the overall, East Asian Canadian and Hong Kong Chinese men show the most certainty. For the Japanese and European Canadians, the sexes act more similarly, with a slight trend towards higher female certainty among European Canadians for the empathy test.

The overprecision results suggest that men are generally more certain of their beliefs, but not universally so. And of course, we have no way of knowing if this certainty is warranted. Men do have slightly higher Reward for Accuracy payoffs, suggesting that this certainty may be warranted or at least pay off.

## Discussion

Here we introduce the EGO procedure for eliciting overconfidence through participant’s distributions rather than point estimates. This method allows us to concurrently capture both estimated placement and confidence or uncertainty in that placement (overprecision). It also lends itself to incentivizing the honesty of these distributions. Using the EGO, we show that overconfidence in placement and precision is inconsistent, sometimes weak, and cross-culturally variable, rather than “consistent, powerful, and widespread” [1]. These results challenge the idea that we can make broad and general claims about overconfidence. They make clear that overconfidence is highly dependent on incentives, context, available information, sex, and population. Nonetheless, the data offers some critical lessons and key findings:

1. It is crucial to distinguish between placement and precision in overconfidence. For example, though men and women can both be overconfident by placement estimates (overplacement), men do appear to generally be more certain about those estimates (overprecision). Similarly, though all groups appear to be overconfident by placement (at least when incentivized and prior to feedback), Hong Kong Chinese (and perhaps Japanese) show less certainty. The EGO method allows the simultaneous and implicit measurement of both placement and precision, and could easily be adapted to measure overestimation rather than overplacement. Indeed, EGO is a special case of the SPIES method, which has been successfully used in the context of overestimation [47, 48]. Overestimation is usually measured by individual estimates compared to actual performance (rather than population means), but is also often a point estimate that does not capture confidence or uncertainty in this estimate.
2. It is crucial to consider individual performance measures when comparing overplacement between groups. For example, Canadians and Hong Kong Chinese appear to be overconfident and Japanese accurate on the empathy task without incentives. However, when we consider individual performance, all populations are actually accurate and the Japanese tend towards underplacement (though not significant in our sample) and are significantly less confident than other populations. Under incentives, the Japanese seem like the least confident group when you compare estimates to mean performance, but are the most confident group when you compare estimates to individual performance! This discrepancy occurs because performance also varies between conditions.
3. Overplacement is prevalent, but not universal. In fact, by the True Overplacement measure, of our 32 cells (Task x Incentives x Feedback x Population), 22% were *underconfident*.
4. Feedback through taking the test generally results in more accurate estimates. The size of the update is similar between populations, with two exceptions in the math test. Under no incentives, both Canadian samples went from overconfident to accurate tending toward underconfident after taking the test, perhaps because they found the test particularly difficult–they performed more poorly than the East Asian populations. Conversely, under incentives, the Japanese actually *increased* in overplacement, perhaps because they found the test particularly easy—Japanese performed significantly better than all other populations.
5. Consistent with the idea that more information leads to more accurate estimates, individuals are more accurate in the task for which they should have more self-knowledge: math. We found that participants were more accurate in placement for math than empathy and more certain about this belief.
6. The effect of incentives is culturally specific. For True Overplacement, all but the Japanese were largely unaffected by incentives and in all cases, rather than making people more accurate, incentives resulted in higher True Overplacement.
7. The effect of incentives varies between placement and precision. When incentivized, most groups increased placement estimates, but *decreased* certainty about those estimates.
8. Populations differed in placement and precision, but payoffs from these distinct strategies was similar. Canadians used more of a “go big or go home” strategy, compared to the risk averse strategy employed by the Japanese and Chinese, particularly when money was involved (this finding appears to contradict the “cushion hypothesis,” which claims that East Asians are financially risk-seeking because they perceive a greater support network to rely on if they fail [67]). Payoffs (or potential payoffs) were largely the same between real money and tokens, and were remarkably small—rarely deviating from chance—indicating overall poor self-knowledge. However, payoffs did vary by task–participants generally made more money for math–and more so after taking the math test. Taking the empathy test had no effect on payoffs. The different population strategies made almost no difference to payoffs, although when real money was involved, the “go big or go home” strategy was marginally better for math, the higher self-knowledge task, and the more risk averse strategy, marginally better for empathy, the lower self-knowledge task. Of course, although they are roughly the same on-average, the “go big or go home” strategy will generate more variation in winning across those populations.

Our findings on culture, age, and sex are consistent with some and contrast to other prior research. The overprecision increase with age is consistent with past work and the idea that we improve self-knowledge as we mature [52, 53]. That the East Asian samples showed overplacement in the face of incentives similar to that of the European Canadian samples may seem at odds with past research finding pronounced population differences in self-enhancement using hidden behavioral and indirect measures (although we remind readers that the self-esteem and false uniqueness measures indicated that the East Asian-Canadian and Hong Kong Chinese samples were unusually self-enhancing; for reviews see [34, 36]). One reason for the different pattern of results may be that the measures used in this study tapped into somewhat distinct processes compared with those measures used in previous studies; this notion is supported by the modest correlations between the different measures of overplacement and self-enhancement presented in Table 4. An alternative account is that perhaps these conflicting findings indicate that East Asians adopt underconfident assessments of themselves as a strategy to motivate themselves for self-improvement, even if they are able to recognize, when incentivized to scrutinize their performance more closely, that they are being overly self-critical when doing so. People can have different motivations for assessing themselves, either to feel good about themselves, to attend to areas in need of improvement, or to accurately assess their standing (cf., [68]). That the Japanese and Hong Kong Chinese samples had overall greater uncertainty in placement suggests that they have weaker commitments to any single view of self. This may indicate that their various self-views are more in conflict with each other, and more dependent on circumstances, than they are for Westerners (see [68, 69]).

It is commonly claimed that men are more overconfident than women (although this is not reliably found in self-enhancement studies). We argue that this conclusion may be a result of measuring overplacement without considering performance differences. In the stereotypically male domain of math, males appear overconfident, but only when compared to the mean (males make higher placement estimates). When True Overplacement is calculated by subtracting actual performance, male overplacement evaporates, because males actually perform better on-average in stereotypically male domains. Instead, both men and women are overconfident in different contexts. However, men are more *certain* than women. It is difficult to say whether this is “overprecision”. One indication is how this certainty translates to payoffs. Men have slightly higher payoffs than women suggesting that the certainty may not be “over” what is adaptive, but males also have greater variance in payoffs in every population, suggesting that although this certainty pays off for males overall, the spread of winners and losers is larger than for females.

There are two key directions for future work. First, our motivation for this study was our interest in differences in overplacement and overprecision and how they may motivate decisions to become entrepreneurs. As discussed earlier, entrepreneurs are likely to be high in overplacement and overprecision, whereas high overplacement, but not overprecision maybe the classic “wantrepreneur”, who aspires to become an entrepreneur, but never makes the leap. To this end, we paid participants only for the coins placed in the correct decile—similar to a positive skew on the success of a new business. In this situation, expected value would be maximized by placing all coins in the highest probability decile. An alternative incentive structure would be to use a proper scoring rule (such as a Brier, logarithmic, or spherical score; for discussion see [70]). Using a proper scoring rule would incentivize a more honest distribution and account for the possibility that any increases in overprecision we see are a result of realizing what would maximize expected payoffs, regardless of overprecision.

The second direction is using different tasks to determine whether better self-knowledge is what is driving the results we see between tasks. We cannot exclude the possibility that other differences between the tasks drove the differences between tasks we see in our results. For example, we used the standard way in which these tests are administered, which meant that the math test had a time limit and fewer questions than the empathy test. This and other features may have led participants to believe that one test was easier than the other or that they would somehow perform better. Of course all participants experienced the same conditions for each test, we may be inadvertently tapping into some other aspect of overconfidence psychology beyond task knowledge. Running this protocol with different tasks would help answer this question.

We present EGO as a procedure and provide data from four populations, two distinct tasks, before and after feedback, and experimental manipulation of incentives. From these data, we argue that assumptions of universal overconfidence, even on average, do not stand up to the incredible variation in both placement and precision by domain, knowledge of the task, incentives, population, age, and sex. Instead, overconfidence appears to be a highly-context dependent strategy, suggesting caution against broad generalizations about overconfidence.

## Supporting information

S1 File Supplementary files including analytic script, copy of consent form, experimental protocol and additional analyses. Contains Analytic Script for Overconfidence.html; Analytic Script for Overconfidence.ipynb; Overconfidence ConsentForm.pdf; Overconfidence_Supplementary.pdf.
(ZIP)

S1 Table Correlation between overconfidence and self-enhancement measures for Euro Canadians. (PDF)

S2 Table Correlation between overconfidence and self-enhancement measures for East Asian Canadians. (PDF)

S3 Table Correlation between overconfidence and self-enhancement measures for Hong Kong Chinese. (PDF)

S4 Table Correlation between overconfidence and self-enhancement measures for Japanese. (PDF)

S5 Table Multilevel regression models without interactions. Multilevel regression models on the binary variables for task type (Math), updating (After) and incentives (Incentive), with population, age, and sex. We control for common variance from repeated measures using random intercepts for participants.
(PDF)

S6 Table Multilevel regression models with interactions. Multilevel regression models on the binary variables for task type (Math), updating (After) and incentives (Incentive), with population, age, and sex. This table includes interactions. We control for common variance from repeated measures using random intercepts for participants.
(PDF)

S1 Fig Side by side comparisons of True Overconfidence for empathy and math with and without incentives. Error bars are 95% confidence intervals. Note that the y-axis range is different so as to better visualize the differences between lines.
(TIF)

S2 Fig Actual raw performance (i.e. not relative to rest of population) for each population. For the Empathy under (a) no incentives and (b) incentives and the Math test under (c) no incentives and (d) incentives. Error bars are 95% confidence intervals.
(TIF)

## References

[1] ohnson DD, Fowler JH. The evolution of overconfidence. Nature. 2011;477(7364):317–20. 10.1038/nature10384 21921915

[2] Plous S. The psychology of judgment and decision making: Mcgraw-Hill Book Company; 1993.

[3] Malmendier U, Tate G. CEO overconfidence and corporate investment. The journal of finance. 2005;60(6):2661–700.

[4] Malmendier U, Tate G. Who makes acquisitions? CEO overconfidence and the market's reaction. Journal of Financial Economics. 2008;89(1):20–43.

[5] Odean T. Volume, volatility, price, and profit when all traders are above average. The Journal of Finance. 1998;53(6):1887–934.

[6] Johnson DD. Overconfidence and war: Harvard University Press; 2009.

[7] Camerer C, Lovallo D. Overconfidence and excess entry: An experimental approach. American economic review. 1999:306–18.

[8] Bénabou R, Tirole J. Self-confidence and personal motivation. The Quarterly Journal of Economics. 2002;117(3):871–915.

[9] Taylor SE, Kemeny ME, Reed GM, Bower JE, Gruenewald TL. Psychological resources, positive illusions, and health. American psychologist. 2000;55(1):99–109. 11392870 10.1037//0003-066x.55.1.99

[10] Taylor SE, Brown JD. Illusion and well-being: a social psychological perspective on mental health. Psychological bulletin. 1988;103(2):193–210. 3283814

[11] Adams JK, Adams PA. Realism of confidence judgments. Psychological Review. 1961;68(1):33–45. 13681400 10.1037/h0040274

[12] Wright GN, Phillips LD, Whalley PC, Choo GT, Ng K-O, Tan I, et al Cultural differences in probabilistic thinking. Journal of Cross-Cultural Psychology. 1978;9(3):285–99.

[13] Yates JF, Lee JW, Shinotsuka H, Patalano AL, Sieck W. Cross-cultural variations in probability judgment accuracy: Beyond general knowledge overconfidence? Organizational Behavior and Human Decision Processes. 1998;74:89–117. 9705815 10.1006/obhd.1998.2771

[14] Yates JF, Lee J-W, Bush JG. General knowledge overconfidence: cross-national variations, response style, and “reality”. Organizational behavior and human decision processes. 1997;70(2):87–94. 10.1006/obhd.1998.2771 9705815

[15] Yates JF, Zhu Y, Ronis DL, Wang D-F, Shinotsuka H, Toda M. Probability judgment accuracy: China, Japan, and the United States. Organizational Behavior and Human Decision Processes. 1989;43(2):145–71.

[16] Greenwald AG. The totalitarian ego: Fabrication and revision of personal history. American psychologist. 1980;35(7):603–18.

[17] Heine SJ, Lehman DR, Markus HR, Kitayama S. Is there a universal need for positive self-regard? Psychological review. 1999;106(4):766–94. 10560328 10.1037/0033-295X.106.4.766

[18] Ortoleva P, Snowberg E. Overconfidence in Political Behavior. American Economic Review. 2015;105(2):504–35.

[19] Barber BM, Odean T. Boys will be boys: Gender, overconfidence, and common stock investment. The Quarterly Journal of Economics. 2001;116(1):261–92.

[20] Beyer S, Bowden EM. Gender Differences in Self-Perceptions: Convergent Evidence from Three Measures of Accuracy and Bias. Personality and Social Psychology Bulletin. 1997;23(2):157–72.

[21] Lenney E. Women's self-confidence in achievement settings. Psychological bulletin. 1977;84(1):1–13.

[22] Chuang W-I, Lee B-S, Wang K-L. US and Domestic Market Gains and Asian Investors’ Overconfident Trading Behavior. Financial Management. 2013;43(1):113–48.

[23] Lundeberg MA, Fox PW, Punćcohaŕ J. Highly confident but wrong: Gender differences and similarities in confidence judgments. Journal of educational psychology. 1994;86(1):114–21.

[24] Heine SJ, Hamamura T. In search of East Asian self-enhancement. Personality and Social Psychology Review. 2007;11(1):4–27. 10.1177/1088868306294587 18453453

[25] Svenson O. Are we all less risky and more skillful than our fellow drivers? Acta Psychologica. 1981;47(2):143–8.

[26] Whitcomb KM, Önkal D, Curley SP, George Benson P. Probability judgment accuracy for general knowledge. Cross‐national differences and assessment methods. Journal of Behavioral Decision Making. 1995;8(1):51–67.

[27] Dunning D. Trait importance and modifiability as factors influencing self-assessment and self-enhancement motives. Personality and Social Psychology Bulletin. 1995;21(12):1297–306.

[28] Lichtenstein S, Fischhoff B. Do those who know more also know more about how much they know? Organizational Behavior and Human Performance. 1977;20(2):159–83.

[29] Yamagishi T, Hashimoto H, Cook KS, Kiyonari T, Shinada M, Mifune N, et al Modesty in self‐presentation: A comparison between the USA and Japan. Asian Journal of Social Psychology. 2012;15(1):60–8.

[30] Gigerenzer G, Hoffrage U, Kleinbolting H. Probabilistic mental models: a Brunswikian theory of confidence. Psychol Rev. 1991;98(4):506–28. . 1961771 10.1037/0033-295x.98.4.506

[31] Heine SJ. Where is the evidence for pancultural self-enhancement? A reply to Sedikides, Gaertner, and Toguchi (2003). Journal of Personality and Social Psychology. 2005;89(4):531–8. 10.1037/0022-3514.89.4.531 16287416

[32] Kitayama S, Markus HR, Matsumoto H, Norasakkunkit V. Individual and collective processes in the construction of the self: self-enhancement in the United States and self-criticism in Japan. Journal of personality and social psychology. 1997;72(6):1245–67. 9177018 10.1037//0022-3514.72.6.1245

[33] Heine SJ, Takata T, Lehman DR. Beyond self-presentation: Evidence for self-criticism among Japanese. Personality and Social Psychology Bulletin. 2000;26(1):71–8.

[34] Falk CF, Heine SJ. What is implicit self-esteem, and does it vary across populations? Personality and Social Psychology review. 2015;19(2):177–98. 10.1177/1088868314544693 25063044

[35] Moore DA, Healy PJ. The trouble with overconfidence. Psychological review. 2008;115(2):502–17. 10.1037/0033-295X.115.2.502 18426301

[36] Hamamura T, Heine SJ, Takemoto TR. Why the better-than-average effect is a worse-than-average measure of self-enhancement: An investigation of conflicting findings from studies of East Asian self-evaluations. Motivation and Emotion. 2007;31(4):247–59.

[37] Klar Y, Giladi EE. No one in my group can be below the group's average: a robust positivity bias in favor of anonymous peers. Journal of personality and social psychology. 1997;73(5):885–901. 9364753 10.1037//0022-3514.73.5.885

[38] Miller DT, Ross M. Self-serving biases in the attribution of causality: Fact or fiction? Psychological bulletin. 1975;82(2):213–25.

[39] Benoît JP, Dubra J. Apparent overconfidence. Econometrica. 2011;79(5):1591–625.

[40] Kruger J, Dunning D. Unskilled and unaware of it: how difficulties in recognizing one's own incompetence lead to inflated self-assessments. Journal of personality and social psychology. 1999;77(6):1121–34. 10626367 10.1037//0022-3514.77.6.1121

[41] Chudek M, Muthukrishna M, Henrich J. Cultural Evolution In: Buss DM, editor. The Handbook of Evolutionary Psychology. 2. 2nd ed. Hoboken, NJ: John Wiley and Sons; 2015.

[42] Henrich J, Heine SJ, Norenzayan A. The weirdest people in the world? Behavioral and Brain Sciences. 2010;33(2–3):61–83. 10.1017/S0140525X0999152X 20550733

[43] Henrich J, Heine SJ, Norenzayan A. Most people are not WEIRD. Nature. 2010;466(7302):29 10.1038/466029a 20595995

[44] Gneezy U, Leonard KL, List JA. Gender differences in competition: Evidence from a matrilineal and a patriarchal society. Econometrica. 2009;77(5):1637–64.

[45] Shane S, Venkataraman S, MacMillan I. Cultural differences in innovation championing strategies. Journal of Management. 1995;21(5):931–52.

[46] Muthukrishna M, Henrich J. Innovation in the collective brain. Philosophical Transactions of the Royal Society of London B: Biological Sciences. 2016;371(1690). 10.1098/rstb.2015.0192 26926282 PMC4780534

[47] Haran U, Moore DA, Morewedge CK. A simple remedy for overprecision in judgment. Judgment and Decision Making. 2010;5(7):467–76. 28405257 PMC5386407

[48] Moore DA, Carter AB, Yang HH. Wide of the mark: Evidence on the underlying causes of overprecision in judgment. Organizational Behavior and Human Decision Processes. 2015;131:110–20.

[49] Talhelm T, Zhang X, Oishi S, Shimin C, Duan D, Lan X, et al Large-scale psychological differences within China explained by rice versus wheat agriculture. Science. 2014;344(6184):603–8. 10.1126/science.1246850 24812395

[50] Dunning D, Meyerowitz JA, Holzberg AD. Ambiguity and self-evaluation: The role of idiosyncratic trait definitions in self-serving assessments of ability. Journal of personality and social psychology. 1989;57(6):1082–90.

[51] Williams EF, Gilovich T. Do people really believe they are above average? Journal of Experimental Social Psychology. 2008;44(4):1121–8.

[52] Moore DA, Dev AS, Goncharova E. Overconfidence Across Cultures. psyarxiv preprint. 2018 doi: 10.31234/osf.io/rhnxc

[53] Prims JP, Moore DA. Overconfidence over the lifespan. Judgm Decis Mak. 2017;12(1):29–41. ; PubMed Central PMCID: PMCPMC5978695. 29861807 PMC5978695

[54] Freund PA, Kasten N. How smart do you think you are? A meta-analysis on the validity of self-estimates of cognitive ability. Psychological bulletin. 2012;138(2):296–321. 10.1037/a0026556 22181852

[55] Brislin RW. Back-translation for cross-cultural research. Journal of cross-cultural psychology. 1970;1(3):185–216.

[56] Baron‐Cohen S, Wheelwright S, Hill J, Raste Y, Plumb I. The “Reading the Mind in the Eyes” test revised version: A study with normal adults, and adults with Asperger syndrome or high‐functioning autism. Journal of child psychology and psychiatry. 2001;42(2):241–51. 11280420

[57] Adams RB Jr, Rule NO, Franklin RG Jr, Wang E, Stevenson MT, Yoshikawa S, et al Cross-cultural reading the mind in the eyes: An fMRI investigation. Journal of Cognitive Neuroscience. 2010;22(1):97–108. 10.1162/jocn.2009.21187 19199419

[58] Rosenberg M. Society and the adolescent self-image. 1965.

[59] Campbell JD. Similarity and uniqueness: The effects of attribute type, relevance, and individual differences in self-esteem and depression. Journal of personality and social psychology. 1986;50(2):281–94. 3701578 10.1037//0022-3514.50.2.281

[60] John OP, Naumann LP, Soto CJ. Paradigm shift to the integrative big five trait taxonomy. In: John OP, Robins RW, Pervin LA, editors. Handbook of personality: Theory and research. 32008. p. 114–58.

[61] John OP, Donahue EM, Kentle RL. The big five inventory—versions 4a and 54. Berkeley: University of California, Berkeley, Institute of Personality and Social Research 1991.

[62] Cheng JT, Tracy JL, Henrich J. Pride, personality, and the evolutionary foundations of human social status. Evolution and Human Behavior. 2010;31(5):334–47.

[63] Singelis TM. The measurement of independent and interdependent self-construals. Personality and Social Psychology Bulletin. 1994;20(5):580–91.

[64] Aron A, Aron EN, Smollan D. Inclusion of Other in the Self Scale and the structure of interpersonal closeness. Journal of personality and social psychology. 1992;63(4):596–612.

[65] Ryder AG, Alden LE, Paulhus DL. Is acculturation unidimensional or bidimensional? A head-to-head comparison in the prediction of personality, self-identity, and adjustment. Journal of personality and social psychology. 2000;79(1):49–65. 10909877 10.1037//0022-3514.79.1.49

[66] Steinborn MB, Langner R, Flehmig HC, Huestegge L. Methodology of performance scoring in the d2 sustained-attention test: Cumulative-reliability functions and practical guidelines. Psychol Assess. 2018;30(3):339–57. 10.1037/pas0000482 . 28406669

[67] Hsee CK, Weber EU. Cross-national differences in risk preference and lay predictions. Journal of Behavioral Decision Making. 1999;12(2):165–79.

[68] Sedikides C, Strube MJ. Self-evaluation: To thine own self be good, to thine own self be sure, to thine own self be true, and to thine own self be better In: Zanna MP, editor. Advances in experimental social psychology. 29 New York: Academic Press; 1997 p. 209–69.

[69] Kim Y-H, Cohen D, Au W-T. The jury and abjury of my peers: The self in face and dignity cultures. Journal of Personality and Social Psychology. 2010;98(6):904 10.1037/a0017936 20515246

[70] Gneiting T, Raftery AE. Strictly proper scoring rules, prediction, and estimation. Journal of the American Statistical Association. 2007;102(477):359–78.

