# Supplementary material for: Overconfidence is universal? Elicitation of Genuine Overconfidence (EGO) procedure reveals systematic differences across domain, task knowledge, and incentives in four populations
Source: PLoS One. 2018 Aug 30;13(8):e0202288. doi: 10.1371/journal.pone.0202288 (PMC6116975; doi:10.1371/journal.pone.0202288)
Supplement: S1 File — Contains Analytic Script for Overconfidence.html; Analytic Script for Overconfidence.ipynb; Overconfidence ConsentForm.pdf; Overconfidence_Supplementary.pdf. (ZIP) [file pone.0202288.s001.zip › Analytic Script for Overconfidence.html]

Analytic Script for Overconfidence


In [95]:

```
library(tidyverse);
library(lmerTest);
library(MuMIn);
library(stargazer);
```

In [96]:

```
dat <- read.csv("Overconfidence_complete_data.csv")
#Multiply overplacement by 10 so interpret as percent not decimal (e.g. 10% not 0.1)
dat$op <- (dat$dplacement - .5)*100
dat$true_op <- dat$decileoc2*100
dat$pop <- as.factor(dat$sample)
dat$pop <- relevel(dat$pop, ref = "Euro_Canadian")
dat$zsd <- scale(dat$sd, center=T, scale=T)
dat$zage <- scale(dat$age, scale=TRUE, center = TRUE)
dat$avgfalseunique <- (dat$falseuniquecooperative + dat$falseuniquecreative + 
                       dat$falseuniqueindependent + dat$falseuniqueathletic + 
                       dat$falseuniquehumor + dat$falseuniquegetsalong + 
                       dat$falseuniqueconsiderate + dat$falseuniquehardworking + 
                       dat$falseuniquedependable + dat$falseuniqueattractive)/10
length(unique(dat$pid))
table(dat$sample)
```

319

```
Asian_Canadian  Euro_Canadian      Hong_Kong          Japan 
           252            264            436            324
```

In [97]:

```
dat.eurocan <- dat[dat$sample=="Euro_Canadian",]
dat.asiacan <- dat[dat$sample=="Asian_Canadian",]
dat.hk <- dat[dat$sample=="Hong_Kong",]
dat.jp <- dat[dat$sample=="Japan",]
```

# Correlations¶

In [98]:

```
dat.collapse <- dat %>%
select(pid, selfesteem, avgfalseunique, op, true_op, zsd) %>%
group_by(pid) %>%
summarise(selfesteem = mean(selfesteem), avgfalseunique = mean(avgfalseunique), 
op = mean(op), true_op = mean(true_op), zsd = mean(zsd))
glimpse(dat.collapse)
library("PerformanceAnalytics")
chart.Correlation(dat.collapse[c(2:6)], histogram=TRUE)
```

```
Observations: 319
Variables: 6
$ pid            <fct> 1076399, 1076418, 1076430, 1076512, 1076550, 1076571...
$ selfesteem     <dbl> 13, 17, 14, 11, 16, 17, 16, 14, 5, 12, 20, 15, 7, 4,...
$ avgfalseunique <dbl> 34.0, 63.0, 58.0, 38.5, 33.0, 70.0, 15.5, 41.0, 29.0...
$ op             <dbl> 9.50, 13.75, 6.00, -5.25, -2.25, 10.50, 26.75, 0.25,...
$ true_op        <dbl> 24.50, -1.25, 16.00, -25.25, -7.25, -9.50, 1.75, 20....
$ zsd            <dbl> -0.115924156, 0.302042025, -0.534284738, -0.68703681...
```

In [99]:

```
dat.collapse <- dat %>%
select(pid, sample, selfesteem, avgfalseunique, op, true_op, zsd) %>%
group_by(pid, sample) %>%
summarise(selfesteem = mean(selfesteem), avgfalseunique = mean(avgfalseunique), 
op = mean(op), true_op = mean(true_op), zsd = mean(zsd))
glimpse(dat.collapse)
library("PerformanceAnalytics")
dat.collapse.eurocan <- dat.collapse[dat.collapse$sample=="Euro_Canadian",]
dat.collapse.asiacan <- dat.collapse[dat.collapse$sample=="Asian_Canadian",]
dat.collapse.hk <- dat.collapse[dat.collapse$sample=="Hong_Kong",]
dat.collapse.jp <- dat.collapse[dat.collapse$sample=="Japan",]
chart.Correlation(dat.collapse.eurocan[c(3:7)], histogram=TRUE)
chart.Correlation(dat.collapse.asiacan[c(3:7)], histogram=TRUE)
chart.Correlation(dat.collapse.hk[c(3:7)], histogram=TRUE)
chart.Correlation(dat.collapse.jp[c(3:7)], histogram=TRUE)
```

```
Observations: 319
Variables: 7
$ pid            <fct> 1076399, 1076418, 1076430, 1076512, 1076550, 1076571...
$ sample         <fct> Japan, Japan, Japan, Japan, Japan, Japan, Japan, Jap...
$ selfesteem     <dbl> 13, 17, 14, 11, 16, 17, 16, 14, 5, 12, 20, 15, 7, 4,...
$ avgfalseunique <dbl> 34.0, 63.0, 58.0, 38.5, 33.0, 70.0, 15.5, 41.0, 29.0...
$ op             <dbl> 9.50, 13.75, 6.00, -5.25, -2.25, 10.50, 26.75, 0.25,...
$ true_op        <dbl> 24.50, -1.25, 16.00, -25.25, -7.25, -9.50, 1.75, 20....
$ zsd            <dbl> -0.115924156, 0.302042025, -0.534284738, -0.68703681...
```

# Overplacement¶

In [100]:

```
model.ec <- lmer(op ~ math + after + money + (1 | pid), data = dat.eurocan)
model.ac <- lmer(op ~ math + after + money + (1 | pid), data = dat.asiacan)
model.hk <- lmer(op ~ math + after + money + (1 | pid), data = dat.hk)
model.jp <- lmer(op ~ math + after + money + (1 | pid), data = dat.jp)
model.all <- lmer(op ~ pop*(math + after + money) + (1 | pid), data = dat)
class(model.ec) <- "lmerMod"
class(model.ac) <- "lmerMod"
class(model.hk) <- "lmerMod"
class(model.jp) <- "lmerMod"
class(model.all) <- "lmerMod"
stargazer(model.ec, model.ac, model.hk, model.jp, model.all, type="text", ci=TRUE, digits=2,
          star.char = c("+", "*", "**", "***"),
          star.cutoffs = c(0.1, 0.05, 0.01, 0.001),
          notes = c("+ p<0.1; * p<0.05; ** p<0.01; *** p<0.001"), 
          notes.append = F)
```

```
====================================================================================================
                                                    Dependent variable:                             
                        ----------------------------------------------------------------------------
                                                             op                                     
                              (1)             (2)            (3)            (4)            (5)      
----------------------------------------------------------------------------------------------------
popAsian_Canadian                                                                         -0.81     
                                                                                      (-8.45, 6.84) 
                                                                                                    
popHong_Kong                                                                              -2.13     
                                                                                      (-9.00, 4.74) 
                                                                                                    
popJapan                                                                                 -8.90*     
                                                                                     (-16.44, -1.36)
                                                                                                    
math                       -9.36***         -4.04*          -0.74          1.52         -9.36***    
                        (-13.31, -5.41) (-7.47, -0.61)  (-3.82, 2.33)  (-1.48, 4.51) (-13.04, -5.67)
                                                                                                    
after                      -6.78***        -8.07***        -4.28**         -1.98        -6.78***    
                        (-10.73, -2.83) (-11.50, -4.64) (-7.36, -1.21) (-4.97, 1.02) (-10.47, -3.10)
                                                                                                    
money                        1.86            2.92           -1.23          3.71           1.86      
                         (-3.66, 7.38)  (-5.23, 11.07)  (-5.81, 3.35)  (-1.75, 9.16)  (-4.55, 8.27) 
                                                                                                    
popAsian_Canadian:math                                                                    5.32*     
                                                                                      (0.04, 10.59) 
                                                                                                    
popHong_Kong:math                                                                        8.61***    
                                                                                      (3.94, 13.28) 
                                                                                                    
popJapan:math                                                                           10.87***    
                                                                                      (5.91, 15.84) 
                                                                                                    
popAsian_Canadian:after                                                                   -1.29     
                                                                                      (-6.56, 3.98) 
                                                                                                    
popHong_Kong:after                                                                        2.50      
                                                                                      (-2.17, 7.17) 
                                                                                                    
popJapan:after                                                                            4.80+     
                                                                                      (-0.16, 9.77) 
                                                                                                    
popAsian_Canadian:money                                                                   1.06      
                                                                                     (-8.01, 10.14) 
                                                                                                    
popHong_Kong:money                                                                        -3.09     
                                                                                     (-11.15, 4.97) 
                                                                                                    
popJapan:money                                                                            1.85      
                                                                                     (-6.77, 10.47) 
                                                                                                    
Constant                   11.14***        10.33***        9.00***         2.24         11.14***    
                         (6.01, 16.26)   (4.20, 16.46)  (5.17, 12.84)  (-2.47, 6.94)  (5.50, 16.77) 
                                                                                                    
----------------------------------------------------------------------------------------------------
Observations                  264             252            436            324           1,276     
Log Likelihood             -1,124.79       -1,066.25      -1,873.39      -1,348.43      -5,425.57   
Akaike Inf. Crit.          2,261.58        2,144.50        3,758.77      2,708.87       10,887.15   
Bayesian Inf. Crit.        2,283.03        2,165.67        3,783.24      2,731.55       10,979.88   
====================================================================================================
Note:                                                      + p<0.1; * p<0.05; ** p<0.01; *** p<0.001
```

In [101]:

```
r.squaredGLMM(model.ec)
r.squaredGLMM(model.ac)
r.squaredGLMM(model.hk)
r.squaredGLMM(model.jp)
r.squaredGLMM(model.all)
```

R2m
:   0.0952708188090371

R2c
:   0.255863190453937

R2m
:   0.0514013852209431

R2c
:   0.560374876119693

R2m
:   0.014424486509927

R2c
:   0.244184079217103

R2m
:   0.0163589604584296

R2c
:   0.365897151791537

R2m
:   0.0422683825013726

R2c
:   0.349737031567128

All populations are updating toward more accurate according to the traditional measure of overplacement. Does anyone seem more accurate?

## Before vs After Empathy¶

In [102]:

```
dat$before <- (dat$after + 1) %% 2 #Use modulo arithmetic to create a before variable
table(dat$before, dat$after)
dat.eurocan <- dat[dat$sample=="Euro_Canadian",]
dat.asiacan <- dat[dat$sample=="Asian_Canadian",]
dat.hk <- dat[dat$sample=="Hong_Kong",]
dat.jp <- dat[dat$sample=="Japan",]
```

```
      0   1
  0   0 638
  1 638   0
```

In [103]:

```
model.ec <- lmer(op ~ math + before + money + (1 | pid), data = dat.eurocan)
model.ac <- lmer(op ~ math + before + money + (1 | pid), data = dat.asiacan)
model.hk <- lmer(op ~ math + before + money + (1 | pid), data = dat.hk)
model.jp <- lmer(op ~ math + before + money + (1 | pid), data = dat.jp)
model.all <- lmer(op ~ pop*(math + before + money) + (1 | pid), data = dat)
summary(model.ec)
summary(model.hk)
```

```
Linear mixed model fit by REML. t-tests use Satterthwaite's method [
lmerModLmerTest]
Formula: op ~ math + before + money + (1 | pid)
   Data: dat.eurocan

REML criterion at convergence: 2249.6

Scaled residuals: 
     Min       1Q   Median       3Q      Max 
-2.81666 -0.53617  0.02082  0.64634  2.32133 

Random effects:
 Groups   Name        Variance Std.Dev.
 pid      (Intercept)  57.87    7.607  
 Residual             268.15   16.375  
Number of obs: 264, groups:  pid, 66

Fixed effects:
            Estimate Std. Error      df t value Pr(>|t|)    
(Intercept)    4.355      2.614 122.421   1.666 0.098313 .  
math          -9.356      2.016 196.000  -4.642 6.32e-06 ***
before         6.780      2.016 196.000   3.364 0.000925 ***
money          1.858      2.815  64.000   0.660 0.511739    
---
Signif. codes:  0 '***' 0.001 '**' 0.01 '*' 0.05 '.' 0.1 ' ' 1

Correlation of Fixed Effects:
       (Intr) math   before
math   -0.385              
before -0.385  0.000       
money  -0.653  0.000  0.000
```

```
Linear mixed model fit by REML. t-tests use Satterthwaite's method [
lmerModLmerTest]
Formula: op ~ math + before + money + (1 | pid)
   Data: dat.hk

REML criterion at convergence: 3746.8

Scaled residuals: 
     Min       1Q   Median       3Q      Max 
-2.69014 -0.61090  0.04322  0.60113  2.19496 

Random effects:
 Groups   Name        Variance Std.Dev.
 pid      (Intercept)  81.51    9.028  
 Residual             268.13   16.375  
Number of obs: 436, groups:  pid, 109

Fixed effects:
            Estimate Std. Error       df t value Pr(>|t|)   
(Intercept)   4.7206     1.9586 215.9965   2.410  0.01678 * 
math         -0.7431     1.5684 325.0000  -0.474  0.63596   
before        4.2844     1.5684 325.0000   2.732  0.00665 **
money        -1.2316     2.3372 107.0000  -0.527  0.59932   
---
Signif. codes:  0 '***' 0.001 '**' 0.01 '*' 0.05 '.' 0.1 ' ' 1

Correlation of Fixed Effects:
       (Intr) math   before
math   -0.400              
before -0.400  0.000       
money  -0.569  0.000  0.000
```

In [104]:

```
class(model.ec) <- "lmerMod"
class(model.ac) <- "lmerMod"
class(model.hk) <- "lmerMod"
class(model.jp) <- "lmerMod"
class(model.all) <- "lmerMod"
stargazer(model.ec, model.ac, model.hk, model.jp, model.all, type="text", ci=TRUE, digits=2,
          star.char = c("+", "*", "**", "***"),
          star.cutoffs = c(0.1, 0.05, 0.01, 0.001),
          notes = c("+ p<0.1; * p<0.05; ** p<0.01; *** p<0.001"), 
          notes.append = F)
```

```
===================================================================================================
                                                    Dependent variable:                            
                         --------------------------------------------------------------------------
                                                             op                                    
                               (1)            (2)            (3)           (4)            (5)      
---------------------------------------------------------------------------------------------------
popAsian_Canadian                                                                        -2.10     
                                                                                     (-9.74, 5.55) 
                                                                                                   
popHong_Kong                                                                             0.37      
                                                                                     (-6.50, 7.23) 
                                                                                                   
popJapan                                                                                 -4.09     
                                                                                    (-11.64, 3.45) 
                                                                                                   
math                        -9.36***         -4.04*         -0.74         1.52         -9.36***    
                         (-13.31, -5.41) (-7.47, -0.61) (-3.82, 2.33) (-1.48, 4.51) (-13.04, -5.67)
                                                                                                   
before                       6.78***        8.07***        4.28**         1.98          6.78***    
                          (2.83, 10.73)  (4.64, 11.50)  (1.21, 7.36)  (-1.02, 4.97)  (3.10, 10.47) 
                                                                                                   
money                         1.86            2.92          -1.23         3.71           1.86      
                          (-3.66, 7.38)  (-5.23, 11.07) (-5.81, 3.35) (-1.75, 9.16)  (-4.55, 8.27) 
                                                                                                   
popAsian_Canadian:math                                                                   5.32*     
                                                                                     (0.04, 10.59) 
                                                                                                   
popHong_Kong:math                                                                       8.61***    
                                                                                     (3.94, 13.28) 
                                                                                                   
popJapan:math                                                                          10.87***    
                                                                                     (5.91, 15.84) 
                                                                                                   
popAsian_Canadian:before                                                                 1.29      
                                                                                     (-3.98, 6.56) 
                                                                                                   
popHong_Kong:before                                                                      -2.50     
                                                                                     (-7.17, 2.17) 
                                                                                                   
popJapan:before                                                                         -4.80+     
                                                                                     (-9.77, 0.16) 
                                                                                                   
popAsian_Canadian:money                                                                  1.06      
                                                                                    (-8.01, 10.14) 
                                                                                                   
popHong_Kong:money                                                                       -3.09     
                                                                                    (-11.15, 4.97) 
                                                                                                   
popJapan:money                                                                           1.85      
                                                                                    (-6.77, 10.47) 
                                                                                                   
Constant                      4.36+           2.26          4.72*         0.26           4.36      
                          (-0.77, 9.48)  (-3.87, 8.38)  (0.88, 8.56)  (-4.44, 4.97)  (-1.28, 9.99) 
                                                                                                   
---------------------------------------------------------------------------------------------------
Observations                   264            252            436           324           1,276     
Log Likelihood              -1,124.79      -1,066.25      -1,873.39     -1,348.43      -5,425.57   
Akaike Inf. Crit.           2,261.58        2,144.50      3,758.77      2,708.87       10,887.15   
Bayesian Inf. Crit.         2,283.03        2,165.67      3,783.24      2,731.55       10,979.88   
===================================================================================================
Note:                                                     + p<0.1; * p<0.05; ** p<0.01; *** p<0.001
```

Let's do the same thing with the Math test (unincentived)

## Before vs After Math¶

In [105]:

```
dat$empathy <- (dat$math + 1) %% 2 #Use modulo arithmetic to create a before variable
table(dat$empathy, dat$math)
dat.eurocan <- dat[dat$sample=="Euro_Canadian",]
dat.asiacan <- dat[dat$sample=="Asian_Canadian",]
dat.hk <- dat[dat$sample=="Hong_Kong",]
dat.jp <- dat[dat$sample=="Japan",]
```

```
      0   1
  0   0 638
  1 638   0
```

### Before¶

In [106]:

```
model.ec <- lmer(op ~ empathy + after + money + (1 | pid), data = dat.eurocan)
model.ac <- lmer(op ~ empathy + after + money + (1 | pid), data = dat.asiacan)
model.hk <- lmer(op ~ empathy + after + money + (1 | pid), data = dat.hk)
model.jp <- lmer(op ~ empathy + after + money + (1 | pid), data = dat.jp)
model.all <- lmer(op ~ pop*(empathy + after + money) + (1 | pid), data = dat)
summary(model.ec)
summary(model.ac)
summary(model.hk)
summary(model.jp)
```

```
Linear mixed model fit by REML. t-tests use Satterthwaite's method [
lmerModLmerTest]
Formula: op ~ empathy + after + money + (1 | pid)
   Data: dat.eurocan

REML criterion at convergence: 2249.6

Scaled residuals: 
     Min       1Q   Median       3Q      Max 
-2.81666 -0.53617  0.02082  0.64634  2.32133 

Random effects:
 Groups   Name        Variance Std.Dev.
 pid      (Intercept)  57.87    7.607  
 Residual             268.15   16.375  
Number of obs: 264, groups:  pid, 66

Fixed effects:
            Estimate Std. Error      df t value Pr(>|t|)    
(Intercept)    1.779      2.614 122.421   0.681 0.497409    
empathy        9.356      2.016 196.000   4.642 6.32e-06 ***
after         -6.780      2.016 196.000  -3.364 0.000925 ***
money          1.858      2.815  64.000   0.660 0.511739    
---
Signif. codes:  0 '***' 0.001 '**' 0.01 '*' 0.05 '.' 0.1 ' ' 1

Correlation of Fixed Effects:
        (Intr) empthy after 
empathy -0.385              
after   -0.385  0.000       
money   -0.653  0.000  0.000
```

```
Linear mixed model fit by REML. t-tests use Satterthwaite's method [
lmerModLmerTest]
Formula: op ~ empathy + after + money + (1 | pid)
   Data: dat.asiacan

REML criterion at convergence: 2132.5

Scaled residuals: 
     Min       1Q   Median       3Q      Max 
-2.71702 -0.46458  0.01253  0.56226  1.95448 

Random effects:
 Groups   Name        Variance Std.Dev.
 pid      (Intercept) 223.6    14.95   
 Residual             193.1    13.90   
Number of obs: 252, groups:  pid, 63

Fixed effects:
            Estimate Std. Error      df t value Pr(>|t|)    
(Intercept)    6.289      3.126  84.851   2.012   0.0474 *  
empathy        4.040      1.751 187.000   2.307   0.0221 *  
after         -8.071      1.751 187.000  -4.610 7.45e-06 ***
money          2.919      4.160  61.000   0.702   0.4855    
---
Signif. codes:  0 '***' 0.001 '**' 0.01 '*' 0.05 '.' 0.1 ' ' 1

Correlation of Fixed Effects:
        (Intr) empthy after 
empathy -0.280              
after   -0.280  0.000       
money   -0.634  0.000  0.000
```

```
Linear mixed model fit by REML. t-tests use Satterthwaite's method [
lmerModLmerTest]
Formula: op ~ empathy + after + money + (1 | pid)
   Data: dat.hk

REML criterion at convergence: 3746.8

Scaled residuals: 
     Min       1Q   Median       3Q      Max 
-2.69014 -0.61090  0.04322  0.60113  2.19496 

Random effects:
 Groups   Name        Variance Std.Dev.
 pid      (Intercept)  81.51    9.028  
 Residual             268.13   16.375  
Number of obs: 436, groups:  pid, 109

Fixed effects:
            Estimate Std. Error       df t value Pr(>|t|)    
(Intercept)   8.2619     1.9586 215.9965   4.218 3.62e-05 ***
empathy       0.7431     1.5684 325.0000   0.474  0.63596    
after        -4.2844     1.5684 325.0000  -2.732  0.00665 ** 
money        -1.2316     2.3372 107.0000  -0.527  0.59932    
---
Signif. codes:  0 '***' 0.001 '**' 0.01 '*' 0.05 '.' 0.1 ' ' 1

Correlation of Fixed Effects:
        (Intr) empthy after 
empathy -0.400              
after   -0.400  0.000       
money   -0.569  0.000  0.000
```

```
Linear mixed model fit by REML. t-tests use Satterthwaite's method [
lmerModLmerTest]
Formula: op ~ empathy + after + money + (1 | pid)
   Data: dat.jp

REML criterion at convergence: 2696.9

Scaled residuals: 
     Min       1Q   Median       3Q      Max 
-2.25066 -0.64224  0.02222  0.55639  2.46217 

Random effects:
 Groups   Name        Variance Std.Dev.
 pid      (Intercept) 104.3    10.21   
 Residual             189.3    13.76   
Number of obs: 324, groups:  pid, 81

Fixed effects:
            Estimate Std. Error      df t value Pr(>|t|)
(Intercept)    3.754      2.401 121.697   1.564    0.120
empathy       -1.519      1.529 241.000  -0.993    0.322
after         -1.975      1.529 241.000  -1.292    0.198
money          3.706      2.785  79.000   1.331    0.187

Correlation of Fixed Effects:
        (Intr) empthy after 
empathy -0.318              
after   -0.318  0.000       
money   -0.687  0.000  0.000
```

In [107]:

```
class(model.ec) <- "lmerMod"
class(model.ac) <- "lmerMod"
class(model.hk) <- "lmerMod"
class(model.jp) <- "lmerMod"
class(model.all) <- "lmerMod"
stargazer(model.ec, model.ac, model.hk, model.jp, model.all, type="text", ci=TRUE, digits=2,
          star.char = c("+", "*", "**", "***"),
          star.cutoffs = c(0.1, 0.05, 0.01, 0.001),
          notes = c("+ p<0.1; * p<0.05; ** p<0.01; *** p<0.001"), 
          notes.append = F)
```

```
======================================================================================================
                                                      Dependent variable:                             
                          ----------------------------------------------------------------------------
                                                               op                                     
                                (1)             (2)            (3)            (4)            (5)      
------------------------------------------------------------------------------------------------------
popAsian_Canadian                                                                           4.51      
                                                                                       (-3.14, 12.15) 
                                                                                                      
popHong_Kong                                                                                6.48+     
                                                                                       (-0.39, 13.35) 
                                                                                                      
popJapan                                                                                    1.98      
                                                                                        (-5.57, 9.52) 
                                                                                                      
empathy                       9.36***          4.04*           0.74          -1.52         9.36***    
                           (5.41, 13.31)   (0.61, 7.47)   (-2.33, 3.82)  (-4.51, 1.48)  (5.67, 13.04) 
                                                                                                      
after                        -6.78***        -8.07***        -4.28**         -1.98        -6.78***    
                          (-10.73, -2.83) (-11.50, -4.64) (-7.36, -1.21) (-4.97, 1.02) (-10.47, -3.10)
                                                                                                      
money                          1.86            2.92           -1.23          3.71           1.86      
                           (-3.66, 7.38)  (-5.23, 11.07)  (-5.81, 3.35)  (-1.75, 9.16)  (-4.55, 8.27) 
                                                                                                      
popAsian_Canadian:empathy                                                                  -5.32*     
                                                                                       (-10.59, -0.04)
                                                                                                      
popHong_Kong:empathy                                                                      -8.61***    
                                                                                       (-13.28, -3.94)
                                                                                                      
popJapan:empathy                                                                          -10.87***   
                                                                                       (-15.84, -5.91)
                                                                                                      
popAsian_Canadian:after                                                                     -1.29     
                                                                                        (-6.56, 3.98) 
                                                                                                      
popHong_Kong:after                                                                          2.50      
                                                                                        (-2.17, 7.17) 
                                                                                                      
popJapan:after                                                                              4.80+     
                                                                                        (-0.16, 9.77) 
                                                                                                      
popAsian_Canadian:money                                                                     1.06      
                                                                                       (-8.01, 10.14) 
                                                                                                      
popHong_Kong:money                                                                          -3.09     
                                                                                       (-11.15, 4.97) 
                                                                                                      
popJapan:money                                                                              1.85      
                                                                                       (-6.77, 10.47) 
                                                                                                      
Constant                       1.78            6.29*         8.26***         3.75           1.78      
                           (-3.34, 6.90)   (0.16, 12.42)  (4.42, 12.10)  (-0.95, 8.46)  (-3.85, 7.41) 
                                                                                                      
------------------------------------------------------------------------------------------------------
Observations                    264             252            436            324           1,276     
Log Likelihood               -1,124.79       -1,066.25      -1,873.39      -1,348.43      -5,425.57   
Akaike Inf. Crit.            2,261.58        2,144.50        3,758.77      2,708.87       10,887.15   
Bayesian Inf. Crit.          2,283.03        2,165.67        3,783.24      2,731.55       10,979.88   
======================================================================================================
Note:                                                        + p<0.1; * p<0.05; ** p<0.01; *** p<0.001
```

### After¶

In [108]:

```
model.ec <- lmer(op ~ empathy + before + money + (1 | pid), data = dat.eurocan)
model.ac <- lmer(op ~ empathy + before + money + (1 | pid), data = dat.asiacan)
model.hk <- lmer(op ~ empathy + before + money + (1 | pid), data = dat.hk)
model.jp <- lmer(op ~ empathy + before + money + (1 | pid), data = dat.jp)
model.all <- lmer(op ~ pop*(empathy + before + money) + (1 | pid), data = dat)
summary(model.ec)
summary(model.ac)
summary(model.hk)
summary(model.jp)
```

```
Linear mixed model fit by REML. t-tests use Satterthwaite's method [
lmerModLmerTest]
Formula: op ~ empathy + before + money + (1 | pid)
   Data: dat.eurocan

REML criterion at convergence: 2249.6

Scaled residuals: 
     Min       1Q   Median       3Q      Max 
-2.81666 -0.53617  0.02082  0.64634  2.32133 

Random effects:
 Groups   Name        Variance Std.Dev.
 pid      (Intercept)  57.87    7.607  
 Residual             268.15   16.375  
Number of obs: 264, groups:  pid, 66

Fixed effects:
            Estimate Std. Error      df t value Pr(>|t|)    
(Intercept)   -5.001      2.614 122.421  -1.913 0.058116 .  
empathy        9.356      2.016 196.000   4.642 6.32e-06 ***
before         6.780      2.016 196.000   3.364 0.000925 ***
money          1.858      2.815  64.000   0.660 0.511739    
---
Signif. codes:  0 '***' 0.001 '**' 0.01 '*' 0.05 '.' 0.1 ' ' 1

Correlation of Fixed Effects:
        (Intr) empthy before
empathy -0.385              
before  -0.385  0.000       
money   -0.653  0.000  0.000
```

```
Linear mixed model fit by REML. t-tests use Satterthwaite's method [
lmerModLmerTest]
Formula: op ~ empathy + before + money + (1 | pid)
   Data: dat.asiacan

REML criterion at convergence: 2132.5

Scaled residuals: 
     Min       1Q   Median       3Q      Max 
-2.71702 -0.46458  0.01253  0.56226  1.95448 

Random effects:
 Groups   Name        Variance Std.Dev.
 pid      (Intercept) 223.6    14.95   
 Residual             193.1    13.90   
Number of obs: 252, groups:  pid, 63

Fixed effects:
            Estimate Std. Error      df t value Pr(>|t|)    
(Intercept)   -1.783      3.126  84.851  -0.570   0.5700    
empathy        4.040      1.751 187.000   2.307   0.0221 *  
before         8.071      1.751 187.000   4.610 7.45e-06 ***
money          2.919      4.160  61.000   0.702   0.4855    
---
Signif. codes:  0 '***' 0.001 '**' 0.01 '*' 0.05 '.' 0.1 ' ' 1

Correlation of Fixed Effects:
        (Intr) empthy before
empathy -0.280              
before  -0.280  0.000       
money   -0.634  0.000  0.000
```

```
Linear mixed model fit by REML. t-tests use Satterthwaite's method [
lmerModLmerTest]
Formula: op ~ empathy + before + money + (1 | pid)
   Data: dat.hk

REML criterion at convergence: 3746.8

Scaled residuals: 
     Min       1Q   Median       3Q      Max 
-2.69014 -0.61090  0.04322  0.60113  2.19496 

Random effects:
 Groups   Name        Variance Std.Dev.
 pid      (Intercept)  81.51    9.028  
 Residual             268.13   16.375  
Number of obs: 436, groups:  pid, 109

Fixed effects:
            Estimate Std. Error       df t value Pr(>|t|)   
(Intercept)   3.9775     1.9586 215.9965   2.031  0.04350 * 
empathy       0.7431     1.5684 325.0000   0.474  0.63596   
before        4.2844     1.5684 325.0000   2.732  0.00665 **
money        -1.2316     2.3372 107.0000  -0.527  0.59932   
---
Signif. codes:  0 '***' 0.001 '**' 0.01 '*' 0.05 '.' 0.1 ' ' 1

Correlation of Fixed Effects:
        (Intr) empthy before
empathy -0.400              
before  -0.400  0.000       
money   -0.569  0.000  0.000
```

```
Linear mixed model fit by REML. t-tests use Satterthwaite's method [
lmerModLmerTest]
Formula: op ~ empathy + before + money + (1 | pid)
   Data: dat.jp

REML criterion at convergence: 2696.9

Scaled residuals: 
     Min       1Q   Median       3Q      Max 
-2.25066 -0.64224  0.02222  0.55639  2.46217 

Random effects:
 Groups   Name        Variance Std.Dev.
 pid      (Intercept) 104.3    10.21   
 Residual             189.3    13.76   
Number of obs: 324, groups:  pid, 81

Fixed effects:
            Estimate Std. Error      df t value Pr(>|t|)
(Intercept)    1.779      2.401 121.697   0.741    0.460
empathy       -1.519      1.529 241.000  -0.993    0.322
before         1.975      1.529 241.000   1.292    0.198
money          3.706      2.785  79.000   1.331    0.187

Correlation of Fixed Effects:
        (Intr) empthy before
empathy -0.318              
before  -0.318  0.000       
money   -0.687  0.000  0.000
```

In [109]:

```
class(model.ec) <- "lmerMod"
class(model.ac) <- "lmerMod"
class(model.hk) <- "lmerMod"
class(model.jp) <- "lmerMod"
class(model.all) <- "lmerMod"
stargazer(model.ec, model.ac, model.hk, model.jp, model.all, type="text", ci=TRUE, digits=2,
          star.char = c("+", "*", "**", "***"),
          star.cutoffs = c(0.1, 0.05, 0.01, 0.001),
          notes = c("+ p<0.1; * p<0.05; ** p<0.01; *** p<0.001"), 
          notes.append = F)
```

```
===================================================================================================
                                                     Dependent variable:                           
                          -------------------------------------------------------------------------
                                                             op                                    
                               (1)            (2)            (3)           (4)            (5)      
---------------------------------------------------------------------------------------------------
popAsian_Canadian                                                                        3.22      
                                                                                    (-4.43, 10.86) 
                                                                                                   
popHong_Kong                                                                             8.98*     
                                                                                     (2.11, 15.85) 
                                                                                                   
popJapan                                                                                 6.78+     
                                                                                    (-0.76, 14.32) 
                                                                                                   
empathy                      9.36***         4.04*          0.74          -1.52         9.36***    
                          (5.41, 13.31)   (0.61, 7.47)  (-2.33, 3.82) (-4.51, 1.48)  (5.67, 13.04) 
                                                                                                   
before                       6.78***        8.07***        4.28**         1.98          6.78***    
                          (2.83, 10.73)  (4.64, 11.50)  (1.21, 7.36)  (-1.02, 4.97)  (3.10, 10.47) 
                                                                                                   
money                          1.86           2.92          -1.23         3.71           1.86      
                          (-3.66, 7.38)  (-5.23, 11.07) (-5.81, 3.35) (-1.75, 9.16)  (-4.55, 8.27) 
                                                                                                   
popAsian_Canadian:empathy                                                               -5.32*     
                                                                                    (-10.59, -0.04)
                                                                                                   
popHong_Kong:empathy                                                                   -8.61***    
                                                                                    (-13.28, -3.94)
                                                                                                   
popJapan:empathy                                                                       -10.87***   
                                                                                    (-15.84, -5.91)
                                                                                                   
popAsian_Canadian:before                                                                 1.29      
                                                                                     (-3.98, 6.56) 
                                                                                                   
popHong_Kong:before                                                                      -2.50     
                                                                                     (-7.17, 2.17) 
                                                                                                   
popJapan:before                                                                         -4.80+     
                                                                                     (-9.77, 0.16) 
                                                                                                   
popAsian_Canadian:money                                                                  1.06      
                                                                                    (-8.01, 10.14) 
                                                                                                   
popHong_Kong:money                                                                       -3.09     
                                                                                    (-11.15, 4.97) 
                                                                                                   
popJapan:money                                                                           1.85      
                                                                                    (-6.77, 10.47) 
                                                                                                   
Constant                      -5.00+         -1.78          3.98*         1.78          -5.00+     
                          (-10.13, 0.12) (-7.91, 4.34)  (0.14, 7.82)  (-2.93, 6.48) (-10.63, 0.63) 
                                                                                                   
---------------------------------------------------------------------------------------------------
Observations                   264            252            436           324           1,276     
Log Likelihood              -1,124.79      -1,066.25      -1,873.39     -1,348.43      -5,425.57   
Akaike Inf. Crit.            2,261.58       2,144.50      3,758.77      2,708.87       10,887.15   
Bayesian Inf. Crit.          2,283.03       2,165.67      3,783.24      2,731.55       10,979.88   
===================================================================================================
Note:                                                     + p<0.1; * p<0.05; ** p<0.01; *** p<0.001
```

## Incentives¶

In [110]:

```
dat$tokens <- (dat$money + 1) %% 2 #Use modulo arithmetic to create a before variable
table(dat$tokens, dat$money)
dat.eurocan <- dat[dat$sample=="Euro_Canadian",]
dat.asiacan <- dat[dat$sample=="Asian_Canadian",]
dat.hk <- dat[dat$sample=="Hong_Kong",]
dat.jp <- dat[dat$sample=="Japan",]
```

```
      0   1
  0   0 680
  1 596   0
```

Once again, we are running the same regression model, just changing the reference group for convenience.

### Empathy¶

In [111]:

```
#Before Empathy
model.ec.be <- lmer(op ~ math + after + tokens + (1 | pid), data = dat.eurocan)
model.ac.be <- lmer(op ~ math + after + tokens + (1 | pid), data = dat.asiacan)
model.hk.be <- lmer(op ~ math + after + tokens + (1 | pid), data = dat.hk)
model.jp.be <- lmer(op ~ math + after + tokens + (1 | pid), data = dat.jp)
model.all.be <- lmer(op ~ pop*(math + after + tokens) + (1 | pid), data = dat)

#After Empathy
model.ec.ae <- lmer(op ~ math + before + tokens + (1 | pid), data = dat.eurocan)
model.ac.ae <- lmer(op ~ math + before + tokens + (1 | pid), data = dat.asiacan)
model.hk.ae <- lmer(op ~ math + before + tokens + (1 | pid), data = dat.hk)
model.jp.ae <- lmer(op ~ math + before + tokens + (1 | pid), data = dat.jp)
model.all.ae <- lmer(op ~ pop*(math + before + tokens) + (1 | pid), data = dat)
```

In [112]:

```
#Before Empathy
class(model.ec.be) <- "lmerMod"
class(model.ac.be) <- "lmerMod"
class(model.hk.be) <- "lmerMod"
class(model.jp.be) <- "lmerMod"
class(model.all.be) <- "lmerMod"
stargazer(model.ec.be, model.ac.be, model.hk.be, model.jp.be, model.all.be,
          type="text", ci=TRUE, digits=2,
          star.char = c("+", "*", "**", "***"),
          star.cutoffs = c(0.1, 0.05, 0.01, 0.001),
          notes = c("+ p<0.1; * p<0.05; ** p<0.01; *** p<0.001"), 
          notes.append = F)

#After Empathy
class(model.ec.ae) <- "lmerMod"
class(model.ac.ae) <- "lmerMod"
class(model.hk.ae) <- "lmerMod"
class(model.jp.ae) <- "lmerMod"
class(model.all.ae) <- "lmerMod"
stargazer(model.ec.ae, model.ac.ae, model.hk.ae, model.jp.ae, model.all.ae,
          type="text", ci=TRUE, digits=2,
          star.char = c("+", "*", "**", "***"),
          star.cutoffs = c(0.1, 0.05, 0.01, 0.001),
          notes = c("+ p<0.1; * p<0.05; ** p<0.01; *** p<0.001"), 
          notes.append = F)
```

```
=====================================================================================================
                                                     Dependent variable:                             
                         ----------------------------------------------------------------------------
                                                              op                                     
                               (1)             (2)            (3)            (4)            (5)      
-----------------------------------------------------------------------------------------------------
popAsian_Canadian                                                                          0.25      
                                                                                       (-6.94, 7.44) 
                                                                                                     
popHong_Kong                                                                               -5.22     
                                                                                      (-11.51, 1.07) 
                                                                                                     
popJapan                                                                                  -7.05*     
                                                                                      (-13.53, -0.57)
                                                                                                     
math                        -9.36***         -4.04*          -0.74          1.52         -9.36***    
                         (-13.31, -5.41) (-7.47, -0.61)  (-3.82, 2.33)  (-1.48, 4.51) (-13.04, -5.67)
                                                                                                     
after                       -6.78***        -8.07***        -4.28**         -1.98        -6.78***    
                         (-10.73, -2.83) (-11.50, -4.64) (-7.36, -1.21) (-4.97, 1.02) (-10.47, -3.10)
                                                                                                     
tokens                        -1.86           -2.92           1.23          -3.71          -1.86     
                          (-7.38, 3.66)  (-11.07, 5.23)  (-3.35, 5.81)  (-9.16, 1.75)  (-8.27, 4.55) 
                                                                                                     
popAsian_Canadian:math                                                                     5.32*     
                                                                                       (0.04, 10.59) 
                                                                                                     
popHong_Kong:math                                                                         8.61***    
                                                                                       (3.94, 13.28) 
                                                                                                     
popJapan:math                                                                            10.87***    
                                                                                       (5.91, 15.84) 
                                                                                                     
popAsian_Canadian:after                                                                    -1.29     
                                                                                       (-6.56, 3.98) 
                                                                                                     
popHong_Kong:after                                                                         2.50      
                                                                                       (-2.17, 7.17) 
                                                                                                     
popJapan:after                                                                             4.80+     
                                                                                       (-0.16, 9.77) 
                                                                                                     
popAsian_Canadian:tokens                                                                   -1.06     
                                                                                      (-10.14, 8.01) 
                                                                                                     
popHong_Kong:tokens                                                                        3.09      
                                                                                      (-4.97, 11.15) 
                                                                                                     
popJapan:tokens                                                                            -1.85     
                                                                                      (-10.47, 6.77) 
                                                                                                     
Constant                    12.99***        13.25***        7.77***        5.94**        12.99***    
                          (8.54, 17.44)   (6.87, 19.63)  (3.81, 11.74)  (1.86, 10.02)  (8.20, 17.79) 
                                                                                                     
-----------------------------------------------------------------------------------------------------
Observations                   264             252            436            324           1,276     
Log Likelihood              -1,124.79       -1,066.25      -1,873.39      -1,348.43      -5,425.57   
Akaike Inf. Crit.           2,261.58        2,144.50        3,758.77      2,708.87       10,887.15   
Bayesian Inf. Crit.         2,283.03        2,165.67        3,783.24      2,731.55       10,979.88   
=====================================================================================================
Note:                                                       + p<0.1; * p<0.05; ** p<0.01; *** p<0.001

===================================================================================================
                                                    Dependent variable:                            
                         --------------------------------------------------------------------------
                                                             op                                    
                               (1)            (2)            (3)           (4)            (5)      
---------------------------------------------------------------------------------------------------
popAsian_Canadian                                                                        -1.04     
                                                                                     (-8.23, 6.15) 
                                                                                                   
popHong_Kong                                                                             -2.72     
                                                                                     (-9.01, 3.57) 
                                                                                                   
popJapan                                                                                 -2.25     
                                                                                     (-8.73, 4.24) 
                                                                                                   
math                        -9.36***         -4.04*         -0.74         1.52         -9.36***    
                         (-13.31, -5.41) (-7.47, -0.61) (-3.82, 2.33) (-1.48, 4.51) (-13.04, -5.67)
                                                                                                   
before                       6.78***        8.07***        4.28**         1.98          6.78***    
                          (2.83, 10.73)  (4.64, 11.50)  (1.21, 7.36)  (-1.02, 4.97)  (3.10, 10.47) 
                                                                                                   
tokens                        -1.86          -2.92          1.23          -3.71          -1.86     
                          (-7.38, 3.66)  (-11.07, 5.23) (-3.35, 5.81) (-9.16, 1.75)  (-8.27, 4.55) 
                                                                                                   
popAsian_Canadian:math                                                                   5.32*     
                                                                                     (0.04, 10.59) 
                                                                                                   
popHong_Kong:math                                                                       8.61***    
                                                                                     (3.94, 13.28) 
                                                                                                   
popJapan:math                                                                          10.87***    
                                                                                     (5.91, 15.84) 
                                                                                                   
popAsian_Canadian:before                                                                 1.29      
                                                                                     (-3.98, 6.56) 
                                                                                                   
popHong_Kong:before                                                                      -2.50     
                                                                                     (-7.17, 2.17) 
                                                                                                   
popJapan:before                                                                         -4.80+     
                                                                                     (-9.77, 0.16) 
                                                                                                   
popAsian_Canadian:tokens                                                                 -1.06     
                                                                                    (-10.14, 8.01) 
                                                                                                   
popHong_Kong:tokens                                                                      3.09      
                                                                                    (-4.97, 11.15) 
                                                                                                   
popJapan:tokens                                                                          -1.85     
                                                                                    (-10.47, 6.77) 
                                                                                                   
Constant                     6.21**           5.18          3.49+         3.97+          6.21*     
                          (1.76, 10.66)  (-1.20, 11.56) (-0.47, 7.45) (-0.11, 8.04)  (1.42, 11.01) 
                                                                                                   
---------------------------------------------------------------------------------------------------
Observations                   264            252            436           324           1,276     
Log Likelihood              -1,124.79      -1,066.25      -1,873.39     -1,348.43      -5,425.57   
Akaike Inf. Crit.           2,261.58        2,144.50      3,758.77      2,708.87       10,887.15   
Bayesian Inf. Crit.         2,283.03        2,165.67      3,783.24      2,731.55       10,979.88   
===================================================================================================
Note:                                                     + p<0.1; * p<0.05; ** p<0.01; *** p<0.001
```

In [113]:

```
save.image()
```

# True Overplacement¶

## Before vs After Empathy¶

In [147]:

```
model.ec <- lmer(true_op ~ math + after + money + (1 | pid), data = dat.eurocan)
model.ac <- lmer(true_op ~ math + after + money + (1 | pid), data = dat.asiacan)
model.hk <- lmer(true_op ~ math + after + money + (1 | pid), data = dat.hk)
model.jp <- lmer(true_op ~ math + after + money + (1 | pid), data = dat.jp)
model.all <- lmer(true_op ~ pop*(math + after + money) + (1 | pid), data = dat)
summary(model.jp)
summary(model.all)
```

```
Linear mixed model fit by REML. t-tests use Satterthwaite's method [
lmerModLmerTest]
Formula: true_op ~ math + after + money + (1 | pid)
   Data: dat.jp

REML criterion at convergence: 3017.3

Scaled residuals: 
    Min      1Q  Median      3Q     Max 
-3.1714 -0.6258  0.0113  0.6821  2.5400 

Random effects:
 Groups   Name        Variance Std.Dev.
 pid      (Intercept) 277.7    16.66   
 Residual             517.6    22.75   
Number of obs: 324, groups:  pid, 81

Fixed effects:
            Estimate Std. Error      df t value Pr(>|t|)  
(Intercept)   -2.523      3.941 122.528  -0.640   0.5233  
math           1.642      2.528 241.000   0.650   0.5166  
after         -1.975      2.528 241.000  -0.781   0.4353  
money         11.424      4.563  79.000   2.504   0.0143 *
---
Signif. codes:  0 '***' 0.001 '**' 0.01 '*' 0.05 '.' 0.1 ' ' 1

Correlation of Fixed Effects:
      (Intr) math   after 
math  -0.321              
after -0.321  0.000       
money -0.686  0.000  0.000
```

```
Correlation matrix not shown by default, as p = 16 > 12.
Use print(obj, correlation=TRUE)  or
	 vcov(obj)	 if you need it
```

```
Linear mixed model fit by REML. t-tests use Satterthwaite's method [
lmerModLmerTest]
Formula: true_op ~ pop * (math + after + money) + (1 | pid)
   Data: dat

REML criterion at convergence: 11804.2

Scaled residuals: 
    Min      1Q  Median      3Q     Max 
-3.2776 -0.6289  0.0254  0.6457  2.6211 

Random effects:
 Groups   Name        Variance Std.Dev.
 pid      (Intercept) 276.0    16.61   
 Residual             481.4    21.94   
Number of obs: 1276, groups:  pid, 319

Fixed effects:
                        Estimate Std. Error       df t value Pr(>|t|)   
(Intercept)              10.4945     4.3463 468.8392   2.415  0.01613 * 
popHong_Kong             -4.2128     5.2963 485.6648  -0.795  0.42676   
popAsian_Canadian         0.5986     5.8924 492.6821   0.102  0.91912   
popJapan                -13.0172     5.8199 471.3073  -2.237  0.02578 * 
math                     -7.6894     2.7008 949.0000  -2.847  0.00451 **
after                    -6.7803     2.7008 949.0000  -2.510  0.01222 * 
money                     3.1654     5.0150 311.0000   0.631  0.52839   
popHong_Kong:math         7.1298     3.4222 949.0000   2.083  0.03748 * 
popAsian_Canadian:math    3.3323     3.8648 949.0000   0.862  0.38879   
popJapan:math             9.3314     3.6384 949.0000   2.565  0.01048 * 
popHong_Kong:after        2.4959     3.4222 949.0000   0.729  0.46598   
popAsian_Canadian:after  -1.2911     3.8648 949.0000  -0.334  0.73840   
popJapan:after            4.8050     3.6384 949.0000   1.321  0.18694   
popHong_Kong:money        0.6384     6.3028 311.0000   0.101  0.91938   
popAsian_Canadian:money  -3.3525     7.0972 311.0000  -0.472  0.63699   
popJapan:money            8.2584     6.7392 311.0000   1.225  0.22134   
---
Signif. codes:  0 '***' 0.001 '**' 0.01 '*' 0.05 '.' 0.1 ' ' 1
```

In [148]:

```
class(model.ec) <- "lmerMod"
class(model.ac) <- "lmerMod"
class(model.hk) <- "lmerMod"
class(model.jp) <- "lmerMod"
class(model.all) <- "lmerMod"
stargazer(model.ec, model.ac, model.hk, model.jp, model.all, type="text", ci=TRUE, digits=2,
          star.char = c("+", "*", "**", "***"),
          star.cutoffs = c(0.1, 0.05, 0.01, 0.001),
          notes = c("+ p<0.1; * p<0.05; ** p<0.01; *** p<0.001"), 
          notes.append = F)
```

```
=====================================================================================================
                                                     Dependent variable:                             
                        -----------------------------------------------------------------------------
                                                           true_op                                   
                              (1)             (2)            (3)            (4)             (5)      
-----------------------------------------------------------------------------------------------------
popHong_Kong                                                                               -4.21     
                                                                                      (-14.59, 6.17) 
                                                                                                     
popAsian_Canadian                                                                          0.60      
                                                                                      (-10.95, 12.15)
                                                                                                     
popJapan                                                                                  -13.02*    
                                                                                      (-24.42, -1.61)
                                                                                                     
math                        -7.69**         -4.36+          -0.56           1.64          -7.69**    
                        (-13.46, -1.92)  (-9.47, 0.75)  (-4.46, 3.34)  (-3.31, 6.60)  (-12.98, -2.40)
                                                                                                     
after                       -6.78*          -8.07**         -4.28*         -1.98          -6.78*     
                        (-12.55, -1.01) (-13.18, -2.96) (-8.18, -0.39) (-6.93, 2.98)  (-12.07, -1.49)
                                                                                                     
money                        3.17            -0.19           3.80          11.42*          3.17      
                        (-7.28, 13.61)  (-10.25, 9.87)  (-3.20, 10.81) (2.48, 20.37)  (-6.66, 12.99) 
                                                                                                     
popHong_Kong:math                                                                          7.13*     
                                                                                       (0.42, 13.84) 
                                                                                                     
popAsian_Canadian:math                                                                     3.33      
                                                                                      (-4.24, 10.91) 
                                                                                                     
popJapan:math                                                                              9.33*     
                                                                                       (2.20, 16.46) 
                                                                                                     
popHong_Kong:after                                                                         2.50      
                                                                                       (-4.21, 9.20) 
                                                                                                     
popAsian_Canadian:after                                                                    -1.29     
                                                                                       (-8.87, 6.28) 
                                                                                                     
popJapan:after                                                                             4.80      
                                                                                      (-2.33, 11.94) 
                                                                                                     
popHong_Kong:money                                                                         0.64      
                                                                                      (-11.71, 12.99)
                                                                                                     
popAsian_Canadian:money                                                                    -3.35     
                                                                                      (-17.26, 10.56)
                                                                                                     
popJapan:money                                                                             8.26      
                                                                                      (-4.95, 21.47) 
                                                                                                     
Constant                    10.49*          11.09**         6.28*          -2.52          10.49*     
                         (1.40, 19.59)   (3.27, 18.92)  (0.71, 11.85)  (-10.25, 5.20)  (1.98, 19.01) 
                                                                                                     
-----------------------------------------------------------------------------------------------------
Observations                  264             252            436            324            1,276     
Log Likelihood             -1,239.73       -1,153.47      -1,996.09      -1,508.67       -5,902.11   
Akaike Inf. Crit.          2,491.47        2,318.95        4,004.18       3,029.34       11,840.22   
Bayesian Inf. Crit.        2,512.92        2,340.12        4,028.64       3,052.02       11,932.94   
=====================================================================================================
Note:                                                       + p<0.1; * p<0.05; ** p<0.01; *** p<0.001
```

In [116]:

```
r.squaredGLMM(model.ec)
r.squaredGLMM(model.ac)
r.squaredGLMM(model.hk)
r.squaredGLMM(model.jp)
r.squaredGLMM(model.all)
```

R2m
:   0.0318034890761515

R2c
:   0.368775825725988

R2m
:   0.0279364934021131

R2c
:   0.434070058898977

R2m
:   0.0122133912544224

R2c
:   0.36505165981768

R2m
:   0.0401415747230589

R2c
:   0.375286964350647

R2m
:   0.0278983818979029

R2c
:   0.382081700379482

The Japanese are accurate and significantly less overconfident in the base treatment (empathy, before the test, unincentivized) based on the intercept. The All Populations regression also reveals that they are significantly less overconfident than the Euro Canadians. How do they compare to the East Asian Canadians and Hong Kong Chinese? The answer is in the same regression model - the coefficient is the difference between the Japanese coefficient and the Asian Canadian and Hong Kong coefficients. From this we can easily see that they're 0.60 more underconfident than the Asian Canadians (relative to their difference from the Euro Canadians) and this is obviously going to be significant. But it's easier to calculate the significance by just changing the reference category.

Note: This is the same regression model as above, but with a different reference category.

In [150]:

```
model.ec <- lmer(true_op ~ math + before + money + (1 | pid), data = dat.eurocan)
model.ac <- lmer(true_op ~ math + before + money + (1 | pid), data = dat.asiacan)
model.hk <- lmer(true_op ~ math + before + money + (1 | pid), data = dat.hk)
model.jp <- lmer(true_op ~ math + before + money + (1 | pid), data = dat.jp)
model.all <- lmer(true_op ~ pop*(math + before + money) + (1 | pid), data = dat)
summary(model.ec)
summary(model.ac)
summary(model.hk)
summary(model.jp)
summary(model.all)
```

```
Linear mixed model fit by REML. t-tests use Satterthwaite's method [
lmerModLmerTest]
Formula: true_op ~ math + before + money + (1 | pid)
   Data: dat.eurocan

REML criterion at convergence: 2479.5

Scaled residuals: 
    Min      1Q  Median      3Q     Max 
-2.5391 -0.6792  0.1237  0.6489  2.3604 

Random effects:
 Groups   Name        Variance Std.Dev.
 pid      (Intercept) 304.9    17.46   
 Residual             571.1    23.90   
Number of obs: 264, groups:  pid, 66

Fixed effects:
            Estimate Std. Error      df t value Pr(>|t|)   
(Intercept)    3.714      4.642  98.182   0.800  0.42554   
math          -7.689      2.942 196.000  -2.614  0.00965 **
before         6.780      2.942 196.000   2.305  0.02222 * 
money          3.165      5.330  64.000   0.594  0.55470   
---
Signif. codes:  0 '***' 0.001 '**' 0.01 '*' 0.05 '.' 0.1 ' ' 1

Correlation of Fixed Effects:
       (Intr) math   before
math   -0.317              
before -0.317  0.000       
money  -0.696  0.000  0.000
```

```
Linear mixed model fit by REML. t-tests use Satterthwaite's method [
lmerModLmerTest]
Formula: true_op ~ math + before + money + (1 | pid)
   Data: dat.asiacan

REML criterion at convergence: 2306.9

Scaled residuals: 
     Min       1Q   Median       3Q      Max 
-2.08816 -0.70328  0.01549  0.67265  2.29343 

Random effects:
 Groups   Name        Variance Std.Dev.
 pid      (Intercept) 307.1    17.52   
 Residual             428.0    20.69   
Number of obs: 252, groups:  pid, 63

Fixed effects:
            Estimate Std. Error       df t value Pr(>|t|)   
(Intercept)   3.0216     3.9932  96.1910   0.757  0.45108   
math         -4.3571     2.6063 187.0000  -1.672  0.09625 . 
before        8.0714     2.6063 187.0000   3.097  0.00226 **
money        -0.1871     5.1335  61.0000  -0.036  0.97104   
---
Signif. codes:  0 '***' 0.001 '**' 0.01 '*' 0.05 '.' 0.1 ' ' 1

Correlation of Fixed Effects:
       (Intr) math   before
math   -0.326              
before -0.326  0.000       
money  -0.612  0.000  0.000
```

```
Linear mixed model fit by REML. t-tests use Satterthwaite's method [
lmerModLmerTest]
Formula: true_op ~ math + before + money + (1 | pid)
   Data: dat.hk

REML criterion at convergence: 3992.2

Scaled residuals: 
     Min       1Q   Median       3Q      Max 
-2.60221 -0.58207 -0.00065  0.61574  2.61473 

Random effects:
 Groups   Name        Variance Std.Dev.
 pid      (Intercept) 239.7    15.48   
 Residual             431.3    20.77   
Number of obs: 436, groups:  pid, 109

Fixed effects:
            Estimate Std. Error       df t value Pr(>|t|)  
(Intercept)   1.9973     2.8416 181.4264   0.703    0.483  
math         -0.5596     1.9892 325.0000  -0.281    0.779  
before        4.2844     1.9892 325.0000   2.154    0.032 *
money         3.8038     3.5747 107.0000   1.064    0.290  
---
Signif. codes:  0 '***' 0.001 '**' 0.01 '*' 0.05 '.' 0.1 ' ' 1

Correlation of Fixed Effects:
       (Intr) math   before
math   -0.350              
before -0.350  0.000       
money  -0.600  0.000  0.000
```

```
Linear mixed model fit by REML. t-tests use Satterthwaite's method [
lmerModLmerTest]
Formula: true_op ~ math + before + money + (1 | pid)
   Data: dat.jp

REML criterion at convergence: 3017.3

Scaled residuals: 
    Min      1Q  Median      3Q     Max 
-3.1714 -0.6258  0.0113  0.6821  2.5400 

Random effects:
 Groups   Name        Variance Std.Dev.
 pid      (Intercept) 277.7    16.66   
 Residual             517.6    22.75   
Number of obs: 324, groups:  pid, 81

Fixed effects:
            Estimate Std. Error      df t value Pr(>|t|)  
(Intercept)   -4.498      3.941 122.528  -1.141   0.2559  
math           1.642      2.528 241.000   0.650   0.5166  
before         1.975      2.528 241.000   0.781   0.4353  
money         11.424      4.563  79.000   2.504   0.0143 *
---
Signif. codes:  0 '***' 0.001 '**' 0.01 '*' 0.05 '.' 0.1 ' ' 1

Correlation of Fixed Effects:
       (Intr) math   before
math   -0.321              
before -0.321  0.000       
money  -0.686  0.000  0.000
```

```
Correlation matrix not shown by default, as p = 16 > 12.
Use print(obj, correlation=TRUE)  or
	 vcov(obj)	 if you need it
```

```
Linear mixed model fit by REML. t-tests use Satterthwaite's method [
lmerModLmerTest]
Formula: true_op ~ pop * (math + before + money) + (1 | pid)
   Data: dat

REML criterion at convergence: 11804.2

Scaled residuals: 
    Min      1Q  Median      3Q     Max 
-3.2776 -0.6289  0.0254  0.6457  2.6211 

Random effects:
 Groups   Name        Variance Std.Dev.
 pid      (Intercept) 276.0    16.61   
 Residual             481.4    21.94   
Number of obs: 1276, groups:  pid, 319

Fixed effects:
                         Estimate Std. Error       df t value Pr(>|t|)   
(Intercept)                3.7142     4.3463 468.8392   0.855  0.39323   
popHong_Kong              -1.7169     5.2963 485.6648  -0.324  0.74595   
popAsian_Canadian         -0.6925     5.8924 492.6821  -0.118  0.90649   
popJapan                  -8.2122     5.8199 471.3073  -1.411  0.15889   
math                      -7.6894     2.7008 949.0000  -2.847  0.00451 **
before                     6.7803     2.7008 949.0000   2.510  0.01222 * 
money                      3.1654     5.0150 311.0000   0.631  0.52839   
popHong_Kong:math          7.1298     3.4222 949.0000   2.083  0.03748 * 
popAsian_Canadian:math     3.3323     3.8648 949.0000   0.862  0.38879   
popJapan:math              9.3314     3.6384 949.0000   2.565  0.01048 * 
popHong_Kong:before       -2.4959     3.4222 949.0000  -0.729  0.46598   
popAsian_Canadian:before   1.2911     3.8648 949.0000   0.334  0.73840   
popJapan:before           -4.8050     3.6384 949.0000  -1.321  0.18694   
popHong_Kong:money         0.6384     6.3028 311.0000   0.101  0.91938   
popAsian_Canadian:money   -3.3525     7.0972 311.0000  -0.472  0.63699   
popJapan:money             8.2584     6.7392 311.0000   1.225  0.22134   
---
Signif. codes:  0 '***' 0.001 '**' 0.01 '*' 0.05 '.' 0.1 ' ' 1
```

In [117]:

```
dat$pop <- relevel(dat$pop, ref = "Asian_Canadian")
model.all.ea <- lmer(true_op ~ pop*(math + after + money) + (1 | pid), data = dat)

dat$pop <- relevel(dat$pop, ref = "Hong_Kong")
model.all.hk <- lmer(true_op ~ pop*(math + after + money) + (1 | pid), data = dat)
summary(model.all.ea)
summary(model.all.hk)
class(model.all.ea) <- "lmerMod"
class(model.all.hk) <- "lmerMod"

stargazer(model.all.ea, model.all.hk, type="text", ci=TRUE, digits=2,
          star.char = c("+", "*", "**", "***"),
          star.cutoffs = c(0.1, 0.05, 0.01, 0.001),
          notes = c("+ p<0.1; * p<0.05; ** p<0.01; *** p<0.001"), 
          notes.append = F)
dat$pop <- relevel(dat$pop, ref = "Euro_Canadian")
```

```
Correlation matrix not shown by default, as p = 16 > 12.
Use print(obj, correlation=TRUE)  or
	 vcov(obj)	 if you need it
```

```
Linear mixed model fit by REML. t-tests use Satterthwaite's method [
lmerModLmerTest]
Formula: true_op ~ pop * (math + after + money) + (1 | pid)
   Data: dat

REML criterion at convergence: 11804.2

Scaled residuals: 
    Min      1Q  Median      3Q     Max 
-3.2776 -0.6289  0.0254  0.6457  2.6211 

Random effects:
 Groups   Name        Variance Std.Dev.
 pid      (Intercept) 276.0    16.61   
 Residual             481.4    21.94   
Number of obs: 1276, groups:  pid, 319

Fixed effects:
                       Estimate Std. Error       df t value Pr(>|t|)   
(Intercept)             11.0931     3.9787 523.0258   2.788  0.00550 **
popEuro_Canadian        -0.5986     5.8924 492.6820  -0.102  0.91912   
popHong_Kong            -4.8114     4.9992 522.8919  -0.962  0.33627   
popJapan               -13.6158     5.5508 498.7091  -2.453  0.01451 * 
math                    -4.3571     2.7644 949.0000  -1.576  0.11532   
after                   -8.0714     2.7644 949.0000  -2.920  0.00359 **
money                   -0.1871     5.0219 311.0000  -0.037  0.97030   
popEuro_Canadian:math   -3.3323     3.8648 949.0000  -0.862  0.38879   
popHong_Kong:math        3.7975     3.4726 949.0000   1.094  0.27442   
popJapan:math            5.9991     3.6858 949.0000   1.628  0.10394   
popEuro_Canadian:after   1.2911     3.8648 949.0000   0.334  0.73840   
popHong_Kong:after       3.7870     3.4726 949.0000   1.091  0.27574   
popJapan:after           6.0961     3.6858 949.0000   1.654  0.09847 . 
popEuro_Canadian:money   3.3525     7.0972 311.0000   0.472  0.63699   
popHong_Kong:money       3.9909     6.3083 311.0000   0.633  0.52743   
popJapan:money          11.6109     6.7443 311.0000   1.722  0.08614 . 
---
Signif. codes:  0 '***' 0.001 '**' 0.01 '*' 0.05 '.' 0.1 ' ' 1
```

```
Correlation matrix not shown by default, as p = 16 > 12.
Use print(obj, correlation=TRUE)  or
	 vcov(obj)	 if you need it
```

```
Linear mixed model fit by REML. t-tests use Satterthwaite's method [
lmerModLmerTest]
Formula: true_op ~ pop * (math + after + money) + (1 | pid)
   Data: dat

REML criterion at convergence: 11804.2

Scaled residuals: 
    Min      1Q  Median      3Q     Max 
-3.2776 -0.6289  0.0254  0.6457  2.6211 

Random effects:
 Groups   Name        Variance Std.Dev.
 pid      (Intercept) 276.0    16.61   
 Residual             481.4    21.94   
Number of obs: 1276, groups:  pid, 319

Fixed effects:
                        Estimate Std. Error       df t value Pr(>|t|)  
(Intercept)               6.2817     3.0268 522.6606   2.075   0.0384 *
popAsian_Canadian         4.8114     4.9992 522.8919   0.962   0.3363  
popEuro_Canadian          4.2128     5.2963 485.6648   0.795   0.4268  
popJapan                 -8.8044     4.9135 492.0968  -1.792   0.0738 .
math                     -0.5596     2.1016 949.0000  -0.266   0.7901  
after                    -4.2844     2.1016 949.0000  -2.039   0.0418 *
money                     3.8038     3.8176 311.0000   0.996   0.3198  
popAsian_Canadian:math   -3.7975     3.4726 949.0000  -1.094   0.2744  
popEuro_Canadian:math    -7.1298     3.4222 949.0000  -2.083   0.0375 *
popJapan:math             2.2016     3.2188 949.0000   0.684   0.4941  
popAsian_Canadian:after  -3.7870     3.4726 949.0000  -1.091   0.2757  
popEuro_Canadian:after   -2.4959     3.4222 949.0000  -0.729   0.4660  
popJapan:after            2.3091     3.2188 949.0000   0.717   0.4733  
popAsian_Canadian:money  -3.9909     6.3083 311.0000  -0.633   0.5274  
popEuro_Canadian:money   -0.6384     6.3028 311.0000  -0.101   0.9194  
popJapan:money            7.6200     5.9026 311.0000   1.291   0.1977  
---
Signif. codes:  0 '***' 0.001 '**' 0.01 '*' 0.05 '.' 0.1 ' ' 1
```

```
==================================================================
                                   Dependent variable:            
                        ------------------------------------------
                                         true_op                  
                                 (1)                  (2)         
------------------------------------------------------------------
popAsian_Canadian                                     4.81        
                                                 (-4.99, 14.61)   
                                                                  
popEuro_Canadian                -0.60                 4.21        
                           (-12.15, 10.95)       (-6.17, 14.59)   
                                                                  
popHong_Kong                    -4.81                             
                           (-14.61, 4.99)                         
                                                                  
popJapan                       -13.62*               -8.80+       
                           (-24.50, -2.74)       (-18.43, 0.83)   
                                                                  
math                            -4.36                -0.56        
                            (-9.78, 1.06)        (-4.68, 3.56)    
                                                                  
after                          -8.07**               -4.28*       
                           (-13.49, -2.65)       (-8.40, -0.17)   
                                                                  
money                           -0.19                 3.80        
                           (-10.03, 9.66)        (-3.68, 11.29)   
                                                                  
popAsian_Canadian:math                               -3.80        
                                                 (-10.60, 3.01)   
                                                                  
popEuro_Canadian:math           -3.33                -7.13*       
                           (-10.91, 4.24)       (-13.84, -0.42)   
                                                                  
popHong_Kong:math               3.80                              
                           (-3.01, 10.60)                         
                                                                  
popJapan:math                   6.00                  2.20        
                           (-1.23, 13.22)        (-4.11, 8.51)    
                                                                  
popAsian_Canadian:after                              -3.79        
                                                 (-10.59, 3.02)   
                                                                  
popEuro_Canadian:after          1.29                 -2.50        
                            (-6.28, 8.87)        (-9.20, 4.21)    
                                                                  
popHong_Kong:after              3.79                              
                           (-3.02, 10.59)                         
                                                                  
popJapan:after                  6.10+                 2.31        
                           (-1.13, 13.32)        (-4.00, 8.62)    
                                                                  
popAsian_Canadian:money                              -3.99        
                                                 (-16.35, 8.37)   
                                                                  
popEuro_Canadian:money          3.35                 -0.64        
                           (-10.56, 17.26)      (-12.99, 11.71)   
                                                                  
popHong_Kong:money              3.99                              
                           (-8.37, 16.35)                         
                                                                  
popJapan:money                 11.61+                 7.62        
                           (-1.61, 24.83)        (-3.95, 19.19)   
                                                                  
Constant                       11.09**               6.28*        
                            (3.29, 18.89)        (0.35, 12.21)    
                                                                  
------------------------------------------------------------------
Observations                    1,276                1,276        
Log Likelihood                -5,902.11            -5,902.11      
Akaike Inf. Crit.             11,840.22            11,840.22      
Bayesian Inf. Crit.           11,932.94            11,932.94      
==================================================================
Note:                    + p<0.1; * p<0.05; ** p<0.01; *** p<0.001
```

All groups become more accurate after the empathy test with no incentives. What is their after empathy test unincentivized value and is it satistically indistinguishable from zero? To do this, we can look to the regressions above, but once again, it's easier to run the *same* regression model with the reference category as "After" (rather than "Before").

In [118]:

```
dat$before <- (dat$after + 1) %% 2 #Use modulo arithmetic to create a before variable
table(dat$before, dat$after)
dat.eurocan <- dat[dat$sample=="Euro_Canadian",]
dat.asiacan <- dat[dat$sample=="Asian_Canadian",]
dat.hk <- dat[dat$sample=="Hong_Kong",]
dat.jp <- dat[dat$sample=="Japan",]
```

```
      0   1
  0   0 638
  1 638   0
```

In [119]:

```
model.ec <- lmer(true_op ~ math + before + money + (1 | pid), data = dat.eurocan)
model.ac <- lmer(true_op ~ math + before + money + (1 | pid), data = dat.asiacan)
model.hk <- lmer(true_op ~ math + before + money + (1 | pid), data = dat.hk)
model.jp <- lmer(true_op ~ math + before + money + (1 | pid), data = dat.jp)
model.all <- lmer(true_op ~ pop*(math + before + money) + (1 | pid), data = dat)
summary(model.all)
```

```
Correlation matrix not shown by default, as p = 16 > 12.
Use print(obj, correlation=TRUE)  or
	 vcov(obj)	 if you need it
```

```
Linear mixed model fit by REML. t-tests use Satterthwaite's method [
lmerModLmerTest]
Formula: true_op ~ pop * (math + before + money) + (1 | pid)
   Data: dat

REML criterion at convergence: 11804.2

Scaled residuals: 
    Min      1Q  Median      3Q     Max 
-3.2776 -0.6289  0.0254  0.6457  2.6211 

Random effects:
 Groups   Name        Variance Std.Dev.
 pid      (Intercept) 276.0    16.61   
 Residual             481.4    21.94   
Number of obs: 1276, groups:  pid, 319

Fixed effects:
                         Estimate Std. Error       df t value Pr(>|t|)   
(Intercept)                3.7142     4.3463 468.8392   0.855  0.39323   
popHong_Kong              -1.7169     5.2963 485.6648  -0.324  0.74595   
popAsian_Canadian         -0.6925     5.8924 492.6821  -0.118  0.90649   
popJapan                  -8.2122     5.8199 471.3073  -1.411  0.15889   
math                      -7.6894     2.7008 949.0000  -2.847  0.00451 **
before                     6.7803     2.7008 949.0000   2.510  0.01222 * 
money                      3.1654     5.0150 311.0000   0.631  0.52839   
popHong_Kong:math          7.1298     3.4222 949.0000   2.083  0.03748 * 
popAsian_Canadian:math     3.3323     3.8648 949.0000   0.862  0.38879   
popJapan:math              9.3314     3.6384 949.0000   2.565  0.01048 * 
popHong_Kong:before       -2.4959     3.4222 949.0000  -0.729  0.46598   
popAsian_Canadian:before   1.2911     3.8648 949.0000   0.334  0.73840   
popJapan:before           -4.8050     3.6384 949.0000  -1.321  0.18694   
popHong_Kong:money         0.6384     6.3028 311.0000   0.101  0.91938   
popAsian_Canadian:money   -3.3525     7.0972 311.0000  -0.472  0.63699   
popJapan:money             8.2584     6.7392 311.0000   1.225  0.22134   
---
Signif. codes:  0 '***' 0.001 '**' 0.01 '*' 0.05 '.' 0.1 ' ' 1
```

In [120]:

```
class(model.ec) <- "lmerMod"
class(model.ac) <- "lmerMod"
class(model.hk) <- "lmerMod"
class(model.jp) <- "lmerMod"
class(model.all) <- "lmerMod"
stargazer(model.ec, model.ac, model.hk, model.jp, model.all, type="text", ci=TRUE, digits=2,
          star.char = c("+", "*", "**", "***"),
          star.cutoffs = c(0.1, 0.05, 0.01, 0.001),
          notes = c("+ p<0.1; * p<0.05; ** p<0.01; *** p<0.001"), 
          notes.append = F)
```

```
=====================================================================================================
                                                     Dependent variable:                             
                         ----------------------------------------------------------------------------
                                                           true_op                                   
                               (1)            (2)            (3)            (4)             (5)      
-----------------------------------------------------------------------------------------------------
popHong_Kong                                                                               -1.72     
                                                                                      (-12.10, 8.66) 
                                                                                                     
popAsian_Canadian                                                                          -0.69     
                                                                                      (-12.24, 10.86)
                                                                                                     
popJapan                                                                                   -8.21     
                                                                                      (-19.62, 3.19) 
                                                                                                     
math                         -7.69**         -4.36+         -0.56           1.64          -7.69**    
                         (-13.46, -1.92) (-9.47, 0.75)  (-4.46, 3.34)  (-3.31, 6.60)  (-12.98, -2.40)
                                                                                                     
before                        6.78*          8.07**         4.28*           1.98           6.78*     
                          (1.01, 12.55)  (2.96, 13.18)   (0.39, 8.18)  (-2.98, 6.93)   (1.49, 12.07) 
                                                                                                     
money                         3.17           -0.19           3.80          11.42*          3.17      
                         (-7.28, 13.61)  (-10.25, 9.87) (-3.20, 10.81) (2.48, 20.37)  (-6.66, 12.99) 
                                                                                                     
popHong_Kong:math                                                                          7.13*     
                                                                                       (0.42, 13.84) 
                                                                                                     
popAsian_Canadian:math                                                                     3.33      
                                                                                      (-4.24, 10.91) 
                                                                                                     
popJapan:math                                                                              9.33*     
                                                                                       (2.20, 16.46) 
                                                                                                     
popHong_Kong:before                                                                        -2.50     
                                                                                       (-9.20, 4.21) 
                                                                                                     
popAsian_Canadian:before                                                                   1.29      
                                                                                       (-6.28, 8.87) 
                                                                                                     
popJapan:before                                                                            -4.80     
                                                                                      (-11.94, 2.33) 
                                                                                                     
popHong_Kong:money                                                                         0.64      
                                                                                      (-11.71, 12.99)
                                                                                                     
popAsian_Canadian:money                                                                    -3.35     
                                                                                      (-17.26, 10.56)
                                                                                                     
popJapan:money                                                                             8.26      
                                                                                      (-4.95, 21.47) 
                                                                                                     
Constant                      3.71            3.02           2.00          -4.50           3.71      
                         (-5.38, 12.81)  (-4.80, 10.85) (-3.57, 7.57)  (-12.22, 3.23) (-4.80, 12.23) 
                                                                                                     
-----------------------------------------------------------------------------------------------------
Observations                   264            252            436            324            1,276     
Log Likelihood              -1,239.73      -1,153.47      -1,996.09      -1,508.67       -5,902.11   
Akaike Inf. Crit.           2,491.47        2,318.95       4,004.18       3,029.34       11,840.22   
Bayesian Inf. Crit.         2,512.92        2,340.12       4,028.64       3,052.02       11,932.94   
=====================================================================================================
Note:                                                       + p<0.1; * p<0.05; ** p<0.01; *** p<0.001
```

## Before vs After Math¶

In [121]:

```
dat$empathy <- (dat$math + 1) %% 2 #Use modulo arithmetic to create a before variable
table(dat$empathy, dat$math)
dat.eurocan <- dat[dat$sample=="Euro_Canadian",]
dat.asiacan <- dat[dat$sample=="Asian_Canadian",]
dat.hk <- dat[dat$sample=="Hong_Kong",]
dat.jp <- dat[dat$sample=="Japan",]
```

```
      0   1
  0   0 638
  1 638   0
```

### Before¶

In [122]:

```
model.ec <- lmer(true_op ~ empathy + after + money + (1 | pid), data = dat.eurocan)
model.ac <- lmer(true_op ~ empathy + after + money + (1 | pid), data = dat.asiacan)
model.hk <- lmer(true_op ~ empathy + after + money + (1 | pid), data = dat.hk)
model.jp <- lmer(true_op ~ empathy + after + money + (1 | pid), data = dat.jp)
model.all <- lmer(true_op ~ pop*(empathy + after + money) + (1 | pid), data = dat)
summary(model.ec)
summary(model.ac)
summary(model.hk)
summary(model.jp)
summary(model.all)
```

```
Linear mixed model fit by REML. t-tests use Satterthwaite's method [
lmerModLmerTest]
Formula: true_op ~ empathy + after + money + (1 | pid)
   Data: dat.eurocan

REML criterion at convergence: 2479.5

Scaled residuals: 
    Min      1Q  Median      3Q     Max 
-2.5391 -0.6792  0.1237  0.6489  2.3604 

Random effects:
 Groups   Name        Variance Std.Dev.
 pid      (Intercept) 304.9    17.46   
 Residual             571.1    23.90   
Number of obs: 264, groups:  pid, 66

Fixed effects:
            Estimate Std. Error      df t value Pr(>|t|)   
(Intercept)    2.805      4.642  98.182   0.604  0.54702   
empathy        7.689      2.942 196.000   2.614  0.00965 **
after         -6.780      2.942 196.000  -2.305  0.02222 * 
money          3.165      5.330  64.000   0.594  0.55470   
---
Signif. codes:  0 '***' 0.001 '**' 0.01 '*' 0.05 '.' 0.1 ' ' 1

Correlation of Fixed Effects:
        (Intr) empthy after 
empathy -0.317              
after   -0.317  0.000       
money   -0.696  0.000  0.000
```

```
Linear mixed model fit by REML. t-tests use Satterthwaite's method [
lmerModLmerTest]
Formula: true_op ~ empathy + after + money + (1 | pid)
   Data: dat.asiacan

REML criterion at convergence: 2306.9

Scaled residuals: 
     Min       1Q   Median       3Q      Max 
-2.08816 -0.70328  0.01549  0.67265  2.29343 

Random effects:
 Groups   Name        Variance Std.Dev.
 pid      (Intercept) 307.1    17.52   
 Residual             428.0    20.69   
Number of obs: 252, groups:  pid, 63

Fixed effects:
            Estimate Std. Error       df t value Pr(>|t|)   
(Intercept)   6.7359     3.9932  96.1910   1.687  0.09487 . 
empathy       4.3571     2.6063 187.0000   1.672  0.09625 . 
after        -8.0714     2.6063 187.0000  -3.097  0.00226 **
money        -0.1871     5.1335  61.0000  -0.036  0.97104   
---
Signif. codes:  0 '***' 0.001 '**' 0.01 '*' 0.05 '.' 0.1 ' ' 1

Correlation of Fixed Effects:
        (Intr) empthy after 
empathy -0.326              
after   -0.326  0.000       
money   -0.612  0.000  0.000
```

```
Linear mixed model fit by REML. t-tests use Satterthwaite's method [
lmerModLmerTest]
Formula: true_op ~ empathy + after + money + (1 | pid)
   Data: dat.hk

REML criterion at convergence: 3992.2

Scaled residuals: 
     Min       1Q   Median       3Q      Max 
-2.60221 -0.58207 -0.00065  0.61574  2.61473 

Random effects:
 Groups   Name        Variance Std.Dev.
 pid      (Intercept) 239.7    15.48   
 Residual             431.3    20.77   
Number of obs: 436, groups:  pid, 109

Fixed effects:
            Estimate Std. Error       df t value Pr(>|t|)  
(Intercept)   5.7220     2.8416 181.4264   2.014   0.0455 *
empathy       0.5596     1.9892 325.0000   0.281   0.7786  
after        -4.2844     1.9892 325.0000  -2.154   0.0320 *
money         3.8038     3.5747 107.0000   1.064   0.2897  
---
Signif. codes:  0 '***' 0.001 '**' 0.01 '*' 0.05 '.' 0.1 ' ' 1

Correlation of Fixed Effects:
        (Intr) empthy after 
empathy -0.350              
after   -0.350  0.000       
money   -0.600  0.000  0.000
```

```
Linear mixed model fit by REML. t-tests use Satterthwaite's method [
lmerModLmerTest]
Formula: true_op ~ empathy + after + money + (1 | pid)
   Data: dat.jp

REML criterion at convergence: 3017.3

Scaled residuals: 
    Min      1Q  Median      3Q     Max 
-3.1714 -0.6258  0.0113  0.6821  2.5400 

Random effects:
 Groups   Name        Variance Std.Dev.
 pid      (Intercept) 277.7    16.66   
 Residual             517.6    22.75   
Number of obs: 324, groups:  pid, 81

Fixed effects:
            Estimate Std. Error       df t value Pr(>|t|)  
(Intercept)  -0.8808     3.9409 122.5284  -0.223   0.8235  
empathy      -1.6420     2.5279 241.0000  -0.650   0.5166  
after        -1.9753     2.5279 241.0000  -0.781   0.4353  
money        11.4238     4.5625  79.0000   2.504   0.0143 *
---
Signif. codes:  0 '***' 0.001 '**' 0.01 '*' 0.05 '.' 0.1 ' ' 1

Correlation of Fixed Effects:
        (Intr) empthy after 
empathy -0.321              
after   -0.321  0.000       
money   -0.686  0.000  0.000
```

```
Correlation matrix not shown by default, as p = 16 > 12.
Use print(obj, correlation=TRUE)  or
	 vcov(obj)	 if you need it
```

```
Linear mixed model fit by REML. t-tests use Satterthwaite's method [
lmerModLmerTest]
Formula: true_op ~ pop * (empathy + after + money) + (1 | pid)
   Data: dat

REML criterion at convergence: 11804.2

Scaled residuals: 
    Min      1Q  Median      3Q     Max 
-3.2776 -0.6289  0.0254  0.6457  2.6211 

Random effects:
 Groups   Name        Variance Std.Dev.
 pid      (Intercept) 276.0    16.61   
 Residual             481.4    21.94   
Number of obs: 1276, groups:  pid, 319

Fixed effects:
                          Estimate Std. Error       df t value Pr(>|t|)   
(Intercept)                 2.8051     4.3463 468.8392   0.645  0.51898   
popHong_Kong                2.9170     5.2963 485.6648   0.551  0.58206   
popAsian_Canadian           3.9309     5.8924 492.6821   0.667  0.50502   
popJapan                   -3.6858     5.8199 471.3073  -0.633  0.52684   
empathy                     7.6894     2.7008 949.0000   2.847  0.00451 **
after                      -6.7803     2.7008 949.0000  -2.510  0.01222 * 
money                       3.1654     5.0150 311.0000   0.631  0.52839   
popHong_Kong:empathy       -7.1298     3.4222 949.0000  -2.083  0.03748 * 
popAsian_Canadian:empathy  -3.3323     3.8648 949.0000  -0.862  0.38879   
popJapan:empathy           -9.3314     3.6384 949.0000  -2.565  0.01048 * 
popHong_Kong:after          2.4959     3.4222 949.0000   0.729  0.46598   
popAsian_Canadian:after    -1.2911     3.8648 949.0000  -0.334  0.73840   
popJapan:after              4.8050     3.6384 949.0000   1.321  0.18694   
popHong_Kong:money          0.6384     6.3028 311.0000   0.101  0.91938   
popAsian_Canadian:money    -3.3525     7.0972 311.0000  -0.472  0.63699   
popJapan:money              8.2584     6.7392 311.0000   1.225  0.22134   
---
Signif. codes:  0 '***' 0.001 '**' 0.01 '*' 0.05 '.' 0.1 ' ' 1
```

In [123]:

```
class(model.ec) <- "lmerMod"
class(model.ac) <- "lmerMod"
class(model.hk) <- "lmerMod"
class(model.jp) <- "lmerMod"
class(model.all) <- "lmerMod"
stargazer(model.ec, model.ac, model.hk, model.jp, model.all, type="text", ci=TRUE, digits=2,
          star.char = c("+", "*", "**", "***"),
          star.cutoffs = c(0.1, 0.05, 0.01, 0.001),
          notes = c("+ p<0.1; * p<0.05; ** p<0.01; *** p<0.001"), 
          notes.append = F)
```

```
======================================================================================================
                                                      Dependent variable:                             
                          ----------------------------------------------------------------------------
                                                            true_op                                   
                                (1)             (2)            (3)            (4)            (5)      
------------------------------------------------------------------------------------------------------
popHong_Kong                                                                                2.92      
                                                                                       (-7.46, 13.30) 
                                                                                                      
popAsian_Canadian                                                                           3.93      
                                                                                       (-7.62, 15.48) 
                                                                                                      
popJapan                                                                                    -3.69     
                                                                                       (-15.09, 7.72) 
                                                                                                      
empathy                       7.69**           4.36+           0.56          -1.64         7.69**     
                           (1.92, 13.46)   (-0.75, 9.47)  (-3.34, 4.46)  (-6.60, 3.31)  (2.40, 12.98) 
                                                                                                      
after                         -6.78*          -8.07**         -4.28*         -1.98         -6.78*     
                          (-12.55, -1.01) (-13.18, -2.96) (-8.18, -0.39) (-6.93, 2.98) (-12.07, -1.49)
                                                                                                      
money                          3.17            -0.19           3.80         11.42*          3.17      
                          (-7.28, 13.61)  (-10.25, 9.87)  (-3.20, 10.81) (2.48, 20.37) (-6.66, 12.99) 
                                                                                                      
popHong_Kong:empathy                                                                       -7.13*     
                                                                                       (-13.84, -0.42)
                                                                                                      
popAsian_Canadian:empathy                                                                   -3.33     
                                                                                       (-10.91, 4.24) 
                                                                                                      
popJapan:empathy                                                                           -9.33*     
                                                                                       (-16.46, -2.20)
                                                                                                      
popHong_Kong:after                                                                          2.50      
                                                                                        (-4.21, 9.20) 
                                                                                                      
popAsian_Canadian:after                                                                     -1.29     
                                                                                        (-8.87, 6.28) 
                                                                                                      
popJapan:after                                                                              4.80      
                                                                                       (-2.33, 11.94) 
                                                                                                      
popHong_Kong:money                                                                          0.64      
                                                                                       (-11.71, 12.99)
                                                                                                      
popAsian_Canadian:money                                                                     -3.35     
                                                                                       (-17.26, 10.56)
                                                                                                      
popJapan:money                                                                              8.26      
                                                                                       (-4.95, 21.47) 
                                                                                                      
Constant                       2.81            6.74+          5.72*          -0.88          2.81      
                          (-6.29, 11.90)  (-1.09, 14.56)  (0.15, 11.29)  (-8.60, 6.84) (-5.71, 11.32) 
                                                                                                      
------------------------------------------------------------------------------------------------------
Observations                    264             252            436            324           1,276     
Log Likelihood               -1,239.73       -1,153.47      -1,996.09      -1,508.67      -5,902.11   
Akaike Inf. Crit.            2,491.47        2,318.95        4,004.18      3,029.34       11,840.22   
Bayesian Inf. Crit.          2,512.92        2,340.12        4,028.64      3,052.02       11,932.94   
======================================================================================================
Note:                                                        + p<0.1; * p<0.05; ** p<0.01; *** p<0.001
```

### After¶

In [124]:

```
model.ec <- lmer(true_op ~ empathy + before + money + (1 | pid), data = dat.eurocan)
model.ac <- lmer(true_op ~ empathy + before + money + (1 | pid), data = dat.asiacan)
model.hk <- lmer(true_op ~ empathy + before + money + (1 | pid), data = dat.hk)
model.jp <- lmer(true_op ~ empathy + before + money + (1 | pid), data = dat.jp)
model.all <- lmer(true_op ~ pop*(empathy + before + money) + (1 | pid), data = dat)
summary(model.ec)
summary(model.ac)
summary(model.hk)
summary(model.jp)
```

```
Linear mixed model fit by REML. t-tests use Satterthwaite's method [
lmerModLmerTest]
Formula: true_op ~ empathy + before + money + (1 | pid)
   Data: dat.eurocan

REML criterion at convergence: 2479.5

Scaled residuals: 
    Min      1Q  Median      3Q     Max 
-2.5391 -0.6792  0.1237  0.6489  2.3604 

Random effects:
 Groups   Name        Variance Std.Dev.
 pid      (Intercept) 304.9    17.46   
 Residual             571.1    23.90   
Number of obs: 264, groups:  pid, 66

Fixed effects:
            Estimate Std. Error      df t value Pr(>|t|)   
(Intercept)   -3.975      4.642  98.182  -0.856  0.39385   
empathy        7.689      2.942 196.000   2.614  0.00965 **
before         6.780      2.942 196.000   2.305  0.02222 * 
money          3.165      5.330  64.000   0.594  0.55470   
---
Signif. codes:  0 '***' 0.001 '**' 0.01 '*' 0.05 '.' 0.1 ' ' 1

Correlation of Fixed Effects:
        (Intr) empthy before
empathy -0.317              
before  -0.317  0.000       
money   -0.696  0.000  0.000
```

```
Linear mixed model fit by REML. t-tests use Satterthwaite's method [
lmerModLmerTest]
Formula: true_op ~ empathy + before + money + (1 | pid)
   Data: dat.asiacan

REML criterion at convergence: 2306.9

Scaled residuals: 
     Min       1Q   Median       3Q      Max 
-2.08816 -0.70328  0.01549  0.67265  2.29343 

Random effects:
 Groups   Name        Variance Std.Dev.
 pid      (Intercept) 307.1    17.52   
 Residual             428.0    20.69   
Number of obs: 252, groups:  pid, 63

Fixed effects:
            Estimate Std. Error       df t value Pr(>|t|)   
(Intercept)  -1.3355     3.9932  96.1910  -0.334  0.73877   
empathy       4.3571     2.6063 187.0000   1.672  0.09625 . 
before        8.0714     2.6063 187.0000   3.097  0.00226 **
money        -0.1871     5.1335  61.0000  -0.036  0.97104   
---
Signif. codes:  0 '***' 0.001 '**' 0.01 '*' 0.05 '.' 0.1 ' ' 1

Correlation of Fixed Effects:
        (Intr) empthy before
empathy -0.326              
before  -0.326  0.000       
money   -0.612  0.000  0.000
```

```
Linear mixed model fit by REML. t-tests use Satterthwaite's method [
lmerModLmerTest]
Formula: true_op ~ empathy + before + money + (1 | pid)
   Data: dat.hk

REML criterion at convergence: 3992.2

Scaled residuals: 
     Min       1Q   Median       3Q      Max 
-2.60221 -0.58207 -0.00065  0.61574  2.61473 

Random effects:
 Groups   Name        Variance Std.Dev.
 pid      (Intercept) 239.7    15.48   
 Residual             431.3    20.77   
Number of obs: 436, groups:  pid, 109

Fixed effects:
            Estimate Std. Error       df t value Pr(>|t|)  
(Intercept)   1.4376     2.8416 181.4264   0.506    0.614  
empathy       0.5596     1.9892 325.0000   0.281    0.779  
before        4.2844     1.9892 325.0000   2.154    0.032 *
money         3.8038     3.5747 107.0000   1.064    0.290  
---
Signif. codes:  0 '***' 0.001 '**' 0.01 '*' 0.05 '.' 0.1 ' ' 1

Correlation of Fixed Effects:
        (Intr) empthy before
empathy -0.350              
before  -0.350  0.000       
money   -0.600  0.000  0.000
```

```
Linear mixed model fit by REML. t-tests use Satterthwaite's method [
lmerModLmerTest]
Formula: true_op ~ empathy + before + money + (1 | pid)
   Data: dat.jp

REML criterion at convergence: 3017.3

Scaled residuals: 
    Min      1Q  Median      3Q     Max 
-3.1714 -0.6258  0.0113  0.6821  2.5400 

Random effects:
 Groups   Name        Variance Std.Dev.
 pid      (Intercept) 277.7    16.66   
 Residual             517.6    22.75   
Number of obs: 324, groups:  pid, 81

Fixed effects:
            Estimate Std. Error      df t value Pr(>|t|)  
(Intercept)   -2.856      3.941 122.528  -0.725   0.4700  
empathy       -1.642      2.528 241.000  -0.650   0.5166  
before         1.975      2.528 241.000   0.781   0.4353  
money         11.424      4.563  79.000   2.504   0.0143 *
---
Signif. codes:  0 '***' 0.001 '**' 0.01 '*' 0.05 '.' 0.1 ' ' 1

Correlation of Fixed Effects:
        (Intr) empthy before
empathy -0.321              
before  -0.321  0.000       
money   -0.686  0.000  0.000
```

In [125]:

```
class(model.ec) <- "lmerMod"
class(model.ac) <- "lmerMod"
class(model.hk) <- "lmerMod"
class(model.jp) <- "lmerMod"
class(model.all) <- "lmerMod"
stargazer(model.ec, model.ac, model.hk, model.jp, model.all, type="text", ci=TRUE, digits=2,
          star.char = c("+", "*", "**", "***"),
          star.cutoffs = c(0.1, 0.05, 0.01, 0.001),
          notes = c("+ p<0.1; * p<0.05; ** p<0.01; *** p<0.001"), 
          notes.append = F)
```

```
=====================================================================================================
                                                      Dependent variable:                            
                          ---------------------------------------------------------------------------
                                                            true_op                                  
                               (1)            (2)            (3)            (4)             (5)      
-----------------------------------------------------------------------------------------------------
popHong_Kong                                                                               5.41      
                                                                                      (-4.97, 15.79) 
                                                                                                     
popAsian_Canadian                                                                          2.64      
                                                                                      (-8.91, 14.19) 
                                                                                                     
popJapan                                                                                   1.12      
                                                                                      (-10.29, 12.53)
                                                                                                     
empathy                       7.69**         4.36+           0.56          -1.64          7.69**     
                          (1.92, 13.46)  (-0.75, 9.47)  (-3.34, 4.46)  (-6.60, 3.31)   (2.40, 12.98) 
                                                                                                     
before                        6.78*          8.07**         4.28*           1.98           6.78*     
                          (1.01, 12.55)  (2.96, 13.18)   (0.39, 8.18)  (-2.98, 6.93)   (1.49, 12.07) 
                                                                                                     
money                          3.17          -0.19           3.80          11.42*          3.17      
                          (-7.28, 13.61) (-10.25, 9.87) (-3.20, 10.81) (2.48, 20.37)  (-6.66, 12.99) 
                                                                                                     
popHong_Kong:empathy                                                                      -7.13*     
                                                                                      (-13.84, -0.42)
                                                                                                     
popAsian_Canadian:empathy                                                                  -3.33     
                                                                                      (-10.91, 4.24) 
                                                                                                     
popJapan:empathy                                                                          -9.33*     
                                                                                      (-16.46, -2.20)
                                                                                                     
popHong_Kong:before                                                                        -2.50     
                                                                                       (-9.20, 4.21) 
                                                                                                     
popAsian_Canadian:before                                                                   1.29      
                                                                                       (-6.28, 8.87) 
                                                                                                     
popJapan:before                                                                            -4.80     
                                                                                      (-11.94, 2.33) 
                                                                                                     
popHong_Kong:money                                                                         0.64      
                                                                                      (-11.71, 12.99)
                                                                                                     
popAsian_Canadian:money                                                                    -3.35     
                                                                                      (-17.26, 10.56)
                                                                                                     
popJapan:money                                                                             8.26      
                                                                                      (-4.95, 21.47) 
                                                                                                     
Constant                      -3.98          -1.34           1.44          -2.86           -3.98     
                          (-13.07, 5.12) (-9.16, 6.49)  (-4.13, 7.01)  (-10.58, 4.87) (-12.49, 4.54) 
                                                                                                     
-----------------------------------------------------------------------------------------------------
Observations                   264            252            436            324            1,276     
Log Likelihood              -1,239.73      -1,153.47      -1,996.09      -1,508.67       -5,902.11   
Akaike Inf. Crit.            2,491.47       2,318.95       4,004.18       3,029.34       11,840.22   
Bayesian Inf. Crit.          2,512.92       2,340.12       4,028.64       3,052.02       11,932.94   
=====================================================================================================
Note:                                                       + p<0.1; * p<0.05; ** p<0.01; *** p<0.001
```

## Incentives¶

In [126]:

```
dat$tokens <- (dat$money + 1) %% 2 #Use modulo arithmetic to create a before variable
table(dat$tokens, dat$money)
dat.eurocan <- dat[dat$sample=="Euro_Canadian",]
dat.asiacan <- dat[dat$sample=="Asian_Canadian",]
dat.hk <- dat[dat$sample=="Hong_Kong",]
dat.jp <- dat[dat$sample=="Japan",]
```

```
      0   1
  0   0 680
  1 596   0
```

Once again, we are running the same regression model, just changing the reference group for convenience.

### Empathy¶

In [127]:

```
#Before Empathy
model.ec.be <- lmer(true_op ~ math + after + tokens + (1 | pid), data = dat.eurocan)
model.ac.be <- lmer(true_op ~ math + after + tokens + (1 | pid), data = dat.asiacan)
model.hk.be <- lmer(true_op ~ math + after + tokens + (1 | pid), data = dat.hk)
model.jp.be <- lmer(true_op ~ math + after + tokens + (1 | pid), data = dat.jp)
model.all.be <- lmer(true_op ~ pop*(math + after + tokens) + (1 | pid), data = dat)

summary(model.ec.be)
summary(model.ac.be)
summary(model.hk.be)
summary(model.jp.be)

#After Empathy
model.ec.ae <- lmer(true_op ~ math + before + tokens + (1 | pid), data = dat.eurocan)
model.ac.ae <- lmer(true_op ~ math + before + tokens + (1 | pid), data = dat.asiacan)
model.hk.ae <- lmer(true_op ~ math + before + tokens + (1 | pid), data = dat.hk)
model.jp.ae <- lmer(true_op ~ math + before + tokens + (1 | pid), data = dat.jp)
model.all.ae <- lmer(true_op ~ pop*(math + before + tokens) + (1 | pid), data = dat)

summary(model.ec.ae)
summary(model.ac.ae)
summary(model.hk.ae)
summary(model.jp.ae)
```

```
Linear mixed model fit by REML. t-tests use Satterthwaite's method [
lmerModLmerTest]
Formula: true_op ~ math + after + tokens + (1 | pid)
   Data: dat.eurocan

REML criterion at convergence: 2479.5

Scaled residuals: 
    Min      1Q  Median      3Q     Max 
-2.5391 -0.6792  0.1237  0.6489  2.3604 

Random effects:
 Groups   Name        Variance Std.Dev.
 pid      (Intercept) 304.9    17.46   
 Residual             571.1    23.90   
Number of obs: 264, groups:  pid, 66

Fixed effects:
            Estimate Std. Error      df t value Pr(>|t|)    
(Intercept)   13.660      3.939 117.324   3.468 0.000735 ***
math          -7.689      2.942 196.000  -2.614 0.009646 ** 
after         -6.780      2.942 196.000  -2.305 0.022220 *  
tokens        -3.165      5.330  64.000  -0.594 0.554696    
---
Signif. codes:  0 '***' 0.001 '**' 0.01 '*' 0.05 '.' 0.1 ' ' 1

Correlation of Fixed Effects:
       (Intr) math   after 
math   -0.373              
after  -0.373  0.000       
tokens -0.533  0.000  0.000
```

```
Linear mixed model fit by REML. t-tests use Satterthwaite's method [
lmerModLmerTest]
Formula: true_op ~ math + after + tokens + (1 | pid)
   Data: dat.asiacan

REML criterion at convergence: 2306.9

Scaled residuals: 
     Min       1Q   Median       3Q      Max 
-2.08816 -0.70328  0.01549  0.67265  2.29343 

Random effects:
 Groups   Name        Variance Std.Dev.
 pid      (Intercept) 307.1    17.52   
 Residual             428.0    20.69   
Number of obs: 252, groups:  pid, 63

Fixed effects:
            Estimate Std. Error       df t value Pr(>|t|)   
(Intercept)  10.9060     4.1473  92.8779   2.630  0.01000 * 
math         -4.3571     2.6063 187.0000  -1.672  0.09625 . 
after        -8.0714     2.6063 187.0000  -3.097  0.00226 **
tokens        0.1871     5.1335  61.0000   0.036  0.97104   
---
Signif. codes:  0 '***' 0.001 '**' 0.01 '*' 0.05 '.' 0.1 ' ' 1

Correlation of Fixed Effects:
       (Intr) math   after 
math   -0.314              
after  -0.314  0.000       
tokens -0.648  0.000  0.000
```

```
Linear mixed model fit by REML. t-tests use Satterthwaite's method [
lmerModLmerTest]
Formula: true_op ~ math + after + tokens + (1 | pid)
   Data: dat.hk

REML criterion at convergence: 3992.2

Scaled residuals: 
     Min       1Q   Median       3Q      Max 
-2.60221 -0.58207 -0.00065  0.61574  2.61473 

Random effects:
 Groups   Name        Variance Std.Dev.
 pid      (Intercept) 239.7    15.48   
 Residual             431.3    20.77   
Number of obs: 436, groups:  pid, 109

Fixed effects:
            Estimate Std. Error       df t value Pr(>|t|)    
(Intercept)  10.0855     2.9429 174.6936   3.427 0.000761 ***
math         -0.5596     1.9892 325.0000  -0.281 0.778629    
after        -4.2844     1.9892 325.0000  -2.154 0.031986 *  
tokens       -3.8038     3.5747 107.0000  -1.064 0.289688    
---
Signif. codes:  0 '***' 0.001 '**' 0.01 '*' 0.05 '.' 0.1 ' ' 1

Correlation of Fixed Effects:
       (Intr) math   after 
math   -0.338              
after  -0.338  0.000       
tokens -0.635  0.000  0.000
```

```
Linear mixed model fit by REML. t-tests use Satterthwaite's method [
lmerModLmerTest]
Formula: true_op ~ math + after + tokens + (1 | pid)
   Data: dat.jp

REML criterion at convergence: 3017.3

Scaled residuals: 
    Min      1Q  Median      3Q     Max 
-3.1714 -0.6258  0.0113  0.6821  2.5400 

Random effects:
 Groups   Name        Variance Std.Dev.
 pid      (Intercept) 277.7    16.66   
 Residual             517.6    22.75   
Number of obs: 324, groups:  pid, 81

Fixed effects:
            Estimate Std. Error      df t value Pr(>|t|)  
(Intercept)    8.901      3.417 143.080   2.605   0.0102 *
math           1.642      2.528 241.000   0.650   0.5166  
after         -1.975      2.528 241.000  -0.781   0.4353  
tokens       -11.424      4.563  79.000  -2.504   0.0143 *
---
Signif. codes:  0 '***' 0.001 '**' 0.01 '*' 0.05 '.' 0.1 ' ' 1

Correlation of Fixed Effects:
       (Intr) math   after 
math   -0.370              
after  -0.370  0.000       
tokens -0.544  0.000  0.000
```

```
Linear mixed model fit by REML. t-tests use Satterthwaite's method [
lmerModLmerTest]
Formula: true_op ~ math + before + tokens + (1 | pid)
   Data: dat.eurocan

REML criterion at convergence: 2479.5

Scaled residuals: 
    Min      1Q  Median      3Q     Max 
-2.5391 -0.6792  0.1237  0.6489  2.3604 

Random effects:
 Groups   Name        Variance Std.Dev.
 pid      (Intercept) 304.9    17.46   
 Residual             571.1    23.90   
Number of obs: 264, groups:  pid, 66

Fixed effects:
            Estimate Std. Error      df t value Pr(>|t|)   
(Intercept)    6.880      3.939 117.324   1.746  0.08337 . 
math          -7.689      2.942 196.000  -2.614  0.00965 **
before         6.780      2.942 196.000   2.305  0.02222 * 
tokens        -3.165      5.330  64.000  -0.594  0.55470   
---
Signif. codes:  0 '***' 0.001 '**' 0.01 '*' 0.05 '.' 0.1 ' ' 1

Correlation of Fixed Effects:
       (Intr) math   before
math   -0.373              
before -0.373  0.000       
tokens -0.533  0.000  0.000
```

```
Linear mixed model fit by REML. t-tests use Satterthwaite's method [
lmerModLmerTest]
Formula: true_op ~ math + before + tokens + (1 | pid)
   Data: dat.asiacan

REML criterion at convergence: 2306.9

Scaled residuals: 
     Min       1Q   Median       3Q      Max 
-2.08816 -0.70328  0.01549  0.67265  2.29343 

Random effects:
 Groups   Name        Variance Std.Dev.
 pid      (Intercept) 307.1    17.52   
 Residual             428.0    20.69   
Number of obs: 252, groups:  pid, 63

Fixed effects:
            Estimate Std. Error       df t value Pr(>|t|)   
(Intercept)   2.8345     4.1473  92.8779   0.683  0.49602   
math         -4.3571     2.6063 187.0000  -1.672  0.09625 . 
before        8.0714     2.6063 187.0000   3.097  0.00226 **
tokens        0.1871     5.1335  61.0000   0.036  0.97104   
---
Signif. codes:  0 '***' 0.001 '**' 0.01 '*' 0.05 '.' 0.1 ' ' 1

Correlation of Fixed Effects:
       (Intr) math   before
math   -0.314              
before -0.314  0.000       
tokens -0.648  0.000  0.000
```

```
Linear mixed model fit by REML. t-tests use Satterthwaite's method [
lmerModLmerTest]
Formula: true_op ~ math + before + tokens + (1 | pid)
   Data: dat.hk

REML criterion at convergence: 3992.2

Scaled residuals: 
     Min       1Q   Median       3Q      Max 
-2.60221 -0.58207 -0.00065  0.61574  2.61473 

Random effects:
 Groups   Name        Variance Std.Dev.
 pid      (Intercept) 239.7    15.48   
 Residual             431.3    20.77   
Number of obs: 436, groups:  pid, 109

Fixed effects:
            Estimate Std. Error       df t value Pr(>|t|)  
(Intercept)   5.8011     2.9429 174.6936   1.971   0.0503 .
math         -0.5596     1.9892 325.0000  -0.281   0.7786  
before        4.2844     1.9892 325.0000   2.154   0.0320 *
tokens       -3.8038     3.5747 107.0000  -1.064   0.2897  
---
Signif. codes:  0 '***' 0.001 '**' 0.01 '*' 0.05 '.' 0.1 ' ' 1

Correlation of Fixed Effects:
       (Intr) math   before
math   -0.338              
before -0.338  0.000       
tokens -0.635  0.000  0.000
```

```
Linear mixed model fit by REML. t-tests use Satterthwaite's method [
lmerModLmerTest]
Formula: true_op ~ math + before + tokens + (1 | pid)
   Data: dat.jp

REML criterion at convergence: 3017.3

Scaled residuals: 
    Min      1Q  Median      3Q     Max 
-3.1714 -0.6258  0.0113  0.6821  2.5400 

Random effects:
 Groups   Name        Variance Std.Dev.
 pid      (Intercept) 277.7    16.66   
 Residual             517.6    22.75   
Number of obs: 324, groups:  pid, 81

Fixed effects:
            Estimate Std. Error      df t value Pr(>|t|)  
(Intercept)    6.926      3.417 143.080   2.027   0.0445 *
math           1.642      2.528 241.000   0.650   0.5166  
before         1.975      2.528 241.000   0.781   0.4353  
tokens       -11.424      4.563  79.000  -2.504   0.0143 *
---
Signif. codes:  0 '***' 0.001 '**' 0.01 '*' 0.05 '.' 0.1 ' ' 1

Correlation of Fixed Effects:
       (Intr) math   before
math   -0.370              
before -0.370  0.000       
tokens -0.544  0.000  0.000
```

In [128]:

```
#Before Empathy
class(model.ec.be) <- "lmerMod"
class(model.ac.be) <- "lmerMod"
class(model.hk.be) <- "lmerMod"
class(model.jp.be) <- "lmerMod"
class(model.all.be) <- "lmerMod"
stargazer(model.ec.be, model.ac.be, model.hk.be, model.jp.be, model.all.be,
          type="text", ci=TRUE, digits=2,
          star.char = c("+", "*", "**", "***"),
          star.cutoffs = c(0.1, 0.05, 0.01, 0.001),
          notes = c("+ p<0.1; * p<0.05; ** p<0.01; *** p<0.001"), 
          notes.append = F)

#After Empathy
class(model.ec.ae) <- "lmerMod"
class(model.ac.ae) <- "lmerMod"
class(model.hk.ae) <- "lmerMod"
class(model.jp.ae) <- "lmerMod"
class(model.all.ae) <- "lmerMod"
stargazer(model.ec.ae, model.ac.ae, model.hk.ae, model.jp.ae, model.all.ae,
          type="text", ci=TRUE, digits=2,
          star.char = c("+", "*", "**", "***"),
          star.cutoffs = c(0.1, 0.05, 0.01, 0.001),
          notes = c("+ p<0.1; * p<0.05; ** p<0.01; *** p<0.001"), 
          notes.append = F)
```

```
=======================================================================================================
                                                      Dependent variable:                              
                         ------------------------------------------------------------------------------
                                                            true_op                                    
                               (1)             (2)            (3)             (4)             (5)      
-------------------------------------------------------------------------------------------------------
popHong_Kong                                                                                 -3.57     
                                                                                        (-13.05, 5.90) 
                                                                                                       
popAsian_Canadian                                                                            -2.75     
                                                                                        (-13.59, 8.09) 
                                                                                                       
popJapan                                                                                     -4.76     
                                                                                        (-14.52, 5.00) 
                                                                                                       
math                         -7.69**         -4.36+          -0.56           1.64           -7.69**    
                         (-13.46, -1.92)  (-9.47, 0.75)  (-4.46, 3.34)   (-3.31, 6.60)  (-12.98, -2.40)
                                                                                                       
after                        -6.78*          -8.07**         -4.28*          -1.98          -6.78*     
                         (-12.55, -1.01) (-13.18, -2.96) (-8.18, -0.39)  (-6.93, 2.98)  (-12.07, -1.49)
                                                                                                       
tokens                        -3.17           0.19           -3.80          -11.42*          -3.17     
                         (-13.61, 7.28)  (-9.87, 10.25)  (-10.81, 3.20) (-20.37, -2.48) (-12.99, 6.66) 
                                                                                                       
popHong_Kong:math                                                                            7.13*     
                                                                                         (0.42, 13.84) 
                                                                                                       
popAsian_Canadian:math                                                                       3.33      
                                                                                        (-4.24, 10.91) 
                                                                                                       
popJapan:math                                                                                9.33*     
                                                                                         (2.20, 16.46) 
                                                                                                       
popHong_Kong:after                                                                           2.50      
                                                                                         (-4.21, 9.20) 
                                                                                                       
popAsian_Canadian:after                                                                      -1.29     
                                                                                         (-8.87, 6.28) 
                                                                                                       
popJapan:after                                                                               4.80      
                                                                                        (-2.33, 11.94) 
                                                                                                       
popHong_Kong:tokens                                                                          -0.64     
                                                                                        (-12.99, 11.71)
                                                                                                       
popAsian_Canadian:tokens                                                                     3.35      
                                                                                        (-10.56, 17.26)
                                                                                                       
popJapan:tokens                                                                              -8.26     
                                                                                        (-21.47, 4.95) 
                                                                                                       
Constant                    13.66***         10.91**        10.09***        8.90**         13.66***    
                          (5.94, 21.38)   (2.78, 19.03)  (4.32, 15.85)   (2.20, 15.60)   (6.44, 20.88) 
                                                                                                       
-------------------------------------------------------------------------------------------------------
Observations                   264             252            436             324            1,276     
Log Likelihood              -1,239.73       -1,153.47      -1,996.09       -1,508.67       -5,902.11   
Akaike Inf. Crit.           2,491.47        2,318.95        4,004.18       3,029.34        11,840.22   
Bayesian Inf. Crit.         2,512.92        2,340.12        4,028.64       3,052.02        11,932.94   
=======================================================================================================
Note:                                                         + p<0.1; * p<0.05; ** p<0.01; *** p<0.001

======================================================================================================
                                                      Dependent variable:                             
                         -----------------------------------------------------------------------------
                                                            true_op                                   
                               (1)            (2)            (3)             (4)             (5)      
------------------------------------------------------------------------------------------------------
popHong_Kong                                                                                -1.08     
                                                                                       (-10.56, 8.40) 
                                                                                                      
popAsian_Canadian                                                                           -4.05     
                                                                                       (-14.88, 6.79) 
                                                                                                      
popJapan                                                                                    0.05      
                                                                                        (-9.71, 9.80) 
                                                                                                      
math                         -7.69**         -4.36+         -0.56           1.64           -7.69**    
                         (-13.46, -1.92) (-9.47, 0.75)  (-4.46, 3.34)   (-3.31, 6.60)  (-12.98, -2.40)
                                                                                                      
before                        6.78*          8.07**         4.28*           1.98            6.78*     
                          (1.01, 12.55)  (2.96, 13.18)   (0.39, 8.18)   (-2.98, 6.93)   (1.49, 12.07) 
                                                                                                      
tokens                        -3.17           0.19          -3.80          -11.42*          -3.17     
                         (-13.61, 7.28)  (-9.87, 10.25) (-10.81, 3.20) (-20.37, -2.48) (-12.99, 6.66) 
                                                                                                      
popHong_Kong:math                                                                           7.13*     
                                                                                        (0.42, 13.84) 
                                                                                                      
popAsian_Canadian:math                                                                      3.33      
                                                                                       (-4.24, 10.91) 
                                                                                                      
popJapan:math                                                                               9.33*     
                                                                                        (2.20, 16.46) 
                                                                                                      
popHong_Kong:before                                                                         -2.50     
                                                                                        (-9.20, 4.21) 
                                                                                                      
popAsian_Canadian:before                                                                    1.29      
                                                                                        (-6.28, 8.87) 
                                                                                                      
popJapan:before                                                                             -4.80     
                                                                                       (-11.94, 2.33) 
                                                                                                      
popHong_Kong:tokens                                                                         -0.64     
                                                                                       (-12.99, 11.71)
                                                                                                      
popAsian_Canadian:tokens                                                                    3.35      
                                                                                       (-10.56, 17.26)
                                                                                                      
popJapan:tokens                                                                             -8.26     
                                                                                       (-21.47, 4.95) 
                                                                                                      
Constant                      6.88+           2.83          5.80*           6.93*           6.88+     
                         (-0.84, 14.60)  (-5.29, 10.96) (0.03, 11.57)   (0.23, 13.62)  (-0.34, 14.10) 
                                                                                                      
------------------------------------------------------------------------------------------------------
Observations                   264            252            436             324            1,276     
Log Likelihood              -1,239.73      -1,153.47      -1,996.09       -1,508.67       -5,902.11   
Akaike Inf. Crit.           2,491.47        2,318.95       4,004.18       3,029.34        11,840.22   
Bayesian Inf. Crit.         2,512.92        2,340.12       4,028.64       3,052.02        11,932.94   
======================================================================================================
Note:                                                        + p<0.1; * p<0.05; ** p<0.01; *** p<0.001
```

### Math¶

In [129]:

```
#Before Math
model.ec.be <- lmer(true_op ~ empathy + after + tokens + (1 | pid), data = dat.eurocan)
model.ac.be <- lmer(true_op ~ empathy + after + tokens + (1 | pid), data = dat.asiacan)
model.hk.be <- lmer(true_op ~ empathy + after + tokens + (1 | pid), data = dat.hk)
model.jp.be <- lmer(true_op ~ empathy + after + tokens + (1 | pid), data = dat.jp)
model.all.be <- lmer(true_op ~ pop*(empathy + after + tokens) + (1 | pid), data = dat)

summary(model.ec.be)
summary(model.ac.be)
summary(model.hk.be)
summary(model.jp.be)
```

```
Linear mixed model fit by REML. t-tests use Satterthwaite's method [
lmerModLmerTest]
Formula: true_op ~ empathy + after + tokens + (1 | pid)
   Data: dat.eurocan

REML criterion at convergence: 2479.5

Scaled residuals: 
    Min      1Q  Median      3Q     Max 
-2.5391 -0.6792  0.1237  0.6489  2.3604 

Random effects:
 Groups   Name        Variance Std.Dev.
 pid      (Intercept) 304.9    17.46   
 Residual             571.1    23.90   
Number of obs: 264, groups:  pid, 66

Fixed effects:
            Estimate Std. Error      df t value Pr(>|t|)   
(Intercept)    5.970      3.939 117.324   1.516  0.13232   
empathy        7.689      2.942 196.000   2.614  0.00965 **
after         -6.780      2.942 196.000  -2.305  0.02222 * 
tokens        -3.165      5.330  64.000  -0.594  0.55470   
---
Signif. codes:  0 '***' 0.001 '**' 0.01 '*' 0.05 '.' 0.1 ' ' 1

Correlation of Fixed Effects:
        (Intr) empthy after 
empathy -0.373              
after   -0.373  0.000       
tokens  -0.533  0.000  0.000
```

```
Linear mixed model fit by REML. t-tests use Satterthwaite's method [
lmerModLmerTest]
Formula: true_op ~ empathy + after + tokens + (1 | pid)
   Data: dat.asiacan

REML criterion at convergence: 2306.9

Scaled residuals: 
     Min       1Q   Median       3Q      Max 
-2.08816 -0.70328  0.01549  0.67265  2.29343 

Random effects:
 Groups   Name        Variance Std.Dev.
 pid      (Intercept) 307.1    17.52   
 Residual             428.0    20.69   
Number of obs: 252, groups:  pid, 63

Fixed effects:
            Estimate Std. Error       df t value Pr(>|t|)   
(Intercept)   6.5488     4.1473  92.8779   1.579  0.11772   
empathy       4.3571     2.6063 187.0000   1.672  0.09625 . 
after        -8.0714     2.6063 187.0000  -3.097  0.00226 **
tokens        0.1871     5.1335  61.0000   0.036  0.97104   
---
Signif. codes:  0 '***' 0.001 '**' 0.01 '*' 0.05 '.' 0.1 ' ' 1

Correlation of Fixed Effects:
        (Intr) empthy after 
empathy -0.314              
after   -0.314  0.000       
tokens  -0.648  0.000  0.000
```

```
Linear mixed model fit by REML. t-tests use Satterthwaite's method [
lmerModLmerTest]
Formula: true_op ~ empathy + after + tokens + (1 | pid)
   Data: dat.hk

REML criterion at convergence: 3992.2

Scaled residuals: 
     Min       1Q   Median       3Q      Max 
-2.60221 -0.58207 -0.00065  0.61574  2.61473 

Random effects:
 Groups   Name        Variance Std.Dev.
 pid      (Intercept) 239.7    15.48   
 Residual             431.3    20.77   
Number of obs: 436, groups:  pid, 109

Fixed effects:
            Estimate Std. Error       df t value Pr(>|t|)   
(Intercept)   9.5258     2.9429 174.6936   3.237  0.00145 **
empathy       0.5596     1.9892 325.0000   0.281  0.77863   
after        -4.2844     1.9892 325.0000  -2.154  0.03199 * 
tokens       -3.8038     3.5747 107.0000  -1.064  0.28969   
---
Signif. codes:  0 '***' 0.001 '**' 0.01 '*' 0.05 '.' 0.1 ' ' 1

Correlation of Fixed Effects:
        (Intr) empthy after 
empathy -0.338              
after   -0.338  0.000       
tokens  -0.635  0.000  0.000
```

```
Linear mixed model fit by REML. t-tests use Satterthwaite's method [
lmerModLmerTest]
Formula: true_op ~ empathy + after + tokens + (1 | pid)
   Data: dat.jp

REML criterion at convergence: 3017.3

Scaled residuals: 
    Min      1Q  Median      3Q     Max 
-3.1714 -0.6258  0.0113  0.6821  2.5400 

Random effects:
 Groups   Name        Variance Std.Dev.
 pid      (Intercept) 277.7    16.66   
 Residual             517.6    22.75   
Number of obs: 324, groups:  pid, 81

Fixed effects:
            Estimate Std. Error      df t value Pr(>|t|)   
(Intercept)   10.543      3.417 143.080   3.085  0.00244 **
empathy       -1.642      2.528 241.000  -0.650  0.51660   
after         -1.975      2.528 241.000  -0.781  0.43533   
tokens       -11.424      4.563  79.000  -2.504  0.01435 * 
---
Signif. codes:  0 '***' 0.001 '**' 0.01 '*' 0.05 '.' 0.1 ' ' 1

Correlation of Fixed Effects:
        (Intr) empthy after 
empathy -0.370              
after   -0.370  0.000       
tokens  -0.544  0.000  0.000
```

In [130]:

```
#After Math
model.ec.ae <- lmer(true_op ~ empathy + before + tokens + (1 | pid), data = dat.eurocan)
model.ac.ae <- lmer(true_op ~ empathy + before + tokens + (1 | pid), data = dat.asiacan)
model.hk.ae <- lmer(true_op ~ empathy + before + tokens + (1 | pid), data = dat.hk)
model.jp.ae <- lmer(true_op ~ empathy + before + tokens + (1 | pid), data = dat.jp)
model.all.ae <- lmer(true_op ~ pop*(empathy + before + tokens) + (1 | pid), data = dat)

summary(model.ec.ae)
summary(model.ac.ae)
summary(model.hk.ae)
summary(model.jp.ae)
```

```
Linear mixed model fit by REML. t-tests use Satterthwaite's method [
lmerModLmerTest]
Formula: true_op ~ empathy + before + tokens + (1 | pid)
   Data: dat.eurocan

REML criterion at convergence: 2479.5

Scaled residuals: 
    Min      1Q  Median      3Q     Max 
-2.5391 -0.6792  0.1237  0.6489  2.3604 

Random effects:
 Groups   Name        Variance Std.Dev.
 pid      (Intercept) 304.9    17.46   
 Residual             571.1    23.90   
Number of obs: 264, groups:  pid, 66

Fixed effects:
            Estimate Std. Error       df t value Pr(>|t|)   
(Intercept)  -0.8098     3.9394 117.3241  -0.206  0.83748   
empathy       7.6894     2.9417 196.0000   2.614  0.00965 **
before        6.7803     2.9417 196.0000   2.305  0.02222 * 
tokens       -3.1654     5.3301  64.0000  -0.594  0.55470   
---
Signif. codes:  0 '***' 0.001 '**' 0.01 '*' 0.05 '.' 0.1 ' ' 1

Correlation of Fixed Effects:
        (Intr) empthy before
empathy -0.373              
before  -0.373  0.000       
tokens  -0.533  0.000  0.000
```

```
Linear mixed model fit by REML. t-tests use Satterthwaite's method [
lmerModLmerTest]
Formula: true_op ~ empathy + before + tokens + (1 | pid)
   Data: dat.asiacan

REML criterion at convergence: 2306.9

Scaled residuals: 
     Min       1Q   Median       3Q      Max 
-2.08816 -0.70328  0.01549  0.67265  2.29343 

Random effects:
 Groups   Name        Variance Std.Dev.
 pid      (Intercept) 307.1    17.52   
 Residual             428.0    20.69   
Number of obs: 252, groups:  pid, 63

Fixed effects:
            Estimate Std. Error       df t value Pr(>|t|)   
(Intercept)  -1.5226     4.1473  92.8779  -0.367  0.71435   
empathy       4.3571     2.6063 187.0000   1.672  0.09625 . 
before        8.0714     2.6063 187.0000   3.097  0.00226 **
tokens        0.1871     5.1335  61.0000   0.036  0.97104   
---
Signif. codes:  0 '***' 0.001 '**' 0.01 '*' 0.05 '.' 0.1 ' ' 1

Correlation of Fixed Effects:
        (Intr) empthy before
empathy -0.314              
before  -0.314  0.000       
tokens  -0.648  0.000  0.000
```

```
Linear mixed model fit by REML. t-tests use Satterthwaite's method [
lmerModLmerTest]
Formula: true_op ~ empathy + before + tokens + (1 | pid)
   Data: dat.hk

REML criterion at convergence: 3992.2

Scaled residuals: 
     Min       1Q   Median       3Q      Max 
-2.60221 -0.58207 -0.00065  0.61574  2.61473 

Random effects:
 Groups   Name        Variance Std.Dev.
 pid      (Intercept) 239.7    15.48   
 Residual             431.3    20.77   
Number of obs: 436, groups:  pid, 109

Fixed effects:
            Estimate Std. Error       df t value Pr(>|t|)  
(Intercept)   5.2414     2.9429 174.6936   1.781   0.0766 .
empathy       0.5596     1.9892 325.0000   0.281   0.7786  
before        4.2844     1.9892 325.0000   2.154   0.0320 *
tokens       -3.8038     3.5747 107.0000  -1.064   0.2897  
---
Signif. codes:  0 '***' 0.001 '**' 0.01 '*' 0.05 '.' 0.1 ' ' 1

Correlation of Fixed Effects:
        (Intr) empthy before
empathy -0.338              
before  -0.338  0.000       
tokens  -0.635  0.000  0.000
```

```
Linear mixed model fit by REML. t-tests use Satterthwaite's method [
lmerModLmerTest]
Formula: true_op ~ empathy + before + tokens + (1 | pid)
   Data: dat.jp

REML criterion at convergence: 3017.3

Scaled residuals: 
    Min      1Q  Median      3Q     Max 
-3.1714 -0.6258  0.0113  0.6821  2.5400 

Random effects:
 Groups   Name        Variance Std.Dev.
 pid      (Intercept) 277.7    16.66   
 Residual             517.6    22.75   
Number of obs: 324, groups:  pid, 81

Fixed effects:
            Estimate Std. Error      df t value Pr(>|t|)  
(Intercept)    8.568      3.417 143.080   2.507   0.0133 *
empathy       -1.642      2.528 241.000  -0.650   0.5166  
before         1.975      2.528 241.000   0.781   0.4353  
tokens       -11.424      4.563  79.000  -2.504   0.0143 *
---
Signif. codes:  0 '***' 0.001 '**' 0.01 '*' 0.05 '.' 0.1 ' ' 1

Correlation of Fixed Effects:
        (Intr) empthy before
empathy -0.370              
before  -0.370  0.000       
tokens  -0.544  0.000  0.000
```

In [131]:

```
#Before Empathy
class(model.ec.be) <- "lmerMod"
class(model.ac.be) <- "lmerMod"
class(model.hk.be) <- "lmerMod"
class(model.jp.be) <- "lmerMod"
class(model.all.be) <- "lmerMod"
stargazer(model.ec.be, model.ac.be, model.hk.be, model.jp.be, model.all.be,
          type="text", ci=TRUE, digits=2,
          star.char = c("+", "*", "**", "***"),
          star.cutoffs = c(0.1, 0.05, 0.01, 0.001),
          notes = c("+ p<0.1; * p<0.05; ** p<0.01; *** p<0.001"), 
          notes.append = F)

#After Empathy
class(model.ec.ae) <- "lmerMod"
class(model.ac.ae) <- "lmerMod"
class(model.hk.ae) <- "lmerMod"
class(model.jp.ae) <- "lmerMod"
class(model.all.ae) <- "lmerMod"
stargazer(model.ec.ae, model.ac.ae, model.hk.ae, model.jp.ae, model.all.ae,
          type="text", ci=TRUE, digits=2,
          star.char = c("+", "*", "**", "***"),
          star.cutoffs = c(0.1, 0.05, 0.01, 0.001),
          notes = c("+ p<0.1; * p<0.05; ** p<0.01; *** p<0.001"), 
          notes.append = F)
```

```
========================================================================================================
                                                       Dependent variable:                              
                          ------------------------------------------------------------------------------
                                                             true_op                                    
                                (1)             (2)            (3)             (4)             (5)      
--------------------------------------------------------------------------------------------------------
popHong_Kong                                                                                  3.56      
                                                                                         (-5.92, 13.03) 
                                                                                                        
popAsian_Canadian                                                                             0.58      
                                                                                         (-10.26, 11.42)
                                                                                                        
popJapan                                                                                      4.57      
                                                                                         (-5.18, 14.33) 
                                                                                                        
empathy                       7.69**           4.36+           0.56           -1.64          7.69**     
                           (1.92, 13.46)   (-0.75, 9.47)  (-3.34, 4.46)   (-6.60, 3.31)   (2.40, 12.98) 
                                                                                                        
after                         -6.78*          -8.07**         -4.28*          -1.98          -6.78*     
                          (-12.55, -1.01) (-13.18, -2.96) (-8.18, -0.39)  (-6.93, 2.98)  (-12.07, -1.49)
                                                                                                        
tokens                         -3.17           0.19           -3.80          -11.42*          -3.17     
                          (-13.61, 7.28)  (-9.87, 10.25)  (-10.81, 3.20) (-20.37, -2.48) (-12.99, 6.66) 
                                                                                                        
popHong_Kong:empathy                                                                         -7.13*     
                                                                                         (-13.84, -0.42)
                                                                                                        
popAsian_Canadian:empathy                                                                     -3.33     
                                                                                         (-10.91, 4.24) 
                                                                                                        
popJapan:empathy                                                                             -9.33*     
                                                                                         (-16.46, -2.20)
                                                                                                        
popHong_Kong:after                                                                            2.50      
                                                                                          (-4.21, 9.20) 
                                                                                                        
popAsian_Canadian:after                                                                       -1.29     
                                                                                          (-8.87, 6.28) 
                                                                                                        
popJapan:after                                                                                4.80      
                                                                                         (-2.33, 11.94) 
                                                                                                        
popHong_Kong:tokens                                                                           -0.64     
                                                                                         (-12.99, 11.71)
                                                                                                        
popAsian_Canadian:tokens                                                                      3.35      
                                                                                         (-10.56, 17.26)
                                                                                                        
popJapan:tokens                                                                               -8.26     
                                                                                         (-21.47, 4.95) 
                                                                                                        
Constant                       5.97            6.55           9.53**         10.54**          5.97      
                          (-1.75, 13.69)  (-1.58, 14.68)  (3.76, 15.29)   (3.85, 17.24)  (-1.25, 13.19) 
                                                                                                        
--------------------------------------------------------------------------------------------------------
Observations                    264             252            436             324            1,276     
Log Likelihood               -1,239.73       -1,153.47      -1,996.09       -1,508.67       -5,902.11   
Akaike Inf. Crit.            2,491.47        2,318.95        4,004.18       3,029.34        11,840.22   
Bayesian Inf. Crit.          2,512.92        2,340.12        4,028.64       3,052.02        11,932.94   
========================================================================================================
Note:                                                          + p<0.1; * p<0.05; ** p<0.01; *** p<0.001

======================================================================================================
                                                      Dependent variable:                             
                          ----------------------------------------------------------------------------
                                                            true_op                                   
                               (1)            (2)            (3)             (4)             (5)      
------------------------------------------------------------------------------------------------------
popHong_Kong                                                                                6.05      
                                                                                       (-3.43, 15.53) 
                                                                                                      
popAsian_Canadian                                                                           -0.71     
                                                                                       (-11.55, 10.13)
                                                                                                      
popJapan                                                                                    9.38+     
                                                                                       (-0.38, 19.13) 
                                                                                                      
empathy                       7.69**         4.36+           0.56           -1.64          7.69**     
                          (1.92, 13.46)  (-0.75, 9.47)  (-3.34, 4.46)   (-6.60, 3.31)   (2.40, 12.98) 
                                                                                                      
before                        6.78*          8.07**         4.28*           1.98            6.78*     
                          (1.01, 12.55)  (2.96, 13.18)   (0.39, 8.18)   (-2.98, 6.93)   (1.49, 12.07) 
                                                                                                      
tokens                        -3.17           0.19          -3.80          -11.42*          -3.17     
                          (-13.61, 7.28) (-9.87, 10.25) (-10.81, 3.20) (-20.37, -2.48) (-12.99, 6.66) 
                                                                                                      
popHong_Kong:empathy                                                                       -7.13*     
                                                                                       (-13.84, -0.42)
                                                                                                      
popAsian_Canadian:empathy                                                                   -3.33     
                                                                                       (-10.91, 4.24) 
                                                                                                      
popJapan:empathy                                                                           -9.33*     
                                                                                       (-16.46, -2.20)
                                                                                                      
popHong_Kong:before                                                                         -2.50     
                                                                                        (-9.20, 4.21) 
                                                                                                      
popAsian_Canadian:before                                                                    1.29      
                                                                                        (-6.28, 8.87) 
                                                                                                      
popJapan:before                                                                             -4.80     
                                                                                       (-11.94, 2.33) 
                                                                                                      
popHong_Kong:tokens                                                                         -0.64     
                                                                                       (-12.99, 11.71)
                                                                                                      
popAsian_Canadian:tokens                                                                    3.35      
                                                                                       (-10.56, 17.26)
                                                                                                      
popJapan:tokens                                                                             -8.26     
                                                                                       (-21.47, 4.95) 
                                                                                                      
Constant                      -0.81          -1.52          5.24+           8.57*           -0.81     
                          (-8.53, 6.91)  (-9.65, 6.61)  (-0.53, 11.01)  (1.87, 15.26)   (-8.03, 6.41) 
                                                                                                      
------------------------------------------------------------------------------------------------------
Observations                   264            252            436             324            1,276     
Log Likelihood              -1,239.73      -1,153.47      -1,996.09       -1,508.67       -5,902.11   
Akaike Inf. Crit.            2,491.47       2,318.95       4,004.18       3,029.34        11,840.22   
Bayesian Inf. Crit.          2,512.92       2,340.12       4,028.64       3,052.02        11,932.94   
======================================================================================================
Note:                                                        + p<0.1; * p<0.05; ** p<0.01; *** p<0.001
```

In [132]:

```
save.image()
```

# Overprecision¶

In [162]:

```
dat$pop <- relevel(dat$pop, ref = "Hong_Kong")
model.ec <- lmer(zsd ~ math + after + money + (1 | pid), data = dat.eurocan)
model.ac <- lmer(zsd ~ math + after + money + (1 | pid), data = dat.asiacan)
model.hk <- lmer(zsd ~ math + after + money + (1 | pid), data = dat.hk)
model.jp <- lmer(zsd ~ math + after + money + (1 | pid), data = dat.jp)
model.all <- lmer(zsd ~ pop*(math + after + money) + (1 | pid), data = dat)
summary(model.ec)
summary(model.ac)
summary(model.hk)
summary(model.jp)
summary(model.all)
```

```
Linear mixed model fit by REML. t-tests use Satterthwaite's method [
lmerModLmerTest]
Formula: zsd ~ math + after + money + (1 | pid)
   Data: dat.eurocan

REML criterion at convergence: 618.8

Scaled residuals: 
    Min      1Q  Median      3Q     Max 
-2.5995 -0.4062 -0.0618  0.3305  6.4634 

Random effects:
 Groups   Name        Variance Std.Dev.
 pid      (Intercept) 0.3775   0.6144  
 Residual             0.4020   0.6340  
Number of obs: 264, groups:  pid, 66

Fixed effects:
             Estimate Std. Error        df t value Pr(>|t|)
(Intercept)  -0.21663    0.14638  86.18914  -1.480    0.143
math         -0.05036    0.07804 196.00000  -0.645    0.519
after        -0.01886    0.07804 196.00000  -0.242    0.809
money         0.11981    0.17416  64.00000   0.688    0.494

Correlation of Fixed Effects:
      (Intr) math   after 
math  -0.267              
after -0.267  0.000       
money -0.721  0.000  0.000
```

```
Linear mixed model fit by REML. t-tests use Satterthwaite's method [
lmerModLmerTest]
Formula: zsd ~ math + after + money + (1 | pid)
   Data: dat.asiacan

REML criterion at convergence: 553.5

Scaled residuals: 
    Min      1Q  Median      3Q     Max 
-3.9737 -0.3909  0.0156  0.3763  4.8613 

Random effects:
 Groups   Name        Variance Std.Dev.
 pid      (Intercept) 0.6655   0.8158  
 Residual             0.2858   0.5346  
Number of obs: 252, groups:  pid, 63

Fixed effects:
             Estimate Std. Error        df t value Pr(>|t|)  
(Intercept)  -0.33453    0.15685  73.77241  -2.133   0.0363 *
math         -0.17195    0.06735 187.00000  -2.553   0.0115 *
after        -0.03782    0.06735 187.00000  -0.561   0.5751  
money         0.22069    0.21656  61.00000   1.019   0.3122  
---
Signif. codes:  0 '***' 0.001 '**' 0.01 '*' 0.05 '.' 0.1 ' ' 1

Correlation of Fixed Effects:
      (Intr) math   after 
math  -0.215              
after -0.215  0.000       
money -0.657  0.000  0.000
```

```
Linear mixed model fit by REML. t-tests use Satterthwaite's method [
lmerModLmerTest]
Formula: zsd ~ math + after + money + (1 | pid)
   Data: dat.hk

REML criterion at convergence: 997.2

Scaled residuals: 
    Min      1Q  Median      3Q     Max 
-3.9825 -0.5160 -0.0456  0.4494  4.2053 

Random effects:
 Groups   Name        Variance Std.Dev.
 pid      (Intercept) 0.6431   0.8019  
 Residual             0.3274   0.5722  
Number of obs: 436, groups:  pid, 109

Fixed effects:
             Estimate Std. Error        df t value Pr(>|t|)    
(Intercept)   0.28481    0.11925 133.15431   2.388   0.0183 *  
math         -0.34924    0.05481 325.00000  -6.372 6.39e-10 ***
after        -0.12139    0.05481 325.00000  -2.215   0.0275 *  
money         0.53925    0.16328 107.00000   3.303   0.0013 ** 
---
Signif. codes:  0 '***' 0.001 '**' 0.01 '*' 0.05 '.' 0.1 ' ' 1

Correlation of Fixed Effects:
      (Intr) math   after 
math  -0.230              
after -0.230  0.000       
money -0.653  0.000  0.000
```

```
Linear mixed model fit by REML. t-tests use Satterthwaite's method [
lmerModLmerTest]
Formula: zsd ~ math + after + money + (1 | pid)
   Data: dat.jp

REML criterion at convergence: 784.6

Scaled residuals: 
    Min      1Q  Median      3Q     Max 
-3.1213 -0.3704 -0.0610  0.2590  8.3909 

Random effects:
 Groups   Name        Variance Std.Dev.
 pid      (Intercept) 0.4577   0.6765  
 Residual             0.4242   0.6513  
Number of obs: 324, groups:  pid, 81

Fixed effects:
             Estimate Std. Error        df t value Pr(>|t|)   
(Intercept)  -0.06153    0.14036 104.26946  -0.438  0.66204   
math         -0.23878    0.07237 241.00000  -3.300  0.00111 **
after        -0.04283    0.07237 241.00000  -0.592  0.55454   
money         0.32928    0.16979  79.00000   1.939  0.05603 . 
---
Signif. codes:  0 '***' 0.001 '**' 0.01 '*' 0.05 '.' 0.1 ' ' 1

Correlation of Fixed Effects:
      (Intr) math   after 
math  -0.258              
after -0.258  0.000       
money -0.717  0.000  0.000
```

```
Correlation matrix not shown by default, as p = 16 > 12.
Use print(obj, correlation=TRUE)  or
	 vcov(obj)	 if you need it
```

```
Linear mixed model fit by REML. t-tests use Satterthwaite's method [
lmerModLmerTest]
Formula: zsd ~ pop * (math + after + money) + (1 | pid)
   Data: dat

REML criterion at convergence: 2969.4

Scaled residuals: 
    Min      1Q  Median      3Q     Max 
-3.7209 -0.4220 -0.0390  0.3413  8.9783 

Random effects:
 Groups   Name        Variance Std.Dev.
 pid      (Intercept) 0.5457   0.7387  
 Residual             0.3592   0.5993  
Number of obs: 1276, groups:  pid, 319

Fixed effects:
                         Estimate Std. Error        df t value Pr(>|t|)    
(Intercept)               0.28481    0.11313 406.80028   2.518 0.012198 *  
popEuro_Canadian         -0.50144    0.19990 390.10765  -2.508 0.012533 *  
popJapan                 -0.34634    0.18513 393.00219  -1.871 0.062119 .  
popAsian_Canadian        -0.61934    0.18683 406.90501  -3.315 0.000999 ***
math                     -0.34924    0.05741 949.00000  -6.084  1.7e-09 ***
after                    -0.12139    0.05741 949.00000  -2.115 0.034719 *  
money                     0.53925    0.15288 311.00000   3.527 0.000483 ***
popEuro_Canadian:math     0.29887    0.09348 949.00000   3.197 0.001433 ** 
popJapan:math             0.11046    0.08792 949.00000   1.256 0.209293    
popAsian_Canadian:math    0.17729    0.09485 949.00000   1.869 0.061912 .  
popEuro_Canadian:after    0.10254    0.09348 949.00000   1.097 0.272948    
popJapan:after            0.07857    0.08792 949.00000   0.894 0.371754    
popAsian_Canadian:after   0.08358    0.09485 949.00000   0.881 0.378481    
popEuro_Canadian:money   -0.41944    0.25239 311.00000  -1.662 0.097552 .  
popJapan:money           -0.20996    0.23637 311.00000  -0.888 0.375073    
popAsian_Canadian:money  -0.31856    0.25261 311.00000  -1.261 0.208242    
---
Signif. codes:  0 '***' 0.001 '**' 0.01 '*' 0.05 '.' 0.1 ' ' 1
```

In [160]:

```
class(model.ec) <- "lmerMod"
class(model.ac) <- "lmerMod"
class(model.hk) <- "lmerMod"
class(model.jp) <- "lmerMod"
class(model.all) <- "lmerMod"
stargazer(model.ec, model.ac, model.hk, model.jp, model.all,
         type="text", ci=TRUE, digits=2,
          star.char = c("+", "*", "**", "***"),
          star.cutoffs = c(0.1, 0.05, 0.01, 0.001),
          notes = c("+ p<0.1; * p<0.05; ** p<0.01; *** p<0.001"), 
          notes.append = F)
```

```
=================================================================================================
                                                   Dependent variable:                           
                        -------------------------------------------------------------------------
                                                           zsd                                   
                             (1)           (2)            (3)            (4)            (5)      
-------------------------------------------------------------------------------------------------
popEuro_Canadian                                                                       -0.50*    
                                                                                   (-0.89, -0.11)
                                                                                                 
popJapan                                                                               -0.35+    
                                                                                   (-0.71, 0.02) 
                                                                                                 
popAsian_Canadian                                                                     -0.62***   
                                                                                   (-0.99, -0.25)
                                                                                                 
math                        -0.05         -0.17*        -0.35***       -0.24***       -0.35***   
                        (-0.20, 0.10) (-0.30, -0.04) (-0.46, -0.24) (-0.38, -0.10) (-0.46, -0.24)
                                                                                                 
after                       -0.02         -0.04          -0.12*         -0.04          -0.12*    
                        (-0.17, 0.13) (-0.17, 0.09)  (-0.23, -0.01) (-0.18, 0.10)  (-0.23, -0.01)
                                                                                                 
money                       0.12           0.22         0.54***         0.33+         0.54***    
                        (-0.22, 0.46) (-0.20, 0.65)   (0.22, 0.86)  (-0.004, 0.66)  (0.24, 0.84) 
                                                                                                 
popEuro_Canadian:math                                                                  0.30**    
                                                                                    (0.12, 0.48) 
                                                                                                 
popJapan:math                                                                           0.11     
                                                                                   (-0.06, 0.28) 
                                                                                                 
popAsian_Canadian:math                                                                 0.18+     
                                                                                   (-0.01, 0.36) 
                                                                                                 
popEuro_Canadian:after                                                                  0.10     
                                                                                   (-0.08, 0.29) 
                                                                                                 
popJapan:after                                                                          0.08     
                                                                                   (-0.09, 0.25) 
                                                                                                 
popAsian_Canadian:after                                                                 0.08     
                                                                                   (-0.10, 0.27) 
                                                                                                 
popEuro_Canadian:money                                                                 -0.42+    
                                                                                   (-0.91, 0.08) 
                                                                                                 
popJapan:money                                                                         -0.21     
                                                                                   (-0.67, 0.25) 
                                                                                                 
popAsian_Canadian:money                                                                -0.32     
                                                                                   (-0.81, 0.18) 
                                                                                                 
Constant                    -0.22         -0.33*         0.28*          -0.06          0.28*     
                        (-0.50, 0.07) (-0.64, -0.03)  (0.05, 0.52)  (-0.34, 0.21)   (0.06, 0.51) 
                                                                                                 
-------------------------------------------------------------------------------------------------
Observations                 264           252            436            324           1,276     
Log Likelihood             -309.40       -276.75        -498.59        -392.31       -1,484.68   
Akaike Inf. Crit.          630.80         565.49        1,009.18        796.61        3,005.36   
Bayesian Inf. Crit.        652.25         586.67        1,033.65        819.30        3,098.09   
=================================================================================================
Note:                                                   + p<0.1; * p<0.05; ** p<0.01; *** p<0.001
```

In [135]:

```
r.squaredGLMM(model.ec)
r.squaredGLMM(model.ac)
r.squaredGLMM(model.hk)
r.squaredGLMM(model.jp)
r.squaredGLMM(model.all)
```

R2m
:   0.00531612790964726

R2c
:   0.487003126824567

R2m
:   0.0205666240671963

R2c
:   0.705751911858879

R2m
:   0.0992729657033986

R2c
:   0.696106575846874

R2m
:   0.0444417552268364

R2c
:   0.540388521483339

R2m
:   0.110629873341486

R2c
:   0.646976376801489

# Reward for Accuracy¶

In [136]:

```
model.ec <- lmer(indecile2 ~ math + after + money + (1 | pid), data = dat.eurocan)
model.ac <- lmer(indecile2 ~ math + after + money + (1 | pid), data = dat.asiacan)
model.hk <- lmer(indecile2 ~ math + after + money + (1 | pid), data = dat.hk)
model.jp <- lmer(indecile2 ~ math + after + money + (1 | pid), data = dat.jp)
model.all <- lmer(indecile2 ~ pop*(math + after + money) + (1 | pid), data = dat)
```

In [137]:

```
class(model.ec) <- "lmerMod"
class(model.ac) <- "lmerMod"
class(model.hk) <- "lmerMod"
class(model.jp) <- "lmerMod"
class(model.all) <- "lmerMod"
stargazer(model.ec, model.ac, model.hk, model.jp, model.all, type="text", ci=TRUE, digits=2,
          star.char = c("+", "*", "**", "***"),
          star.cutoffs = c(0.1, 0.05, 0.01, 0.001),
          notes = c("+ p<0.1; * p<0.05; ** p<0.01; *** p<0.001"), 
          notes.append = F)
```

```
==============================================================================================
                                                 Dependent variable:                          
                        ----------------------------------------------------------------------
                                                      indecile2                               
                             (1)           (2)           (3)           (4)           (5)      
----------------------------------------------------------------------------------------------
popHong_Kong                                                                         0.43     
                                                                                (-0.25, 1.11) 
                                                                                              
popAsian_Canadian                                                                    0.37     
                                                                                (-0.39, 1.12) 
                                                                                              
popJapan                                                                             0.22     
                                                                                (-0.52, 0.96) 
                                                                                              
math                       1.32***       0.94***        0.38*        0.50**        1.32***    
                        (0.86, 1.78)  (0.45, 1.44)  (0.07, 0.69)  (0.15, 0.85)   (0.89, 1.75) 
                                                                                              
after                       0.15          0.40          0.14          0.29           0.15     
                        (-0.31, 0.61) (-0.09, 0.90) (-0.17, 0.45) (-0.06, 0.64) (-0.28, 0.58) 
                                                                                              
money                       -0.19         -0.29         -0.07         -0.05         -0.19     
                        (-0.81, 0.43) (-1.00, 0.42) (-0.49, 0.35) (-0.52, 0.42) (-0.79, 0.40) 
                                                                                              
popHong_Kong:math                                                                  -0.94***   
                                                                                (-1.48, -0.40)
                                                                                              
popAsian_Canadian:math                                                              -0.37     
                                                                                (-0.99, 0.24) 
                                                                                              
popJapan:math                                                                      -0.82**    
                                                                                (-1.39, -0.24)
                                                                                              
popHong_Kong:after                                                                  -0.01     
                                                                                (-0.56, 0.53) 
                                                                                              
popAsian_Canadian:after                                                              0.25     
                                                                                (-0.36, 0.87) 
                                                                                              
popJapan:after                                                                       0.14     
                                                                                (-0.44, 0.71) 
                                                                                              
popHong_Kong:money                                                                   0.12     
                                                                                (-0.63, 0.87) 
                                                                                              
popAsian_Canadian:money                                                             -0.10     
                                                                                (-0.94, 0.75) 
                                                                                              
popJapan:money                                                                       0.14     
                                                                                (-0.66, 0.94) 
                                                                                              
Constant                    0.67*        1.04***       1.10***       0.89***        0.67*     
                        (0.09, 1.25)  (0.44, 1.64)  (0.73, 1.46)  (0.44, 1.33)   (0.12, 1.22) 
                                                                                              
----------------------------------------------------------------------------------------------
Observations                 264           252           436           324          1,276     
Log Likelihood             -563.13       -554.22       -873.59       -640.52      -2,642.25   
Akaike Inf. Crit.         1,138.26      1,120.44      1,759.17      1,293.04       5,320.51   
Bayesian Inf. Crit.       1,159.72      1,141.62      1,783.64      1,315.72       5,413.24   
==============================================================================================
Note:                                                + p<0.1; * p<0.05; ** p<0.01; *** p<0.001
```

In [138]:

```
r.squaredGLMM(model.ec)
r.squaredGLMM(model.ac)
r.squaredGLMM(model.hk)
r.squaredGLMM(model.jp)
r.squaredGLMM(model.all)
```

R2m
:   0.0948215690357096

R2c
:   0.238978840781122

R2m
:   0.0536276102063862

R2c
:   0.253824798359922

R2m
:   0.0123479267397974

R2c
:   0.183426831645008

R2m
:   0.0263765318275981

R2c
:   0.172126820783789

R2m
:   0.0495094684488078

R2c
:   0.215708889498989

# Age and Sex¶

Here we show the run the regression models above including age and sex.

In [139]:

```
model.op <- lmer(op ~ pop+math + after + money + zage + male + (1 | pid), data = dat)
model.top <- lmer(true_op ~ pop+math + after + money + zage + male + (1 | pid), data = dat)
model.precis <- lmer(zsd ~ pop+math + after + money + zage + male + (1 | pid), data = dat)
model.reward <- lmer(indecile2 ~ pop+math + after + money + zage + male + (1 | pid), data = dat)
summary(model.op)
summary(model.top)
summary(model.precis)
summary(model.reward)
```

```
Linear mixed model fit by REML. t-tests use Satterthwaite's method [
lmerModLmerTest]
Formula: op ~ pop + math + after + money + zage + male + (1 | pid)
   Data: dat

REML criterion at convergence: 10787.8

Scaled residuals: 
     Min       1Q   Median       3Q      Max 
-2.73118 -0.56986  0.02507  0.63196  2.26157 

Random effects:
 Groups   Name        Variance Std.Dev.
 pid      (Intercept)  96.14    9.805  
 Residual             239.90   15.489  
Number of obs: 1264, groups:  pid, 316

Fixed effects:
                  Estimate Std. Error       df t value Pr(>|t|)    
(Intercept)         4.3103     1.9812 377.2894   2.176  0.03021 *  
popHong_Kong        1.7922     1.9603 309.0000   0.914  0.36130    
popAsian_Canadian   1.7204     2.2086 309.0000   0.779  0.43661    
popJapan           -0.1215     2.1352 309.0000  -0.057  0.95466    
math               -2.7089     0.8713 946.0000  -3.109  0.00193 ** 
after              -4.9430     0.8713 946.0000  -5.673 1.86e-08 ***
money               1.0706     1.4209 309.0000   0.753  0.45173    
zage                1.8276     0.7241 309.0000   2.524  0.01211 *  
male                5.7815     1.4271 309.0000   4.051 6.45e-05 ***
---
Signif. codes:  0 '***' 0.001 '**' 0.01 '*' 0.05 '.' 0.1 ' ' 1

Correlation of Fixed Effects:
            (Intr) ppHn_K ppAs_C popJpn math   after  money  zage  
popHong_Kng -0.652                                                 
popAsn_Cndn -0.578  0.554                                          
popJapan    -0.534  0.563  0.504                                   
math        -0.220  0.000  0.000  0.000                            
after       -0.220  0.000  0.000  0.000  0.000                     
money       -0.419  0.090  0.083  0.011  0.000  0.000              
zage        -0.054 -0.005  0.010  0.185  0.000  0.000 -0.009       
male        -0.331  0.012  0.002 -0.113  0.000  0.000 -0.043  0.018
```

```
Linear mixed model fit by REML. t-tests use Satterthwaite's method [
lmerModLmerTest]
Formula: true_op ~ pop + math + after + money + zage + male + (1 | pid)
   Data: dat

REML criterion at convergence: 11739.2

Scaled residuals: 
     Min       1Q   Median       3Q      Max 
-3.07081 -0.63022  0.02843  0.64065  2.63490 

Random effects:
 Groups   Name        Variance Std.Dev.
 pid      (Intercept) 270.5    16.45   
 Residual             485.5    22.03   
Number of obs: 1264, groups:  pid, 316

Fixed effects:
                  Estimate Std. Error       df t value Pr(>|t|)    
(Intercept)         6.0381     3.1095 363.7578   1.942   0.0529 .  
popHong_Kong        0.9657     3.1059 309.0000   0.311   0.7561    
popAsian_Canadian   0.2638     3.4993 309.0000   0.075   0.9400    
popJapan           -0.5182     3.3831 309.0000  -0.153   0.8784    
math               -2.2658     1.2396 946.0000  -1.828   0.0679 .  
after              -4.9430     1.2396 946.0000  -3.988 7.18e-05 ***
money               4.5801     2.2513 309.0000   2.034   0.0428 *  
zage                1.8496     1.1473 309.0000   1.612   0.1079    
male               -0.6009     2.2611 309.0000  -0.266   0.7906    
---
Signif. codes:  0 '***' 0.001 '**' 0.01 '*' 0.05 '.' 0.1 ' ' 1

Correlation of Fixed Effects:
            (Intr) ppHn_K ppAs_C popJpn math   after  money  zage  
popHong_Kng -0.658                                                 
popAsn_Cndn -0.584  0.554                                          
popJapan    -0.539  0.563  0.504                                   
math        -0.199  0.000  0.000  0.000                            
after       -0.199  0.000  0.000  0.000  0.000                     
money       -0.423  0.090  0.083  0.011  0.000  0.000              
zage        -0.054 -0.005  0.010  0.185  0.000  0.000 -0.009       
male        -0.335  0.012  0.002 -0.113  0.000  0.000 -0.043  0.018
```

```
Linear mixed model fit by REML. t-tests use Satterthwaite's method [
lmerModLmerTest]
Formula: zsd ~ pop + math + after + money + zage + male + (1 | pid)
   Data: dat

REML criterion at convergence: 2928.9

Scaled residuals: 
    Min      1Q  Median      3Q     Max 
-3.7713 -0.4374 -0.0298  0.3499  8.9723 

Random effects:
 Groups   Name        Variance Std.Dev.
 pid      (Intercept) 0.5209   0.7217  
 Residual             0.3617   0.6015  
Number of obs: 1264, groups:  pid, 316

Fixed effects:
                   Estimate Std. Error        df t value Pr(>|t|)    
(Intercept)        -0.09509    0.12024 334.81600  -0.791 0.429578    
popHong_Kong        0.51702    0.12267 309.00001   4.215 3.29e-05 ***
popAsian_Canadian  -0.11481    0.13821 309.00000  -0.831 0.406785    
popJapan            0.17863    0.13362 309.00000   1.337 0.182237    
math               -0.22822    0.03383 946.00000  -6.745 2.66e-11 ***
after              -0.06002    0.03383 946.00000  -1.774 0.076374 .  
money               0.36060    0.08892 309.00000   4.056 6.34e-05 ***
zage               -0.10005    0.04531 309.00000  -2.208 0.027983 *  
male               -0.29681    0.08930 309.00000  -3.324 0.000995 ***
---
Signif. codes:  0 '***' 0.001 '**' 0.01 '*' 0.05 '.' 0.1 ' ' 1

Correlation of Fixed Effects:
            (Intr) ppHn_K ppAs_C popJpn math   after  money  zage  
popHong_Kng -0.672                                                 
popAsn_Cndn -0.596  0.554                                          
popJapan    -0.550  0.563  0.504                                   
math        -0.141  0.000  0.000  0.000                            
after       -0.141  0.000  0.000  0.000  0.000                     
money       -0.432  0.090  0.083  0.011  0.000  0.000              
zage        -0.055 -0.005  0.010  0.185  0.000  0.000 -0.009       
male        -0.342  0.012  0.002 -0.113  0.000  0.000 -0.043  0.018
```

```
Linear mixed model fit by REML. t-tests use Satterthwaite's method [
lmerModLmerTest]
Formula: indecile2 ~ pop + math + after + money + zage + male + (1 | pid)
   Data: dat

REML criterion at convergence: 5252.2

Scaled residuals: 
    Min      1Q  Median      3Q     Max 
-1.5070 -0.6217 -0.2711  0.4276  4.4534 

Random effects:
 Groups   Name        Variance Std.Dev.
 pid      (Intercept) 0.6379   0.7987  
 Residual             3.1908   1.7863  
Number of obs: 1264, groups:  pid, 316

Fixed effects:
                   Estimate Std. Error        df t value Pr(>|t|)    
(Intercept)         0.75547    0.19405 408.90180   3.893 0.000115 ***
popHong_Kong        0.02859    0.18798 309.00000   0.152 0.879225    
popAsian_Canadian   0.27274    0.21179 309.00000   1.288 0.198796    
popJapan           -0.05545    0.20476 309.00000  -0.271 0.786711    
math                0.72943    0.10049 946.00000   7.259 8.13e-13 ***
after               0.23259    0.10049 946.00000   2.315 0.020843 *  
money              -0.14223    0.13626 309.00000  -1.044 0.297372    
zage                0.03940    0.06944 309.00000   0.567 0.570858    
male                0.27237    0.13685 309.00000   1.990 0.047439 *  
---
Signif. codes:  0 '***' 0.001 '**' 0.01 '*' 0.05 '.' 0.1 ' ' 1

Correlation of Fixed Effects:
            (Intr) ppHn_K ppAs_C popJpn math   after  money  zage  
popHong_Kng -0.638                                                 
popAsn_Cndn -0.566  0.554                                          
popJapan    -0.523  0.563  0.504                                   
math        -0.259  0.000  0.000  0.000                            
after       -0.259  0.000  0.000  0.000  0.000                     
money       -0.410  0.090  0.083  0.011  0.000  0.000              
zage        -0.053 -0.005  0.010  0.185  0.000  0.000 -0.009       
male        -0.325  0.012  0.002 -0.113  0.000  0.000 -0.043  0.018
```

In [140]:

```
r.squaredGLMM(model.op)
r.squaredGLMM(model.top)
r.squaredGLMM(model.precis)
r.squaredGLMM(model.reward)
```

R2m
:   0.0564845598110949

R2c
:   0.326424791779724

R2m
:   0.0214566198875316

R2c
:   0.371582095838211

R2m
:   0.131086853934811

R2c
:   0.643880529113762

R2m
:   0.0456728710548555

R2c
:   0.204668383913063

In [141]:

```
class(model.op) <- "lmerMod"
class(model.top) <- "lmerMod"
class(model.precis) <- "lmerMod"
class(model.reward) <- "lmerMod"
stargazer(model.op, model.top, model.precis, model.reward, type="text", ci=TRUE, digits=2,
          star.char = c("+", "*", "**", "***"),
          star.cutoffs = c(0.1, 0.05, 0.01, 0.001),
          notes = c("+ p<0.1; * p<0.05; ** p<0.01; *** p<0.001"), 
          notes.append = F)
```

```
==============================================================================
                                       Dependent variable:                    
                    ----------------------------------------------------------
                          op          true_op          zsd         indecile2  
                         (1)            (2)            (3)            (4)     
------------------------------------------------------------------------------
popHong_Kong             1.79           0.97         0.52***         0.03     
                    (-2.05, 5.63)  (-5.12, 7.05)   (0.28, 0.76)  (-0.34, 0.40)
                                                                              
popAsian_Canadian        1.72           0.26          -0.11          0.27     
                    (-2.61, 6.05)  (-6.59, 7.12)  (-0.39, 0.16)  (-0.14, 0.69)
                                                                              
popJapan                -0.12          -0.52           0.18          -0.06    
                    (-4.31, 4.06)  (-7.15, 6.11)  (-0.08, 0.44)  (-0.46, 0.35)
                                                                              
math                   -2.71**         -2.27+        -0.23***       0.73***   
                    (-4.42, -1.00) (-4.70, 0.16)  (-0.29, -0.16) (0.53, 0.93) 
                                                                              
after                  -4.94***       -4.94***        -0.06+         0.23*    
                    (-6.65, -3.24) (-7.37, -2.51) (-0.13, 0.01)  (0.04, 0.43) 
                                                                              
money                    1.07          4.58*         0.36***         -0.14    
                    (-1.71, 3.86)   (0.17, 8.99)   (0.19, 0.53)  (-0.41, 0.12)
                                                                              
zage                    1.83*           1.85          -0.10*         0.04     
                     (0.41, 3.25)  (-0.40, 4.10)  (-0.19, -0.01) (-0.10, 0.18)
                                                                              
male                   5.78***         -0.60         -0.30***        0.27*    
                     (2.98, 8.58)  (-5.03, 3.83)  (-0.47, -0.12) (0.004, 0.54)
                                                                              
Constant                4.31*          6.04+          -0.10         0.76***   
                     (0.43, 8.19)  (-0.06, 12.13) (-0.33, 0.14)  (0.38, 1.14) 
                                                                              
------------------------------------------------------------------------------
Observations            1,264          1,264          1,264          1,264    
Log Likelihood        -5,393.91      -5,869.58      -1,464.46      -2,626.12  
Akaike Inf. Crit.     10,809.81      11,761.16       2,950.91      5,274.24   
Bayesian Inf. Crit.   10,866.37      11,817.73       3,007.48      5,330.80   
==============================================================================
Note:                                + p<0.1; * p<0.05; ** p<0.01; *** p<0.001
```

In [142]:

```
model.op <- lmer(decileoc ~ pop*(math + after + money + zage + male) + (1 | pid), data = dat)
model.top <- lmer(decileoc2 ~ pop*(math + after + money + zage + male) + (1 | pid), data = dat)
model.precis <- lmer(zsd ~ pop*(math + after + money + zage + male) + (1 | pid), data = dat)
model.reward <- lmer(indecile2 ~ pop*(math + after + money + zage + male) + (1 | pid), data = dat)
summary(model.op)
summary(model.top)
summary(model.precis)
summary(model.reward)
```

```
Correlation matrix not shown by default, as p = 24 > 12.
Use print(obj, correlation=TRUE)  or
	 vcov(obj)	 if you need it
```

```
Linear mixed model fit by REML. t-tests use Satterthwaite's method [
lmerModLmerTest]
Formula: decileoc ~ pop * (math + after + money + zage + male) + (1 |      pid)
   Data: dat

REML criterion at convergence: 281.3

Scaled residuals: 
    Min      1Q  Median      3Q     Max 
-3.2207 -0.6300  0.0411  0.6812  2.5147 

Random effects:
 Groups   Name        Variance Std.Dev.
 pid      (Intercept) 0.03058  0.1749  
 Residual             0.04964  0.2228  
Number of obs: 1264, groups:  pid, 316

Fixed effects:
                          Estimate Std. Error         df t value Pr(>|t|)   
(Intercept)               0.151732   0.050034 411.335196   3.033  0.00258 **
popHong_Kong             -0.090483   0.062493 415.813943  -1.448  0.14840   
popAsian_Canadian         0.006429   0.068668 423.384701   0.094  0.92545   
popJapan                 -0.080887   0.078905 377.600677  -1.025  0.30596   
math                     -0.087500   0.027425 939.999997  -3.191  0.00147 **
after                    -0.067803   0.027425 939.999997  -2.472  0.01360 * 
money                     0.001408   0.053146 300.000008   0.026  0.97888   
zage                      0.048296   0.024945 300.000007   1.936  0.05379 . 
male                      0.011686   0.051720 300.000006   0.226  0.82140   
popHong_Kong:math         0.046157   0.034810 939.999997   1.326  0.18517   
popAsian_Canadian:math    0.029643   0.039243 939.999997   0.755  0.45022   
popJapan:math             0.084019   0.037154 939.999997   2.261  0.02397 * 
popHong_Kong:after        0.024423   0.034810 939.999997   0.702  0.48309   
popAsian_Canadian:after  -0.012911   0.039243 939.999997  -0.329  0.74222   
popJapan:after            0.050398   0.037154 939.999997   1.356  0.17528   
popHong_Kong:money        0.051683   0.066586 300.000007   0.776  0.43825   
popAsian_Canadian:money   0.017817   0.075358 300.000006   0.236  0.81326   
popJapan:money            0.095671   0.071674 300.000006   1.335  0.18295   
popHong_Kong:zage        -0.059294   0.038216 300.000006  -1.552  0.12182   
popAsian_Canadian:zage   -0.042478   0.030025 300.000006  -1.415  0.15818   
popJapan:zage             0.008978   0.070270 300.000005   0.128  0.89842   
popHong_Kong:male         0.063078   0.066245 300.000006   0.952  0.34176   
popAsian_Canadian:male   -0.056605   0.074657 300.000005  -0.758  0.44893   
popJapan:male            -0.044787   0.071515 300.000005  -0.626  0.53162   
---
Signif. codes:  0 '***' 0.001 '**' 0.01 '*' 0.05 '.' 0.1 ' ' 1
```

```
Correlation matrix not shown by default, as p = 24 > 12.
Use print(obj, correlation=TRUE)  or
	 vcov(obj)	 if you need it
```

```
Linear mixed model fit by REML. t-tests use Satterthwaite's method [
lmerModLmerTest]
Formula: decileoc2 ~ pop * (math + after + money + zage + male) + (1 |  
    pid)
   Data: dat

REML criterion at convergence: 227.6

Scaled residuals: 
    Min      1Q  Median      3Q     Max 
-3.2779 -0.6333  0.0411  0.6631  2.5980 

Random effects:
 Groups   Name        Variance Std.Dev.
 pid      (Intercept) 0.02713  0.1647  
 Residual             0.04829  0.2197  
Number of obs: 1264, groups:  pid, 316

Fixed effects:
                          Estimate Std. Error         df t value Pr(>|t|)   
(Intercept)               0.104137   0.048017 419.058262   2.169  0.03066 * 
popHong_Kong             -0.063461   0.059983 423.851580  -1.058  0.29067   
popAsian_Canadian         0.025190   0.065928 431.953399   0.382  0.70258   
popJapan                 -0.079396   0.075625 382.951536  -1.050  0.29444   
math                     -0.076894   0.027048 940.000000  -2.843  0.00457 **
after                    -0.067803   0.027048 940.000000  -2.507  0.01235 * 
money                     0.014976   0.050749 299.999997   0.295  0.76812   
zage                      0.051851   0.023820 300.000000   2.177  0.03028 * 
male                      0.007485   0.049388 299.999998   0.152  0.87963   
popHong_Kong:math         0.070737   0.034332 940.000000   2.060  0.03964 * 
popAsian_Canadian:math    0.033323   0.038704 940.000000   0.861  0.38949   
popJapan:math             0.093666   0.036644 940.000000   2.556  0.01074 * 
popHong_Kong:after        0.024423   0.034332 940.000000   0.711  0.47702   
popAsian_Canadian:after  -0.012911   0.038704 940.000000  -0.334  0.73877   
popJapan:after            0.050398   0.036644 940.000000   1.375  0.16936   
popHong_Kong:money        0.028847   0.063583 299.999998   0.454  0.65038   
popAsian_Canadian:money  -0.005643   0.071959 299.999998  -0.078  0.93754   
popJapan:money            0.086436   0.068442 299.999998   1.263  0.20760   
popHong_Kong:zage        -0.065129   0.036492 300.000000  -1.785  0.07532 . 
popAsian_Canadian:zage   -0.046467   0.028671 300.000000  -1.621  0.10613   
popJapan:zage             0.009007   0.067101 300.000000   0.134  0.89331   
popHong_Kong:male         0.038106   0.063257 299.999999   0.602  0.54736   
popAsian_Canadian:male   -0.058529   0.071290 299.999999  -0.821  0.41230   
popJapan:male            -0.045299   0.068290 299.999999  -0.663  0.50762   
---
Signif. codes:  0 '***' 0.001 '**' 0.01 '*' 0.05 '.' 0.1 ' ' 1
```

```
Correlation matrix not shown by default, as p = 24 > 12.
Use print(obj, correlation=TRUE)  or
	 vcov(obj)	 if you need it
```

```
Linear mixed model fit by REML. t-tests use Satterthwaite's method [
lmerModLmerTest]
Formula: zsd ~ pop * (math + after + money + zage + male) + (1 | pid)
   Data: dat

REML criterion at convergence: 2935.4

Scaled residuals: 
    Min      1Q  Median      3Q     Max 
-3.7815 -0.4309 -0.0443  0.3531  9.0330 

Random effects:
 Groups   Name        Variance Std.Dev.
 pid      (Intercept) 0.5151   0.7177  
 Residual             0.3594   0.5995  
Number of obs: 1264, groups:  pid, 316

Fixed effects:
                          Estimate Std. Error         df t value Pr(>|t|)    
(Intercept)              -0.261420   0.180716 356.115502  -1.447 0.148895    
popHong_Kong              0.756406   0.225426 358.345916   3.355 0.000877 ***
popAsian_Canadian         0.154990   0.247169 362.115017   0.627 0.531016    
popJapan                  0.262185   0.287917 339.282159   0.911 0.363139    
math                     -0.050365   0.073795 940.000000  -0.682 0.495091    
after                    -0.018856   0.073795 940.000000  -0.256 0.798378    
money                     0.160988   0.199364 300.000005   0.808 0.420015    
zage                     -0.163484   0.093574 300.000001  -1.747 0.081643 .  
male                      0.088275   0.194016 300.000004   0.455 0.649448    
popHong_Kong:math        -0.296186   0.093667 940.000000  -3.162 0.001617 ** 
popAsian_Canadian:math   -0.121581   0.105597 940.000000  -1.151 0.249871    
popJapan:math            -0.209557   0.099976 940.000000  -2.096 0.036342 *  
popHong_Kong:after       -0.094440   0.093667 940.000000  -1.008 0.313594    
popAsian_Canadian:after  -0.018962   0.105597 940.000000  -0.180 0.857529    
popJapan:after           -0.020445   0.099976 940.000000  -0.204 0.838011    
popHong_Kong:money        0.373959   0.249778 300.000003   1.497 0.135402    
popAsian_Canadian:money   0.131892   0.282685 300.000002   0.467 0.641147    
popJapan:money            0.191080   0.268867 300.000003   0.711 0.477832    
popHong_Kong:zage        -0.002849   0.143356 300.000000  -0.020 0.984155    
popAsian_Canadian:zage    0.115743   0.112631 300.000001   1.028 0.304952    
popJapan:zage             0.083460   0.263599 300.000000   0.317 0.751753    
popHong_Kong:male        -0.517019   0.248500 300.000002  -2.081 0.038323 *  
popAsian_Canadian:male   -0.628783   0.280057 300.000002  -2.245 0.025484 *  
popJapan:male            -0.230765   0.268272 300.000002  -0.860 0.390369    
---
Signif. codes:  0 '***' 0.001 '**' 0.01 '*' 0.05 '.' 0.1 ' ' 1
```

```
Correlation matrix not shown by default, as p = 24 > 12.
Use print(obj, correlation=TRUE)  or
	 vcov(obj)	 if you need it
```

```
Linear mixed model fit by REML. t-tests use Satterthwaite's method [
lmerModLmerTest]
Formula: indecile2 ~ pop * (math + after + money + zage + male) + (1 |  
    pid)
   Data: dat

REML criterion at convergence: 5236.1

Scaled residuals: 
    Min      1Q  Median      3Q     Max 
-1.7880 -0.6245 -0.2705  0.4268  4.2968 

Random effects:
 Groups   Name        Variance Std.Dev.
 pid      (Intercept) 0.6302   0.7939  
 Residual             3.1634   1.7786  
Number of obs: 1264, groups:  pid, 316

Fixed effects:
                         Estimate Std. Error        df t value Pr(>|t|)    
(Intercept)               0.48246    0.30707 520.03398   1.571 0.116745    
popHong_Kong              0.60699    0.38430 528.83581   1.579 0.114823    
popAsian_Canadian         0.32762    0.42366 543.66109   0.773 0.439680    
popJapan                  0.16643    0.47643 453.19598   0.349 0.727003    
math                      1.31818    0.21893 940.00000   6.021 2.48e-09 ***
after                     0.15152    0.21893 940.00000   0.692 0.489063    
money                    -0.34719    0.30557 300.00000  -1.136 0.256773    
zage                      0.34887    0.14342 300.00000   2.432 0.015581 *  
male                      0.47843    0.29737 300.00000   1.609 0.108698    
popHong_Kong:math        -0.92929    0.27789 940.00000  -3.344 0.000858 ***
popAsian_Canadian:math   -0.37374    0.31328 940.00000  -1.193 0.233174    
popJapan:math            -0.78654    0.29660 940.00000  -2.652 0.008141 ** 
popHong_Kong:after       -0.01263    0.27789 940.00000  -0.045 0.963769    
popAsian_Canadian:after   0.25325    0.31328 940.00000   0.808 0.419078    
popJapan:after            0.13962    0.29660 940.00000   0.471 0.637933    
popHong_Kong:money        0.24978    0.38284 300.00000   0.652 0.514619    
popAsian_Canadian:money  -0.09579    0.43327 300.00000  -0.221 0.825181    
popJapan:money            0.35026    0.41209 300.00000   0.850 0.396028    
popHong_Kong:zage        -0.16492    0.21972 300.00000  -0.751 0.453496    
popAsian_Canadian:zage   -0.44518    0.17263 300.00000  -2.579 0.010391 *  
popJapan:zage            -0.56055    0.40402 300.00000  -1.387 0.166338    
popHong_Kong:male        -0.49099    0.38088 300.00000  -1.289 0.198353    
popAsian_Canadian:male    0.17663    0.42925 300.00000   0.411 0.681000    
popJapan:male            -0.31081    0.41118 300.00000  -0.756 0.450308    
---
Signif. codes:  0 '***' 0.001 '**' 0.01 '*' 0.05 '.' 0.1 ' ' 1
```

In [143]:

```
r.squaredGLMM(model.op)
r.squaredGLMM(model.top)
r.squaredGLMM(model.precis)
r.squaredGLMM(model.reward)
```

R2m
:   0.0423434943043876

R2c
:   0.40739817097892

R2m
:   0.0406619755666121

R2c
:   0.385751009494721

R2m
:   0.154766821446079

R2c
:   0.652608969575207

R2m
:   0.0672486116605385

R2c
:   0.222205433885324

In [144]:

```
class(model.op) <- "lmerMod"
class(model.top) <- "lmerMod"
class(model.precis) <- "lmerMod"
class(model.reward) <- "lmerMod"
stargazer(model.op, model.top, model.precis, model.reward, type="text", ci=TRUE, digits=2,
          star.char = c("+", "*", "**", "***"),
          star.cutoffs = c(0.1, 0.05, 0.01, 0.001),
          notes = c("+ p<0.1; * p<0.05; ** p<0.01; *** p<0.001"), 
          notes.append = F)
```

```
===================================================================================
                                            Dependent variable:                    
                        -----------------------------------------------------------
                           decileoc      decileoc2         zsd         indecile2   
                             (1)            (2)            (3)            (4)      
-----------------------------------------------------------------------------------
popHong_Kong                -0.09          -0.06         0.76***          0.61     
                        (-0.21, 0.03)  (-0.18, 0.05)   (0.31, 1.20)  (-0.15, 1.36) 
                                                                                   
popAsian_Canadian            0.01           0.03           0.15           0.33     
                        (-0.13, 0.14)  (-0.10, 0.15)  (-0.33, 0.64)  (-0.50, 1.16) 
                                                                                   
popJapan                    -0.08          -0.08           0.26           0.17     
                        (-0.24, 0.07)  (-0.23, 0.07)  (-0.30, 0.83)  (-0.77, 1.10) 
                                                                                   
math                       -0.09**        -0.08**         -0.05         1.32***    
                        (-0.14, -0.03) (-0.13, -0.02) (-0.19, 0.09)   (0.89, 1.75) 
                                                                                   
after                       -0.07*         -0.07*         -0.02           0.15     
                        (-0.12, -0.01) (-0.12, -0.01) (-0.16, 0.13)  (-0.28, 0.58) 
                                                                                   
money                       0.001           0.01           0.16          -0.35     
                        (-0.10, 0.11)  (-0.08, 0.11)  (-0.23, 0.55)  (-0.95, 0.25) 
                                                                                   
zage                        0.05+          0.05*          -0.16+         0.35*     
                        (-0.001, 0.10)  (0.01, 0.10)  (-0.35, 0.02)   (0.07, 0.63) 
                                                                                   
male                         0.01           0.01           0.09           0.48     
                        (-0.09, 0.11)  (-0.09, 0.10)  (-0.29, 0.47)  (-0.10, 1.06) 
                                                                                   
popHong_Kong:math            0.05          0.07*         -0.30**        -0.93***   
                        (-0.02, 0.11)  (0.003, 0.14)  (-0.48, -0.11) (-1.47, -0.38)
                                                                                   
popAsian_Canadian:math       0.03           0.03          -0.12          -0.37     
                        (-0.05, 0.11)  (-0.04, 0.11)  (-0.33, 0.09)  (-0.99, 0.24) 
                                                                                   
popJapan:math               0.08*          0.09*          -0.21*        -0.79**    
                         (0.01, 0.16)   (0.02, 0.17)  (-0.41, -0.01) (-1.37, -0.21)
                                                                                   
popHong_Kong:after           0.02           0.02          -0.09          -0.01     
                        (-0.04, 0.09)  (-0.04, 0.09)  (-0.28, 0.09)  (-0.56, 0.53) 
                                                                                   
popAsian_Canadian:after     -0.01          -0.01          -0.02           0.25     
                        (-0.09, 0.06)  (-0.09, 0.06)  (-0.23, 0.19)  (-0.36, 0.87) 
                                                                                   
popJapan:after               0.05           0.05          -0.02           0.14     
                        (-0.02, 0.12)  (-0.02, 0.12)  (-0.22, 0.18)  (-0.44, 0.72) 
                                                                                   
popHong_Kong:money           0.05           0.03           0.37           0.25     
                        (-0.08, 0.18)  (-0.10, 0.15)  (-0.12, 0.86)  (-0.50, 1.00) 
                                                                                   
popAsian_Canadian:money      0.02          -0.01           0.13          -0.10     
                        (-0.13, 0.17)  (-0.15, 0.14)  (-0.42, 0.69)  (-0.94, 0.75) 
                                                                                   
popJapan:money               0.10           0.09           0.19           0.35     
                        (-0.04, 0.24)  (-0.05, 0.22)  (-0.34, 0.72)  (-0.46, 1.16) 
                                                                                   
popHong_Kong:zage           -0.06          -0.07+         -0.003         -0.16     
                        (-0.13, 0.02)  (-0.14, 0.01)  (-0.28, 0.28)  (-0.60, 0.27) 
                                                                                   
popAsian_Canadian:zage      -0.04          -0.05           0.12         -0.45**    
                        (-0.10, 0.02)  (-0.10, 0.01)  (-0.11, 0.34)  (-0.78, -0.11)
                                                                                   
popJapan:zage                0.01           0.01           0.08          -0.56     
                        (-0.13, 0.15)  (-0.12, 0.14)  (-0.43, 0.60)  (-1.35, 0.23) 
                                                                                   
popHong_Kong:male            0.06           0.04          -0.52*         -0.49     
                        (-0.07, 0.19)  (-0.09, 0.16)  (-1.00, -0.03) (-1.24, 0.26) 
                                                                                   
popAsian_Canadian:male      -0.06          -0.06          -0.63*          0.18     
                        (-0.20, 0.09)  (-0.20, 0.08)  (-1.18, -0.08) (-0.66, 1.02) 
                                                                                   
popJapan:male               -0.04          -0.05          -0.23          -0.31     
                        (-0.18, 0.10)  (-0.18, 0.09)  (-0.76, 0.30)  (-1.12, 0.50) 
                                                                                   
Constant                    0.15**         0.10*          -0.26           0.48     
                         (0.05, 0.25)   (0.01, 0.20)  (-0.62, 0.09)  (-0.12, 1.08) 
                                                                                   
-----------------------------------------------------------------------------------
Observations                1,264          1,264          1,264          1,264     
Log Likelihood             -140.64        -113.80       -1,467.72      -2,618.05   
Akaike Inf. Crit.           333.28         279.60        2,987.44       5,288.10   
Bayesian Inf. Crit.         466.97         413.30        3,121.14       5,421.79   
===================================================================================
Note:                                     + p<0.1; * p<0.05; ** p<0.01; *** p<0.001
```

In [145]:

```
save.image()
```

# Order checks¶

In [146]:

```
dat$math_first <- ifelse(dat$condition %in% c(0,2),0,1)
table(dat$condition, dat$math_first)
dat.eurocan <- dat[dat$sample=="Euro_Canadian",]
dat.asiacan <- dat[dat$sample=="Asian_Canadian",]
dat.hk <- dat[dat$sample=="Hong_Kong",]
dat.jp <- dat[dat$sample=="Japan",]
model.ec <- lmer(op ~ math + after + money + math_first + (1 | pid), data = dat.eurocan)
model.ac <- lmer(op ~ math + after + money + math_first + (1 | pid), data = dat.asiacan)
model.hk <- lmer(op ~ math + after + money + math_first + (1 | pid), data = dat.hk)
model.jp <- lmer(op ~ math + after + money + math_first + (1 | pid), data = dat.jp)
summary(model.ec)
summary(model.ac)
summary(model.hk)
summary(model.jp)

model.ec <- lmer(true_op ~ math + after + money + math_first + (1 | pid), data = dat.eurocan)
model.ac <- lmer(true_op ~ math + after + money + math_first + (1 | pid), data = dat.asiacan)
model.hk <- lmer(true_op ~ math + after + money + math_first + (1 | pid), data = dat.hk)
model.jp <- lmer(true_op ~ math + after + money + math_first + (1 | pid), data = dat.jp)
summary(model.ec)
summary(model.ac)
summary(model.hk)
summary(model.jp)

model.ec <- lmer(zsd ~ math + after + money + math_first + (1 | pid), data = dat.eurocan)
model.ac <- lmer(zsd ~ math + after + money + math_first + (1 | pid), data = dat.asiacan)
model.hk <- lmer(zsd ~ math + after + money + math_first + (1 | pid), data = dat.hk)
model.jp <- lmer(zsd ~ math + after + money + math_first + (1 | pid), data = dat.jp)
summary(model.ec)
summary(model.ac)
summary(model.hk)
summary(model.jp)
```

```
      0   1
  0 336   0
  1   0 260
  2 372   0
  3   0 308
```

```
Linear mixed model fit by REML. t-tests use Satterthwaite's method [
lmerModLmerTest]
Formula: op ~ math + after + money + math_first + (1 | pid)
   Data: dat.eurocan

REML criterion at convergence: 2245.5

Scaled residuals: 
     Min       1Q   Median       3Q      Max 
-2.83501 -0.52582  0.00418  0.64305  2.30298 

Random effects:
 Groups   Name        Variance Std.Dev.
 pid      (Intercept)  59.47    7.711  
 Residual             268.15   16.375  
Number of obs: 264, groups:  pid, 66

Fixed effects:
            Estimate Std. Error      df t value Pr(>|t|)    
(Intercept)   10.618      2.879 106.909   3.688 0.000358 ***
math          -9.356      2.016 196.000  -4.642 6.32e-06 ***
after         -6.780      2.016 196.000  -3.364 0.000925 ***
money          1.825      2.834  63.000   0.644 0.522044    
math_first     1.222      2.790  63.000   0.438 0.662835    
---
Signif. codes:  0 '***' 0.001 '**' 0.01 '*' 0.05 '.' 0.1 ' ' 1

Correlation of Fixed Effects:
           (Intr) math   after  money 
math       -0.350                     
after      -0.350  0.000              
money      -0.585  0.000  0.000       
math_first -0.410  0.000  0.000 -0.027
```

```
Linear mixed model fit by REML. t-tests use Satterthwaite's method [
lmerModLmerTest]
Formula: op ~ math + after + money + math_first + (1 | pid)
   Data: dat.asiacan

REML criterion at convergence: 2124.6

Scaled residuals: 
     Min       1Q   Median       3Q      Max 
-2.67305 -0.46014  0.05165  0.59484  2.02007 

Random effects:
 Groups   Name        Variance Std.Dev.
 pid      (Intercept) 213.8    14.62   
 Residual             193.1    13.90   
Number of obs: 252, groups:  pid, 63

Fixed effects:
            Estimate Std. Error      df t value Pr(>|t|)    
(Intercept)    6.947      3.600  76.737   1.930   0.0573 .  
math          -4.040      1.751 187.000  -2.307   0.0221 *  
after         -8.071      1.751 187.000  -4.610 7.45e-06 ***
money          2.085      4.110  60.000   0.507   0.6138    
math_first     7.438      4.106  60.000   1.812   0.0750 .  
---
Signif. codes:  0 '***' 0.001 '**' 0.01 '*' 0.05 '.' 0.1 ' ' 1

Correlation of Fixed Effects:
           (Intr) math   after  money 
math       -0.243                     
after      -0.243  0.000              
money      -0.479  0.000  0.000       
math_first -0.518  0.000  0.000 -0.112
```

```
Linear mixed model fit by REML. t-tests use Satterthwaite's method [
lmerModLmerTest]
Formula: op ~ math + after + money + math_first + (1 | pid)
   Data: dat.hk

REML criterion at convergence: 3742.7

Scaled residuals: 
     Min       1Q   Median       3Q      Max 
-2.70366 -0.62186  0.04252  0.62437  2.17565 

Random effects:
 Groups   Name        Variance Std.Dev.
 pid      (Intercept)  82.27    9.07   
 Residual             268.13   16.37   
Number of obs: 436, groups:  pid, 109

Fixed effects:
            Estimate Std. Error       df t value Pr(>|t|)    
(Intercept)   9.6622     2.1891 184.6544   4.414 1.73e-05 ***
math         -0.7431     1.5684 325.0000  -0.474  0.63596    
after        -4.2844     1.5684 325.0000  -2.732  0.00665 ** 
money        -1.2937     2.3450 106.0000  -0.552  0.58232    
math_first   -1.6286     2.4067 106.0000  -0.677  0.50006    
---
Signif. codes:  0 '***' 0.001 '**' 0.01 '*' 0.05 '.' 0.1 ' ' 1

Correlation of Fixed Effects:
           (Intr) math   after  money 
math       -0.358                     
after      -0.358  0.000              
money      -0.528  0.000  0.000       
math_first -0.444  0.000  0.000  0.039
```

```
Linear mixed model fit by REML. t-tests use Satterthwaite's method [
lmerModLmerTest]
Formula: op ~ math + after + money + math_first + (1 | pid)
   Data: dat.jp

REML criterion at convergence: 2692.2

Scaled residuals: 
    Min      1Q  Median      3Q     Max 
-2.2238 -0.6245  0.0098  0.5417  2.4889 

Random effects:
 Groups   Name        Variance Std.Dev.
 pid      (Intercept) 104.7    10.23   
 Residual             189.3    13.76   
Number of obs: 324, groups:  pid, 81

Fixed effects:
            Estimate Std. Error      df t value Pr(>|t|)
(Intercept)    1.026      2.746 108.049   0.374    0.709
math           1.519      1.529 241.000   0.993    0.322
after         -1.975      1.529 241.000  -1.292    0.198
money          3.720      2.788  78.000   1.334    0.186
math_first     2.496      2.741  78.000   0.910    0.365

Correlation of Fixed Effects:
           (Intr) math   after  money 
math       -0.278                     
after      -0.278  0.000              
money      -0.604  0.000  0.000       
math_first -0.484  0.000  0.000  0.006
```

```
Linear mixed model fit by REML. t-tests use Satterthwaite's method [
lmerModLmerTest]
Formula: true_op ~ math + after + money + math_first + (1 | pid)
   Data: dat.eurocan

REML criterion at convergence: 2473.7

Scaled residuals: 
    Min      1Q  Median      3Q     Max 
-2.5149 -0.6547  0.1139  0.6428  2.3846 

Random effects:
 Groups   Name        Variance Std.Dev.
 pid      (Intercept) 307.8    17.54   
 Residual             571.1    23.90   
Number of obs: 264, groups:  pid, 66

Fixed effects:
            Estimate Std. Error      df t value Pr(>|t|)   
(Intercept)    8.782      5.159  88.753   1.702  0.09222 . 
math          -7.689      2.942 196.000  -2.614  0.00965 **
after         -6.780      2.942 196.000  -2.305  0.02222 * 
money          3.056      5.349  63.000   0.571  0.56977   
math_first     4.047      5.266  63.000   0.768  0.44510   
---
Signif. codes:  0 '***' 0.001 '**' 0.01 '*' 0.05 '.' 0.1 ' ' 1

Correlation of Fixed Effects:
           (Intr) math   after  money 
math       -0.285                     
after      -0.285  0.000              
money      -0.616  0.000  0.000       
math_first -0.432  0.000  0.000 -0.027
```

```
Linear mixed model fit by REML. t-tests use Satterthwaite's method [
lmerModLmerTest]
Formula: true_op ~ math + after + money + math_first + (1 | pid)
   Data: dat.asiacan

REML criterion at convergence: 2301.6

Scaled residuals: 
     Min       1Q   Median       3Q      Max 
-2.07035 -0.69312  0.00239  0.68517  2.31125 

Random effects:
 Groups   Name        Variance Std.Dev.
 pid      (Intercept) 312.2    17.67   
 Residual             428.0    20.69   
Number of obs: 252, groups:  pid, 63

Fixed effects:
            Estimate Std. Error       df t value Pr(>|t|)   
(Intercept)  12.2899     4.6552  83.4514   2.640  0.00989 **
math         -4.3571     2.6063 187.0000  -1.672  0.09625 . 
after        -8.0714     2.6063 187.0000  -3.097  0.00226 **
money         0.1081     5.1977  60.0000   0.021  0.98348   
math_first   -2.6331     5.1925  60.0000  -0.507  0.61395   
---
Signif. codes:  0 '***' 0.001 '**' 0.01 '*' 0.05 '.' 0.1 ' ' 1

Correlation of Fixed Effects:
           (Intr) math   after  money 
math       -0.280                     
after      -0.280  0.000              
money      -0.468  0.000  0.000       
math_first -0.507  0.000  0.000 -0.112
```

```
Linear mixed model fit by REML. t-tests use Satterthwaite's method [
lmerModLmerTest]
Formula: true_op ~ math + after + money + math_first + (1 | pid)
   Data: dat.hk

REML criterion at convergence: 3987.4

Scaled residuals: 
     Min       1Q   Median       3Q      Max 
-2.58273 -0.59381  0.00574  0.61831  2.60070 

Random effects:
 Groups   Name        Variance Std.Dev.
 pid      (Intercept) 242.0    15.55   
 Residual             431.3    20.77   
Number of obs: 436, groups:  pid, 109

Fixed effects:
            Estimate Std. Error       df t value Pr(>|t|)  
(Intercept)   7.0977     3.2131 159.2930   2.209   0.0286 *
math         -0.5596     1.9892 325.0000  -0.281   0.7786  
after        -4.2844     1.9892 325.0000  -2.154   0.0320 *
money         3.7267     3.5892 106.0000   1.038   0.3015  
math_first   -2.0223     3.6836 106.0000  -0.549   0.5842  
---
Signif. codes:  0 '***' 0.001 '**' 0.01 '*' 0.05 '.' 0.1 ' ' 1

Correlation of Fixed Effects:
           (Intr) math   after  money 
math       -0.310                     
after      -0.310  0.000              
money      -0.550  0.000  0.000       
math_first -0.463  0.000  0.000  0.039
```

```
Linear mixed model fit by REML. t-tests use Satterthwaite's method [
lmerModLmerTest]
Formula: true_op ~ math + after + money + math_first + (1 | pid)
   Data: dat.jp

REML criterion at convergence: 3012

Scaled residuals: 
     Min       1Q   Median       3Q      Max 
-3.14827 -0.62093  0.02002  0.67881  2.56013 

Random effects:
 Groups   Name        Variance Std.Dev.
 pid      (Intercept) 280.1    16.74   
 Residual             517.6    22.75   
Number of obs: 324, groups:  pid, 81

Fixed effects:
            Estimate Std. Error      df t value Pr(>|t|)  
(Intercept)   -4.109      4.513 108.513  -0.910   0.3646  
math           1.642      2.528 241.000   0.650   0.5166  
after         -1.975      2.528 241.000  -0.781   0.4353  
money         11.442      4.576  78.000   2.500   0.0145 *
math_first     3.271      4.500  78.000   0.727   0.4695  
---
Signif. codes:  0 '***' 0.001 '**' 0.01 '*' 0.05 '.' 0.1 ' ' 1

Correlation of Fixed Effects:
           (Intr) math   after  money 
math       -0.280                     
after      -0.280  0.000              
money      -0.604  0.000  0.000       
math_first -0.483  0.000  0.000  0.006
```

```
Linear mixed model fit by REML. t-tests use Satterthwaite's method [
lmerModLmerTest]
Formula: zsd ~ math + after + money + math_first + (1 | pid)
   Data: dat.eurocan

REML criterion at convergence: 620.2

Scaled residuals: 
    Min      1Q  Median      3Q     Max 
-2.5896 -0.4214 -0.0630  0.3351  6.4732 

Random effects:
 Groups   Name        Variance Std.Dev.
 pid      (Intercept) 0.383    0.6189  
 Residual             0.402    0.6340  
Number of obs: 264, groups:  pid, 66

Fixed effects:
             Estimate Std. Error        df t value Pr(>|t|)
(Intercept)  -0.25381    0.16423  79.64393  -1.545    0.126
math         -0.05036    0.07804 196.00000  -0.645    0.519
after        -0.01886    0.07804 196.00000  -0.242    0.809
money         0.11744    0.17524  63.00000   0.670    0.505
math_first    0.08787    0.17252  63.00000   0.509    0.612

Correlation of Fixed Effects:
           (Intr) math   after  money 
math       -0.238                     
after      -0.238  0.000              
money      -0.634  0.000  0.000       
math_first -0.444  0.000  0.000 -0.027
```

```
Linear mixed model fit by REML. t-tests use Satterthwaite's method [
lmerModLmerTest]
Formula: zsd ~ math + after + money + math_first + (1 | pid)
   Data: dat.asiacan

REML criterion at convergence: 554.7

Scaled residuals: 
    Min      1Q  Median      3Q     Max 
-3.9754 -0.3933  0.0142  0.3755  4.8596 

Random effects:
 Groups   Name        Variance Std.Dev.
 pid      (Intercept) 0.6778   0.8233  
 Residual             0.2858   0.5346  
Number of obs: 252, groups:  pid, 63

Fixed effects:
              Estimate Std. Error         df t value Pr(>|t|)  
(Intercept)  -0.336362   0.186894  68.517328  -1.800   0.0763 .
math         -0.171946   0.067354 187.000000  -2.553   0.0115 *
after        -0.037818   0.067354 187.000000  -0.561   0.5751  
money         0.220239   0.219740  60.000000   1.002   0.3202  
math_first    0.004021   0.219519  60.000000   0.018   0.9854  
---
Signif. codes:  0 '***' 0.001 '**' 0.01 '*' 0.05 '.' 0.1 ' ' 1

Correlation of Fixed Effects:
           (Intr) math   after  money 
math       -0.180                     
after      -0.180  0.000              
money      -0.493  0.000  0.000       
math_first -0.534  0.000  0.000 -0.112
```

```
Linear mixed model fit by REML. t-tests use Satterthwaite's method [
lmerModLmerTest]
Formula: zsd ~ math + after + money + math_first + (1 | pid)
   Data: dat.hk

REML criterion at convergence: 998.8

Scaled residuals: 
    Min      1Q  Median      3Q     Max 
-3.9907 -0.5158 -0.0459  0.4438  4.1975 

Random effects:
 Groups   Name        Variance Std.Dev.
 pid      (Intercept) 0.6495   0.8059  
 Residual             0.3274   0.5722  
Number of obs: 436, groups:  pid, 109

Fixed effects:
             Estimate Std. Error        df t value Pr(>|t|)    
(Intercept)   0.26756    0.13767 124.73214   1.944  0.05420 .  
math         -0.34924    0.05481 325.00000  -6.372 6.39e-10 ***
after        -0.12139    0.05481 325.00000  -2.215  0.02746 *  
money         0.54088    0.16412 106.00000   3.296  0.00134 ** 
math_first    0.04274    0.16844 106.00000   0.254  0.80018    
---
Signif. codes:  0 '***' 0.001 '**' 0.01 '*' 0.05 '.' 0.1 ' ' 1

Correlation of Fixed Effects:
           (Intr) math   after  money 
math       -0.199                     
after      -0.199  0.000              
money      -0.587  0.000  0.000       
math_first -0.494  0.000  0.000  0.039
```

```
Linear mixed model fit by REML. t-tests use Satterthwaite's method [
lmerModLmerTest]
Formula: zsd ~ math + after + money + math_first + (1 | pid)
   Data: dat.jp

REML criterion at convergence: 783.9

Scaled residuals: 
    Min      1Q  Median      3Q     Max 
-3.1515 -0.3547 -0.0625  0.2572  8.3607 

Random effects:
 Groups   Name        Variance Std.Dev.
 pid      (Intercept) 0.4473   0.6688  
 Residual             0.4242   0.6513  
Number of obs: 324, groups:  pid, 81

Fixed effects:
             Estimate Std. Error        df t value Pr(>|t|)   
(Intercept)  -0.18790    0.16069  96.20176  -1.169  0.24516   
math         -0.23878    0.07237 241.00000  -3.300  0.00111 **
after        -0.04283    0.07237 241.00000  -0.592  0.55454   
money         0.33076    0.16822  78.00000   1.966  0.05283 . 
math_first    0.26063    0.16543  78.00000   1.576  0.11918   
---
Signif. codes:  0 '***' 0.001 '**' 0.01 '*' 0.05 '.' 0.1 ' ' 1

Correlation of Fixed Effects:
           (Intr) math   after  money 
math       -0.225                     
after      -0.225  0.000              
money      -0.623  0.000  0.000       
math_first -0.499  0.000  0.000  0.006
```
