# Supplementary material for: Overconfidence is universal? Elicitation of Genuine Overconfidence (EGO) procedure reveals systematic differences across domain, task knowledge, and incentives in four populations
Source: PLoS One. 2018 Aug 30;13(8):e0202288. doi: 10.1371/journal.pone.0202288 (PMC6116975; doi:10.1371/journal.pone.0202288)
Supplement: S1 File — Contains Analytic Script for Overconfidence.html; Analytic Script for Overconfidence.ipynb; Overconfidence ConsentForm.pdf; Overconfidence_Supplementary.pdf. (ZIP) [file pone.0202288.s001.zip › Overconfidence ConsentForm.pdf]

Department of Psychology
University of British Columbia
Vancouver, B.C.
V6T 1Z4, Canada

CONSENT FORM

Game Show Study

Principal Investigator:
Prof. Joe Henrich, Department of Psychology, UBC
Phone: (604) 822-3007

Co-Investigator:
Michael Muthukrishna, Department of Psychology, UBC
Phone: (604) 828-3342

Introduction and Purpose

The purpose of this study is to test people's performance in a game show.

Study Procedures

If you agree to participate, this study will take approximately 90 minutes to complete. The study involves completing questionnaires and performing a computer task. There are no known risks involved in this study.

Confidentiality

Your identity will be kept strictly confidential. To protect your confidentiality, all documents will be identified only by a randomly generated code number and kept in a locked filing cabinet in a secured laboratory room and/or on a password protected computer. You will not be identified by name in any reports of the completed study. Only the investigators listed above will have access to any of your test results.

Remuneration

The experiment will take no more than 90 minutes and you will receive one 1.5 credit and/or the money you win for your participation.

Contact Information
If you have any questions regarding this study, please contact Michael Muthukrishna (muthukrishna@psych.ubc.ca) via email. Alternatively, you may contact Prof Joe Henrich (604-822-3007).

If you have any concerns about your treatment or rights as a research participant, you may contact the Research Subject Information Line in the UBC Office of Research Services at (604) 822-8598.

Consent
Your participation in this study is entirely voluntary and you may refuse to participate or withdraw from the study at any time without consequences for receiving compensation.

Please feel free to ask the experimenter any additional questions that you have.

Your signature below indicates that you have received a copy of this consent form for your own records.

Your signature below indicates that you consent to participate in this study.

_________________________________ _______________
(Participant signature) (Date)

____
(Participant printed name)
