# Supplementary material for: Overconfidence is universal? Elicitation of Genuine Overconfidence (EGO) procedure reveals systematic differences across domain, task knowledge, and incentives in four populations
Source: PLoS One. 2018 Aug 30;13(8):e0202288. doi: 10.1371/journal.pone.0202288 (PMC6116975; doi:10.1371/journal.pone.0202288)
Supplement: S1 File — Contains Analytic Script for Overconfidence.html; Analytic Script for Overconfidence.ipynb; Overconfidence ConsentForm.pdf; Overconfidence_Supplementary.pdf. (ZIP) [file pone.0202288.s001.zip › Overconfidence_Supplementary.pdf]

Supplementary Materials

for

Overconfidence is Universal? Elicitation of Genuine Overconfidence (EGO) method reveals systematic differences across domain, task knowledge, and incentives in four populations

Michael Muthukrishna[1], Joseph Henrich[2,3], Wataru Toyakawa[4], Takeshi Hamamura[5], Tatsuya Kameda[6], Steven J. Heine[7].

Table of Contents

1. Self Enhancement and Overconfidence 2
2. Incentive graphs 4
3. Performance 5
4. Age and Sex 6
5. Background Questionnaire 9
6. Experiment Protocol 14
   Experimental design 14
   Layout of the Experiment 15
      Participant 15
      Experimenter 15
   Pre-participant Set-up 16
      Paper Materials 16
      Hardware List 16
      Computer Setup 17
   Experiment Procedure 18
      Conditions 0 & 1 18
      Condition 0 & 1 Debriefing 21
      Conditions 2 & 3 23
      Condition 2 & 3 Debriefing 26
   Post-participant Set-down 28
      Data Backup 28

1. Self Enhancement and Overconfidence

Here we report the correlations between self-enhancement and overconfidence variables (Table 3 of main text) for each population separately. We use the average for each participant to avoid inflating power.

Table S1.

Correlation between overconfidence and self-enhancement measures for Euro Canadians

	**Self-esteem**	**False Uniqueness**	**Overconfi-dence**	**True Overconfid-ence**	**Uncertainty in Placement**
**Self-esteem**	1				
**False Uniqueness**	-0.16	1			
**Overconfidence**	0.05	0.15	1		
**True Overconfidence**	-0.09	0.09	0.38**	1	
**Uncertainty in Placement**	0.07	0.09	0.03	-0.20	1

*** p < .001 ** p < .01 * p < .05

Table S2.

Correlation between overconfidence and self-enhancement measures for East Asian Canadians

	**Self-esteem**	**False Uniqueness**	**Overconfi-dence**	**True Overconfid-ence**	**Uncertainty in Placement**
**Self-esteem**	1				
**False Uniqueness**	0.20	1			
**Overconfidence**	0.06	0.35***	1		
**True Overconfidence**	0.00	0.22^{+}	0.39**	1	
**Uncertainty in Placement**	0.07	-0.09	-0.14	-0.05	1

*** p < .001 ** p < .01 * p < .05

Table S3.

Correlation between overconfidence and self-enhancement measures for Hong Kong Chinese

	**Self-esteem**	**False Uniqueness**	**Overconfi-dence**	**True Overconfid-ence**	**Uncertainty in Placement**
**Self-esteem**	1				
**False Uniqueness**	0.31**	1			
**Overconfidence**	0.17^{+}	0.39***	1		
**True Overconfidence**	0.05	0.30**	0.29**	1	
**Uncertainty in Placement**	0.18^{+}	-0.02	-0.31**	0.00	1

*** p < .001 ** p < .01 * p < .05

Table S4.
Correlation between overconfidence and self-enhancement measures for Japanese

	**Self-esteem**	**False Uniqueness**	**Overconfi-dence**	**True Overconfid-ence**	**Uncertainty in Placement**
**Self-esteem**	1				
**False Uniqueness**	0.38***	1			
**Overconfidence**	0.26**	0.41***	1		
**True Overconfidence**	-0.03	0.25*	0.28*	1	
**Uncertainty in Placement**	0.04	-0.21^{+}	-0.09	0.03	1

*** p < .001 ** p < .01 * p < .05

2. Incentive graphs

Here we plot True Overconfidence from Figure 2, 3 and 4 of the main text side by side to more easily compare how incentives affect overconfidence.

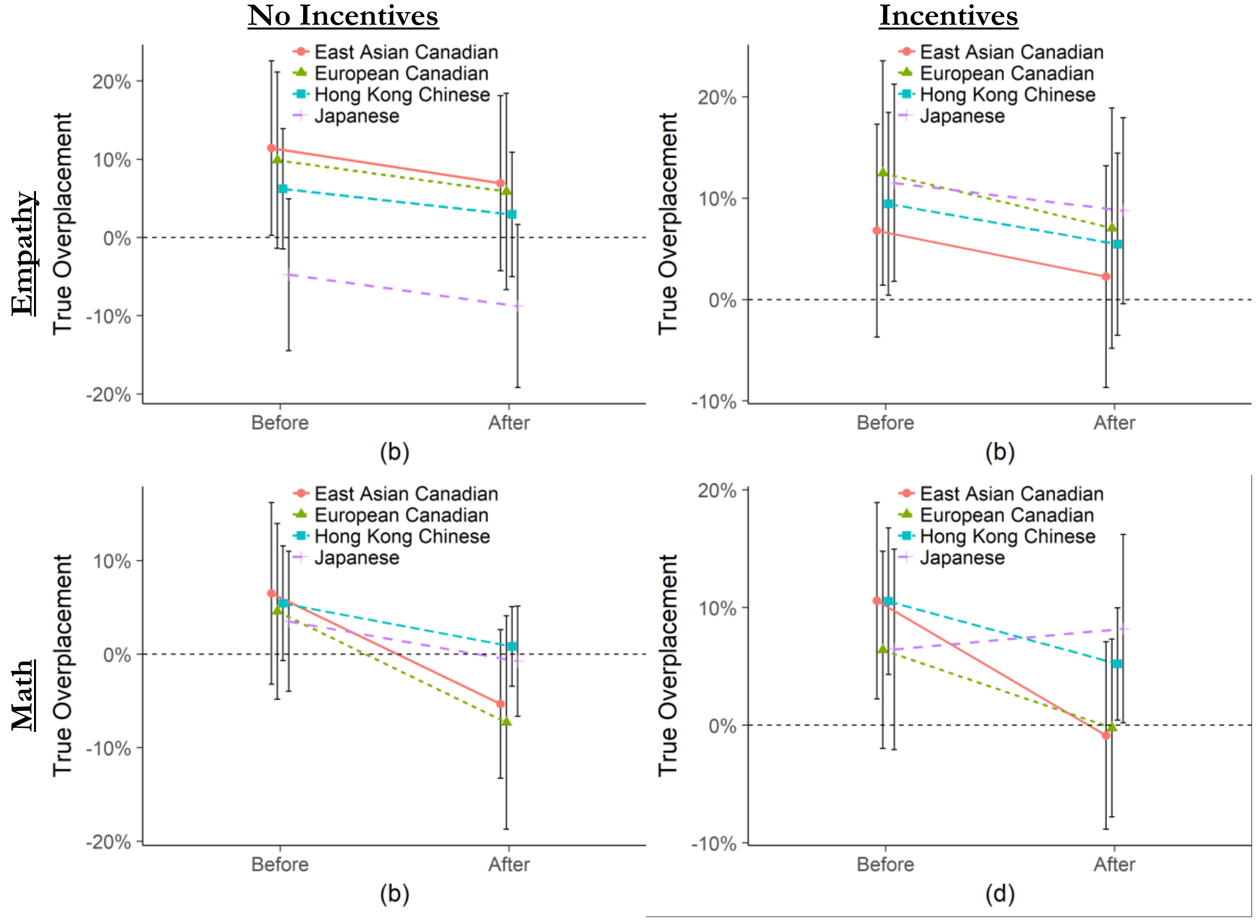

Figure S1. Side by side comparisons of True Overconfidence for empathy and math with and without incentives. Error bars are 95% confidence intervals. Note that the y-axis range is different so as to better visualize the differences between lines.

3. Performance

Here we graph performance in the empathy and math test for each population. The East Asian Canadians and Euro Canadian populations performed better on the empathy test, but the Hong Kong Chinese and Japanese performed better on the math test.

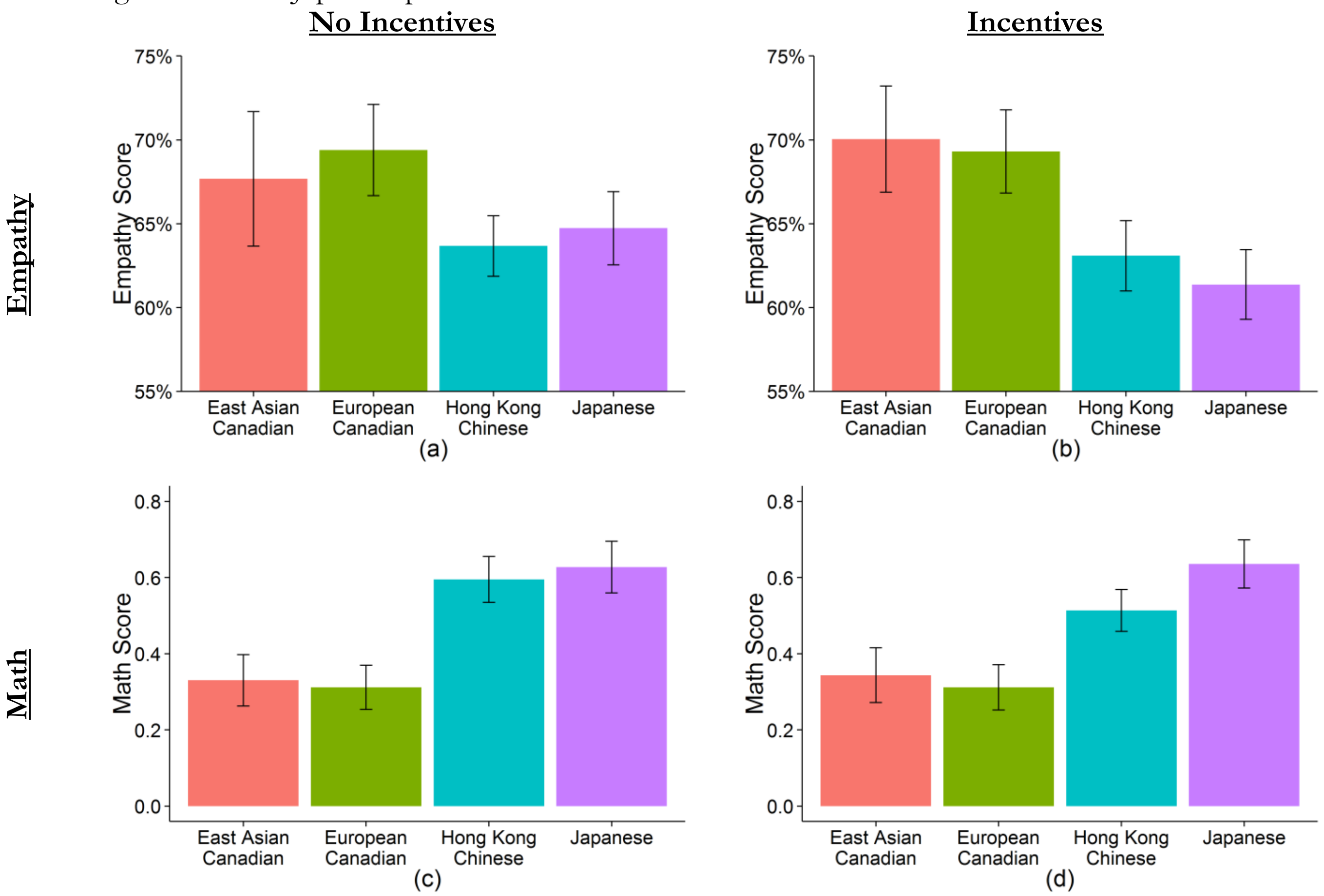

Figure S2. Actual raw performance (i.e. not relative to rest of population) for each population for the Empathy under (a) no incentives and (b) incentives and the Math test under (c) no incentives and (d) incentives. Error bars are 95% confidence intervals.

4. Age and Sex

Here we show the full regressions including age and sex, with (Table S5) and without (Table S6) interactions with population.

Table S5.

Multilevel regression models on the binary variables for task type (Math), updating (After) and incentives (Incentive), with population, age, and sex. We control for common variance from repeated measures using random intercepts for participants.

	Overplacement	True Overplacement	Overprecision	Reward for Accuracy
Intercept	10.04** (3.68, 16.41)	6.04+ (-0.06, 12.13)	-0.10 (-0.33, 0.14)	0.76*** (0.38, 1.14)
Math	-4.48*** (-6.94, -2.02)	-2.27+ (-4.70, 0.16)	-0.23*** (-0.29, -0.16)	0.73*** (0.53, 0.93)
After	-4.94*** (-7.40, -2.48)	-4.94*** (-7.37, -2.51)	-0.06+ (-0.13, 0.01)	0.23* (0.04, 0.43)
Incentive	4.59+ (-0.03, 9.21)	4.58* (0.17, 8.99)	0.36*** (0.19, 0.53)	-0.14 (-0.41, 0.12)
EA Can	-0.35 (-7.53, 6.83)	0.26 (-6.59, 7.12)	-0.11 (-0.39, 0.16)	0.27 (-0.14, 0.69)
HK	-0.43 (-6.81, 5.94)	0.97 (-5.12, 7.05)	0.52*** (0.28, 0.76)	0.03 (-0.34, 0.40)
JP	-0.51 (-7.46, 6.43)	-0.52 (-7.15, 6.11)	0.18 (-0.08, 0.44)	-0.06 (-0.46, 0.35)
Age	1.85 (-0.50, 4.21)	1.85 (-0.40, 4.10)	-0.10* (-0.19, -0.01)	0.04 (-0.10, 0.18)
Male	0.75 (-3.89, 5.39)	-0.60 (-5.03, 3.83)	-0.30*** (-0.47, -0.12)	0.27* (0.004, 0.54)
N	1264 (316 Clusters)	1264 (316 Clusters)	1264 (316 Clusters)	1264 (316 Clusters)
R^2 Fixed	.024	.021	.131	.046
R^2 Total	.395	.372	.644	.205

$^{+}$ $p < .10$ * $p < .05$ ** $p < .01$ *** $p < .001$

Table S6.

Multilevel regression models on the binary variables for task type (Math), updating (After) and incentives (Incentive), with population, age, and sex. This table includes interactions. We control for common variance from repeated measures using random intercepts for participants.

	Overplacement	True Overplacement	Overprecision	Reward for Accuracy
Intercept	15.17** (5.37, 24.98)	10.41* (1.00, 19.82)	-0.26 (-0.62, 0.09)	0.48 (-0.12, 1.08)
Math	-8.75** (-14.13, -3.37)	-7.69** (-12.99, -2.39)	-0.05 (-0.19, 0.09)	1.32*** (0.89, 1.75)
After	-6.78* (-12.16, -1.41)	-6.78* (-12.08, -1.48)	-0.02 (-0.16, 0.13)	0.15 (-0.28, 0.58)
Incentive	0.14 (-10.28, 10.56)	1.50 (-8.45, 11.44)	0.16 (-0.23, 0.55)	-0.35 (-0.95, 0.25)
EA Can	0.64 (-12.82, 14.10)	2.52 (-10.40, 15.44)	0.15 (-0.33, 0.64)	0.33 (-0.50, 1.16)
HK	-9.05 (-21.30, 3.20)	-6.35 (-18.10, 5.41)	0.76*** (0.31, 1.20)	0.61 (-0.15, 1.36)
JP	-8.09 (-23.55, 7.38)	-7.94 (-22.76, 6.88)	0.26 (-0.30, 0.83)	0.17 (-0.77, 1.10)
Age	4.83+ (-0.06, 9.72)	5.19* (0.52, 9.85)	-0.16+ (-0.35, 0.02)	0.35* (0.07, 0.63)
Male	1.17 (-8.97, 11.31)	0.75 (-8.93, 10.43)	0.09 (-0.29, 0.47)	0.48 (-0.10, 1.06)
EA Can: Math	2.96 (-4.73, 10.66)	3.33 (-4.25, 10.92)	-0.12 (-0.33, 0.09)	-0.37 (-0.99, 0.24)
HK:Math	4.62 (-2.21, 11.44)	7.07* (0.34, 13.80)	-0.30** (-0.48, -0.11)	-0.93*** (-1.47, -0.38)
JP: Math	8.40* (1.12, 15.68)	9.37* (2.18, 16.55)	-0.21* (-0.41, -0.01)	-0.79** (-1.37, -0.21)
EA Can:After	-1.29 (-8.98, 6.40)	-1.29 (-8.88, 6.29)	-0.02 (-0.23, 0.19)	0.25 (-0.36, 0.87)
HK:After	2.44 (-4.38, 9.26)	2.44 (-4.29, 9.17)	-0.09 (-0.28, 0.09)	-0.01 (-0.56, 0.53)
JP:After	5.04 (-2.24, 12.32)	5.04 (-2.14, 12.22)	-0.02 (-0.22, 0.18)	0.14 (-0.44, 0.72)
EA Can:Incent	1.78 (-12.99, 16.55)	-0.56 (-14.67, 13.54)	0.13 (-0.42, 0.69)	-0.10 (-0.94, 0.75)
HK:Incent	5.17 (-7.88, 18.22)	2.88 (-9.58, 15.35)	0.37 (-0.12, 0.86)	0.25 (-0.50, 1.00)
JP:Incent	9.57 (-4.48, 23.62)	8.64 (-4.77, 22.06)	0.19 (-0.34, 0.72)	0.35 (-0.46, 1.16)
EA Can:Age	-4.25 (-10.13, 1.64)	-4.65 (-10.27, 0.97)	0.12 (-0.11, 0.34)	-0.45** (-0.78, -0.11)
HK:Age	-5.93 (-13.42, 1.56)	-6.51+ (-13.67, 0.64)	-0.003 (-0.28, 0.28)	-0.16 (-0.60, 0.27)
JP:Age	0.90 (-12.87, 14.67)	0.90 (-12.25, 14.05)	0.08 (-0.43, 0.60)	-0.56 (-1.35, 0.23)
EA Can:Male	-5.66 (-20.29, 8.97)	-5.85 (-19.83, 8.12)	-0.63* (-1.18, -0.08)	0.18 (-0.66, 1.02)
HK:Male	6.31 (-6.68, 19.29)	3.81 (-8.59, 16.21)	-0.52* (-1.00, -0.03)	-0.49 (-1.24, 0.26)
JP:Male	-4.48 (-18.50, 9.54)	-4.53 (-17.91, 8.85)	-0.23 (-0.76, 0.30)	-0.31 (-1.12, 0.50)
N	1264 (316 Clusters)	1264 (316 Clusters)	1264 (316 Clusters)	1264 (316 Clusters)
R^2 Fixed	.042	.041	.155	.067
R^2 Total	.407	.386	.653	.222

$^{+}$ $p < .10$ * $p < .05$ ** $p < .01$ *** $p < .001$

Here we report the mean and standard deviation reward for accuracy for each gender and each population. Means can be treated as CAD, where we assume 1 CAD = 10 HKD = 100 JPY. Male participants universally had a higher reward for accuracy, but also universally had more variance in this reward.

Table S7.
Mean and standard deviation for each gender and population.

	**Female**	**Male**	
**Euro Canadian**	$1.11 (1.98)	$1.48 (2.34)	$1.29 (2.17)
**East Asian Canadian**	$1.27 (2.04)	$1.91 (2.51)	$1.57 (2.29)
**Hong Kong Chinese**	$1.29 (1.55)	$1.36 (2.11)	$1.32 (1.83)
**Japanese**	$1.12 (1.65)	$1.32 (1.84)	$1.25 (1.78)
	$1.21 (1.78)	$1.47 (2.16)	$1.35 (1.99)

5. Background Questionnaire

*** 1. Age**

*** 2. Gender**

- Male
- Female

Other (please specify)

*** 3. What degree are you studying for? (e.g. B Arts, B Sc)**

*** 4. What is your major? (e.g. Chemistry, Computer Science)**

*** 5. Have you lived your entire life in Canada?**

- Yes
- No

IF NO, how long have you lived in Canada?
Where else have you lived? (Please list)

6. Where else have you lived? (Please list)

*** 7. What suburb do/did you live in for most of your time in Canada?**

*** 8. Please specify the ethnic (cultural) group you primarily identify with (e.g. Punjabi, Cantonese Chinese, Mandarin Chinese, Japanese, European, etc.)**

*** 9. What is the native language of your ethnic group?**

**** 10. What generation Canadian are you?***
Grandparents or earlier born in Canada = 4th generation
Parents born in Canada = 3rd generation
You were born in Canada = 2nd generation
You were born elsewhere = 1st generation

**** 11. How well do you speak the native language of your ethnic group?***

Not At All Very Well

Pick the pair of circles that you feel best represents your own level of identification
with your ethnic group (S=Self, G=Group):

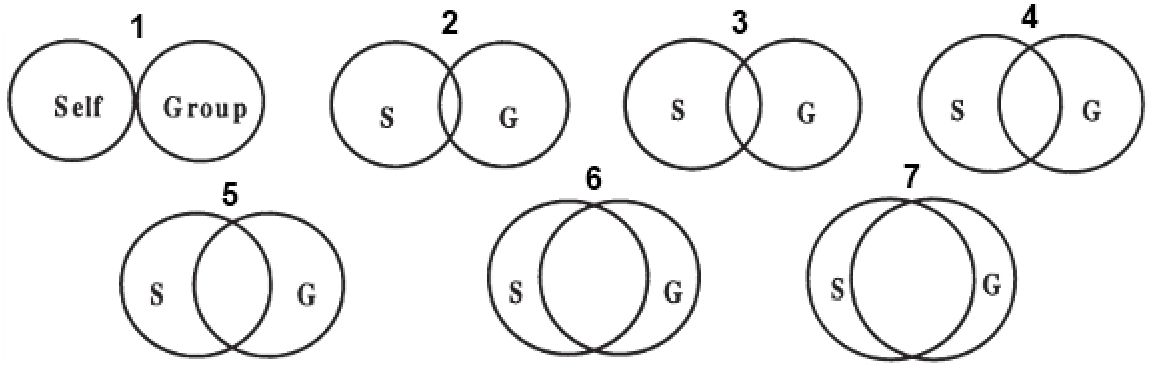

**** 12. Please pick from below:***

- 1
- 2
- 3
- 4
- 5
- 6
- 7

Pick the pair of circles that you feel best represents your own level of identification with other Canadians (S=Self, G=Group):

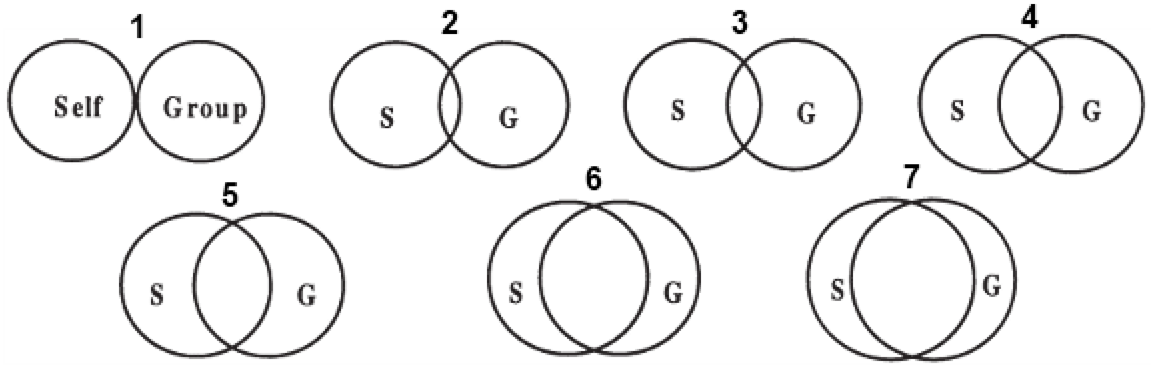

**** 13. Please pick from below:***

- ○ 1
- ○ 2
- ○ 3
- ○ 4
- ○ 5
- ○ 6
- ○ 7

Your religious background:

**** 14. Your religious background:***

- ☐ Buddhism
- ☐ Catholicism
- ☐ Christianity
- ☐ Hinduism
- ☐ Islam
- ☐ Protestantism
- ☐ Sikhism
- ☐ None
- ☐ Other:

Other (please specify) ____________

**** 15. How important is religion in your daily life?***

Not At All	Very Important

**** 16. Think of the last three social events you were at. How many of these social events had a majority of your own ethnic group?***

- 0
- 1
- 2
- 3

**** 17. What are the ethnicities of your friends?***

All my ethnicity	All different ethnicity to me

**** 18. What language do you speak at home?***

**** 19. What is the ethnicity of your partner (e.g. husband, girlfriend)?***

- No partner
- My ethnicity
- Different ethnicity

**** 20. Please indicate the extent to which each statement accurately describes you by ticking the appropriate box. For each question, replace "Asian" with your own ethnicity:***

	Completely Disagree	Completely Agree
I am simply an Asian who lives in North America.		
I keep Asian and Canadian cultures separate.		
I feel part of a combined culture.		
I am conflicted between the Canadian and Asian way of doing things.		
I feel like someone moving between two cultures.		
I feel caught between Asian and Canadian cultures.		

	Completely Disagree	Completely Agree
I don't feel trapped between Asian and Canadian cultures.		

*** 21. How important is...**

	Extremely unimportant	Very unimportant	Somewhat unimportant	Neither unimportant nor important	Somewhat important	Very important	Extremely important
Math to who you are?							
Empathy to who you are?							
Math to your identity?							
Empathy to your identity?							
Math to success in life?							
Empathy to success in life?							
Math to being a good person?							
Empathy to being a good person?							

*** 22. Who do you think is better at the following?**

	East Asians are very much better	East Asians are much better	East Asians are slightly better	Both are equal	Caucasians are slightly better	Caucasians are much better	Caucasians are very much better
Math							
Empathy							

*** 23. How well do you think the tests you took measured what they intended to measure?**

	Not at all	Very little	Somewhat	To a great extent	Perfectly
Math					
Empathy					

* 24. How concrete or abstract do you think the following are?

	Completely abstract	Neither abstract nor concrete	Completely concrete
Math			
Empathy			

25. Have you ever taken a similar survey?

- Yes
- No

26. Can you describe what this survey was about?

6. Experiment Protocol

Experimental design

The experimental design is as follows:

Abstract vs Concrete task is tested **within** subjects (randomly assigned) – A vs C.

Money vs Tokens is tested **between** subjects (randomly assigned) - M vs. T

Ethnicity (East Asian vs. White) is tested between subjects E vs. W (cannot be randomly assigned).

Layout of the Experiment

Participant

- Participants sit in a chair in front of the desktop or laptop.

Experimenter

- Trains participant in betting, then sets up experiment to start on instructions page. Experiment leaves participant in room. Participant will call experimenter at various times during the experiment.

Pre-participant Set-up

Paper Materials

1. Informed Consent forms (5-10)
2. Debriefing Sheets (5-10)
3. Payment Sheet
4. Eyes task definition sheet
5. Paper and pen for math problems – remove front page so not exam booklet
6. Lottery to assign which condition they will be paid for
7. Raffle Entries (10)
8. Participant ID and random assignment sheet
9. Betting grid, coins, and tokens
10. Timers (2 – one for participant, 1 for experimenter)

Hardware List

If laptop

- 1 laptop
- 1 Power cord
- 1 mouse

If desktop

- PC with accessories

Computer Setup

1. Ensure that computer has an internet connection by opening a web browser and going to www.google.com.
2. Open Experiment Software
3. When you reach point in protocol, complete all sections of first window and click “Start Experiment”. This is done after you have seen the participant.

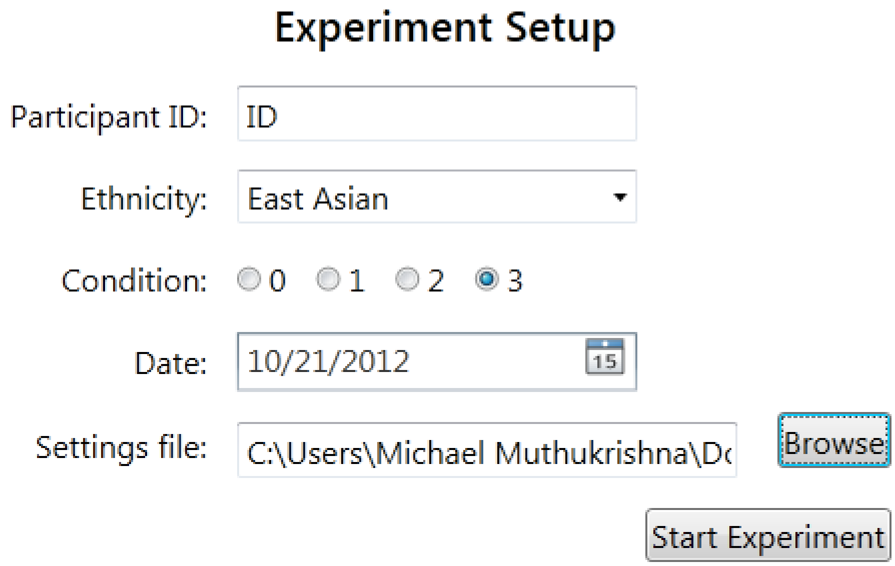

   a) Participant ID: Use next value in randomization sheet
   b) Ethnicity: Fill out what ethnicity the participant looks like – White or East Asian. If Other, please enter into Combo Box
   c) Condition: Select condition from Randomization Sheet
   d) Date: Select Today
   e) Settings file: Select file that matches the condition
4. Click “Start Experiment” and ensure that the instructions appear on the screen.

Experiment Procedure

Conditions 0 & 1

Briefing

Introduce yourself.

I'm going to be reading most of what I have to say today. This is to ensure that all participants are given the same information in the same way. However, please feel free to ask questions as we go along.

You are invited to participate in a study where you will take game show type quizzes. You will predict your performance for each quiz. You can win money based on how well you do in the quiz. For every question you get correct, you will get an entry into a competition to win $100. This is a real competition with real money.

More details are provided in this informed consent form. Please read the form to understand what will be expected of you during this experiment.

Give participant the informed consent form.

When you're ready please read and sign the consent form. Let me know if you have any questions.

Wait for questions

If there are no further questions and you are happy to continue, we may now begin the experiment. Can I first ask you to please turn off your mobile phone or other electronic devices.

Wait.

We will start by going through what you will be doing. You will be taking various tests during this study. For every question you get correct, you will get an entry into a draw for $100. We are also interested in how you think you compare to other participants in this study. At various stages during this study, we will give you 10 tokens which you will place in deciles to indicate how you think you will perform compared to everyone else who has taken this quiz. Remember, you are not predicting your score, but how you compare to all other participants in this experiment.

Make sure board is ready.

I will now go through some of the concepts that are important to understand in this experiment. First, throughout this study we will ask you how you think your performance on the quizzes compares to other participants. These other participants are other psychology students who have participated in this study. You are only comparing yourself to these other participants. Does this make sense?

Wait for affirmative answer and re-explain in other words if they say "no".

To tell us how you think you will perform relative to other participants, we will ask you to indicate what percentile and decile you think you will fall into. A percentile is the percentage of other participants that you got a better score than. For example, if you were in the 64th percentile, you would be better than 64% of other participants, the 10th percentile

means you are better than 10% of other participants and the 99th percentile means that you are better than 99% of other participants. Does this make sense?

Wait for affirmative answer and re-explain in other words if they say "no".

> *A decile is a space of 10 percentiles. So for example, if you were in the 80-89th percentile, you would be better than somewhere between 80 and 89% of other participants. If you were in the 10-19th decile, you would be better than 10-19% of other participants. Does this make sense?*

Wait for affirmative answer and re-explain in other words if they say "no".

> *To indicate what decile you think your score will put you in compared to other participants, you will place 10 tokens onto a grid like this. You would put more tokens into the decile you think your score in a quiz is more likely to be in. You may use as few or as many deciles as you wish, but you must allocate all tokens. Does this make sense?*

Wait for affirmative answer and re-explain in other words if they say "no".

> *You can win money by doing your best in each quiz. For every question you get correct, you will get an additional entry into a draw for $100. Make sense?*

Check that they understand these concepts, by asking:

> *To check that you understand all instructions, I will now ask you a few questions:*
> 1. *Who are competing with?*
> 2. *What is a percentile?*
> 3. *What is a decile?*
> 4. *How can you win money?*

When confident that they understand everything:

Ok, we can now begin the experiment. Give me a second while I set it up.

Experiment

Set up experiment as per Section 0. Make sure the participant only sees the instructions screen. When this screen is up, lead participant into the room and let them go through experiment by themselves.

> *The experimental software will ask you to call the experimenter at various stages. Please let me know when this happens. I will be just outside. You can click "Next" when you're ready.*

Empathy Test

The participant will call you into the room before they begin the empathy test.

> *The empathy test will involve you reading thoughts and feelings in people's eyes. This is a list of definitions in case you are unfamiliar with any words. There is no time limit, but please try to complete the task quickly. You may click "Next" as soon as I leave the room. It may take a minute to start the quiz. Please be patient.*

Give participants the list of definitions.

Math Test

The participant will call you into the room before they begin the math test. You will give them a timer.

> *The math test involves you answering 30 problem solving questions, one at a time. You must answer each question before moving onto the next one and you cannot skip a question. The survey says 31, but it's including your participant ID as a question. There are only 30 questions. You have 20 minutes to complete this task, which means you may not finish all questions. You should attempt to answer as many questions as possible in the time allotted, but you should avoid random guessing. This timer will tell you how much time you have left. You may use this pen and paper if you need it. You may click "Next" as soon as I leave the room. I will stop you when 20 minutes is up.*

Give participant sheet of paper, pen, set timer and start.

Condition 0 & 1 Debriefing

Ensure next participant is not around during debriefing. You are trying to discover if (1) the participant guessed that we were interested in ethnic differences in confidence and (2) were using a weird strategy to allocate tokens. Please try to follow the script, but you may skip, reword, and add things to achieve this purpose.

Thank you, the experiment is now over and I am going to explain a little more about what our lab is investigating, but first I have a few questions.

1. *First, what did you think of the task? Did you have any concerns with what you had to do? Did you understand all the instructions?*

Write responses.

2. *When you were using the decile grid, how were you allocating the tokens? What strategy were you using?*

Write responses.

3. *What sorts of things do you think we are looking for in this particular study?*

Write responses.

4. *Sometimes when people participate in psychology studies they think that maybe there was something else going on behind the scenes. Did anything like that come to mind while you were in this study?*

Write responses.

Now let me tell you more about what we are studying and what we hope to find. This is a new study that we have just begun so I'm really hoping you can help us troubleshoot some of our procedures to make sure the study runs as smoothly as possible.

In our lab, we study how confidence levels differ between cultures. In particular we are interested in how accurate people's confidence in their own abilities are.

5. *At any time during today's session, did you think we might be interested in studying something about confidence? What made you think that? What did you think we might be trying to find?*

Write responses.

6. *At any time during today's session, did you think we might be interested in studying something about ethnic differences in confidence? What made you think that? What did you think we might be trying to find?*

Write responses.

7. *If you remember, we asked you how many tokens/much money you would like to bet on your performance on a general test. This was to test how much confidence you had in your own abilities. Did you know that this was what we were testing?*

Write responses.

The last thing that I want to discuss with you is your knowledge about the true purpose of this study. We will be running this study for the duration of the year with other students that you may know or that may be in your class. As you can imagine, it would be very difficult for us to collect accurate information if people knew about the true purpose of this study beforehand. Consequently, I have to ask you not to discuss this experiment with other students. We're asking that you agree not to discuss this experiment with anyone. So I just need a verbal agreement from you, can you do that? **Make sure to get verbal agreement!**

Finally, do you have any suggestions or remaining questions about this experiment?

Again, thank you so much for participating in this research. Without your help we would be unable to test our hypotheses and gather the necessary data. If you have any questions about the study please contact Michael Muthukrishna or Dr. Joseph Henrich whose information is provided on the consent form.

Please pick a number from this box to determine which condition you will be paid for.

Have participant randomly select a number.

Please complete this form so we can pay you if you win the $100.

1. Ask participant to fill out details for competition – write participant number
2. Hand participant debriefing sheet and have them fill out HSP form.

Before participant leaves you should have 2 things: competition entry and completed HSP form.

Conditions 2 & 3

Briefing

Introduce yourself.

I'm going to be reading most of what I have to say today. This is to ensure that all participants are given the same information in the same way. However, please feel free to ask questions as we go along.

You are invited to participate in a study where you will take game show type quizzes. You will predict your performance for each quiz. You can win money based on how well you do in the quiz. For every question you get correct, you will get an entry into a competition to win $100. This is a real competition with real money. You can also win money by accurately predicting how well you will perform in the quiz relative to other participants in this experiment.

More details are provided in this informed consent form. Please read the form to understand what will be expected of you during this experiment.

Give participant the informed consent form.

When you're ready please read and sign the consent form. Let me know if you have any questions.

Wait for questions

If there are no further questions and you are happy to continue, we may now begin the experiment. Can I first ask you to please turn off your mobile phone or other electronic devices.

Wait.

We will start by going through what you will be doing. You will be doing various tests during this study. For every question you get correct, you will get an entry into a draw for $100. We are also interested in how you think you compare to other participants in this study. At various stages during this study, we will give you $10 which you will place into deciles to indicate how you think you will perform compared to everyone else who has taken this quiz. Remember, you are not predicting your score, but how you compare to other participants in this experiment. At the end of the experiment, you will randomly select one of the times you predicted your performance. Any money you place in the decile you actually perform in is yours to keep.

Make sure board is ready.

I will now go through some of the concepts that are important to understand in this experiment. First, throughout this study we will ask you how you think your performance on the quizzes compares to other participants. These other participants are other psychology students who have participated in this study. You are only comparing yourself to these other participants. Does this make sense?

Wait for affirmative answer and re-explain in other words if they say "no".

To tell us how you think you will perform relative to other participants, we will ask you to indicate what percentile and decile you think you will fall into. A percentile is the percentage of other participants that you got a better score than.

For example, if you were in the 64th percentile, you would be better than 64% of other participants, the 10th percentile means you are better than 10% of other participants and the 99th percentile means that you are better than 99% of other participants. Does this make sense?

Wait for affirmative answer and re-explain in other words if they say "no".

> *A decile is a space of 10 percentiles. So for example, if you were in the 80-89th percentile, you would be better than somewhere between 80 and 89% of other participants. If you were in the 10-19th decile, you would be better than 10-19% of other participants. Does this make sense?*

Wait for affirmative answer and re-explain in other words if they say "no".

> *To indicate what decile you think your score will put you in compared to other participants, you will place $10 onto a grid like this. You would put more loonies into the decile you think you your score in a quiz is more likely to be in. You may use as few or as many deciles as you wish, but you must allocate all $10. Does this make sense? For one randomly chosen round, any money you placed into the decile that your score actually appears in, is yours to keep. So you can win up to $10 today.*

Wait for affirmative answer and re-explain in other words if they say "no".

> *You can win money by doing your best in each quiz. For every question you get correct, you will get an additional entry into a draw for $100. Make sense?*

Check that they understand these concepts, by asking:

> *To check that you understand all instructions, I will now ask you a few questions:*
> 1. *Who are competing with?*
> 2. *What is a percentile?*
> 3. *What is a decile?*
> 4. *How can you win money?*

When confident that they understand everything:

Ok, we can now begin the experiment. Give me a second while I set it up.

Experiment

Set up experiment as per Section 0. Make sure the participant only sees the instructions screen. When this screen is up, lead participant into the room and let them go through experiment by themselves.

> *The experimental software will ask you to call the experimenter at various stages. Please let me know when this happens. I will be just outside. Please click "Next" when you are ready.*

Empathy Test

The participant will call you into the room before they begin the empathy test. Give them the list of definitions.

> *The empathy test will involve you reading thoughts and feelings in people's eyes. This is a list of definitions in case you are unfamiliar with any words. There is no time limit, but please try to complete the task quickly. You may click "Next" as soon as I leave the room.*

Math Test

The participant will call you into the room before they begin the math test. You will give them a timer.

> *The math test involves you answering 30 problem solving questions, one at a time. You must answer each question before moving onto the next one and you cannot skip a question. The survey says 31, but it's including your participant ID as a question. There are only 30 questions. You have 20 minutes to complete this task, which means you may not finish all questions. You should attempt to answer as many questions as possible in the time allotted, but you should avoid random guessing. This timer will tell you how much time you have left. You may use this pen and paper if you need it. You may click "Next" as soon as I leave the room. I will stop you when 20 minutes is up.*

Give participant sheet of paper and pen.

Condition 2 & 3 Debriefing

Ensure next participant is not around during debriefing. You are trying to discover if (1) the participant guessed that we were interested in ethnic differences in confidence and (2) were using a weird strategy to allocate money. Please try to follow the script, but you may skip, reword, and add things to achieve this purpose.

Thank you, the experiment is now over and I am going to explain a little more about what our lab is investigating, but first I have a few questions.

1. *First, what did you think of the task? Did you have any concerns with what you had to do? Did you understand all the instructions?*

Write responses.

2. *When you were using the decile grid, how were you allocating the money? What strategy were you using?*

Write responses.

3. *What sorts of things do you think we are looking for in this particular study?*

Write responses.

4. *Sometimes when people participate in psychology studies they think that maybe there was something else going on behind the scenes. Did anything like that come to mind while you were in this study?*

Write responses.

Now let me tell you more about what we are studying and what we hope to find. This is a new study that we have just begun so I'm really hoping you can help us troubleshoot some of our procedures to make sure the study runs as smoothly as possible.

In our lab, we study how confidence levels differ between cultures. In particular we are interested in how accurate people's confidence in their own abilities are.

5. *At any time during today's session, did you think we might be interested in studying something about confidence? What made you think that? What did you think we might be trying to find?*

Write responses.

6. *At any time during today's session, did you think we might be interested in studying something about ethnic differences in confidence? What made you think that? What did you think we might be trying to find?*

Write responses.

7. *If you remember, we asked you how many tokens/much money you would like to bet on your performance on a general test. This was to test how much confidence you had in your own abilities. Did you know that this was what we were testing?*

Write responses.

The last thing that I want to discuss with you is your knowledge about the true purpose of this study. We will be running this study for the duration of the year with other students that you may know or that may be in your class. As you can imagine, it would be very difficult for us to collect accurate information if people knew about the true purpose of this study beforehand. Consequently, I have to ask you not to discuss this experiment with other students. We're asking that you agree not to discuss this experiment with anyone. So I just need a verbal agreement from you, can you do that? ***Make sure to get verbal agreement!***

Finally, do you have any suggestions or remaining questions about this experiment?

Again, thank you so much for participating in this research. Without your help we would be unable to test our hypotheses and gather the necessary data. If you have any questions about the study please contact Michael Muthukrishna or Dr. Joseph Henrich whose information is provided on the consent form.

Please pick a number from this box to determine which condition you will be paid for.

Have participant randomly select a number.

Please complete this form so we can pay you if you win the $100. I will check what decile you were in and pay you accordingly.

1. Ask participant to fill out details for competition – write participant number
2. Use software to check how much participant needs to be paid.
3. Pay participant, fill in value, and have them sign confirmation of payment.
4. Hand participant debriefing sheet and have them fill out HSP form.

Before participant leaves you should have 3 things: competition entry, completed HSP form, and signed payment confirmation.

Post-participant Set-down

Data Backup

- Record participant debriefing information
- Copy log files and email to Michael
- Shut down Laptops/Desktops
- Take all study materials – sheets and money – back to the lab
