# Supplementary material for: Overconfidence is universal? Elicitation of Genuine Overconfidence (EGO) procedure reveals systematic differences across domain, task knowledge, and incentives in four populations
Source: PLoS One. 2018 Aug 30;13(8):e0202288. doi: 10.1371/journal.pone.0202288 (PMC6116975; doi:10.1371/journal.pone.0202288)
Supplement: S1 Table — (PDF) [file pone.0202288.s002.pdf]

Table S1.
Correlation between overconfidence and self-enhancement measures for Euro Canadians

	**Self-esteem**	**False Uniqueness**	**Overconfidence**	**True Overconfidence**	**Uncertainty in Placement**
**Self-esteem**	1				
**False Uniqueness**	-0.16	1			
**Overconfidence**	0.05	0.15	1		
**True Overconfidence**	-0.09	0.09	0.38**	1	
**Uncertainty in Placement**	0.07	0.09	0.03	-0.20	1

*** p < .001 ** p < .01 * p < .05
