# Supplementary material for: Overconfidence is universal? Elicitation of Genuine Overconfidence (EGO) procedure reveals systematic differences across domain, task knowledge, and incentives in four populations
Source: PLoS One. 2018 Aug 30;13(8):e0202288. doi: 10.1371/journal.pone.0202288 (PMC6116975; doi:10.1371/journal.pone.0202288)
Supplement: S2 Table — (PDF) [file pone.0202288.s003.pdf]

Table S2.
Correlation between overconfidence and self-enhancement measures for East Asian Canadians

	**Self-esteem**	**False Uniqueness**	**Overconfidence**	**True Overconfidence**	**Uncertainty in Placement**
**Self-esteem**	1				
**False Uniqueness**	0.20	1			
**Overconfidence**	0.06	0.35***	1		
**True Overconfidence**	0.00	0.22^{+}	0.39**	1	
**Uncertainty in Placement**	0.07	-0.09	-0.14	-0.05	1

*** $p < .001$ ** $p < .01$ * $p < .05$
