# Supplementary material for: Overconfidence is universal? Elicitation of Genuine Overconfidence (EGO) procedure reveals systematic differences across domain, task knowledge, and incentives in four populations
Source: PLoS One. 2018 Aug 30;13(8):e0202288. doi: 10.1371/journal.pone.0202288 (PMC6116975; doi:10.1371/journal.pone.0202288)
Supplement: S3 Table — (PDF) [file pone.0202288.s004.pdf]

Table S3.

Correlation between overconfidence and self-enhancement measures for Hong Kong Chinese

	**Self-esteem**	**False Uniqueness**	**Overconfidence**	**True Overconfidence**	**Uncertainty in Placement**
**Self-esteem**	1				
**False Uniqueness**	0.31**	1			
**Overconfidence**	0.17^{+}	0.39***	1		
**True Overconfidence**	0.05	0.30**	0.29**	1	
**Uncertainty in Placement**	0.18^{+}	-0.02	-0.31**	0.00	1

*** p < .001 ** p < .01 * p < .05
