# Supplementary material for: Overconfidence is universal? Elicitation of Genuine Overconfidence (EGO) procedure reveals systematic differences across domain, task knowledge, and incentives in four populations
Source: PLoS One. 2018 Aug 30;13(8):e0202288. doi: 10.1371/journal.pone.0202288 (PMC6116975; doi:10.1371/journal.pone.0202288)
Supplement: S4 Table — (PDF) [file pone.0202288.s005.pdf]

Table S4.

Correlation between overconfidence and self-enhancement measures for Japanese

	**Self-esteem**	**False Uniqueness**	**Overconfidence**	**True Overconfidence**	**Uncertainty in Placement**
**Self-esteem**	1				
**False Uniqueness**	0.38***	1			
**Overconfidence**	0.26**	0.41***	1		
**True Overconfidence**	-0.03	0.25*	0.28*	1	
**Uncertainty in Placement**	0.04	-0.21^{+}	-0.09	0.03	1

*** $p < .001$ ** $p < .01$ * $p < .05$
