# Supplementary material for: Overconfidence is universal? Elicitation of Genuine Overconfidence (EGO) procedure reveals systematic differences across domain, task knowledge, and incentives in four populations
Source: PLoS One. 2018 Aug 30;13(8):e0202288. doi: 10.1371/journal.pone.0202288 (PMC6116975; doi:10.1371/journal.pone.0202288)
Supplement: S5 Table — Multilevel regression models on the binary variables for task type (Math), updating (After) and incentives (Incentive), with population, age, and sex. We control for common variance from repeated measures using random intercepts for participants. (PDF) [file pone.0202288.s006.pdf]

Table S5.

Multilevel regression models on the binary variables for task type (Math), updating (After) and incentives (Incentive), with population, age, and sex. We control for common variance from repeated measures using random intercepts for participants.

	Overplacement	True Overplacement	Overprecision	Reward for Accuracy
Intercept	10.04** (3.68, 16.41)	6.04+ (-0.06, 12.13)	-0.10 (-0.33, 0.14)	0.76*** (0.38, 1.14)
Math	-4.48*** (-6.94, -2.02)	-2.27+ (-4.70, 0.16)	-0.23*** (-0.29, -0.16)	0.73*** (0.53, 0.93)
After	-4.94*** (-7.40, -2.48)	-4.94*** (-7.37, -2.51)	-0.06+ (-0.13, 0.01)	0.23* (0.04, 0.43)
Incentive	4.59+ (-0.03, 9.21)	4.58* (0.17, 8.99)	0.36*** (0.19, 0.53)	-0.14 (-0.41, 0.12)
EA Can	-0.35 (-7.53, 6.83)	0.26 (-6.59, 7.12)	-0.11 (-0.39, 0.16)	0.27 (-0.14, 0.69)
HK	-0.43 (-6.81, 5.94)	0.97 (-5.12, 7.05)	0.52*** (0.28, 0.76)	0.03 (-0.34, 0.40)
JP	-0.51 (-7.46, 6.43)	-0.52 (-7.15, 6.11)	0.18 (-0.08, 0.44)	-0.06 (-0.46, 0.35)
Age	1.85 (-0.50, 4.21)	1.85 (-0.40, 4.10)	-0.10* (-0.19, -0.01)	0.04 (-0.10, 0.18)
Male	0.75 (-3.89, 5.39)	-0.60 (-5.03, 3.83)	-0.30*** (-0.47, -0.12)	0.27* (0.004, 0.54)
N	1264 (316 Clusters)	1264 (316 Clusters)	1264 (316 Clusters)	1264 (316 Clusters)
R^2 Fixed	.024	.021	.131	.046
R^2 Total	.395	.372	.644	.205

$^{+}$ $p < .10$ * $p < .05$ ** $p < .01$ *** $p < .001$
