# Supplementary figures and images for: Overconfidence is universal? Elicitation of Genuine Overconfidence (EGO) procedure reveals systematic differences across domain, task knowledge, and incentives in four populations
Source: PLoS One. 2018 Aug 30;13(8):e0202288. doi: 10.1371/journal.pone.0202288 (PMC6116975; doi:10.1371/journal.pone.0202288)

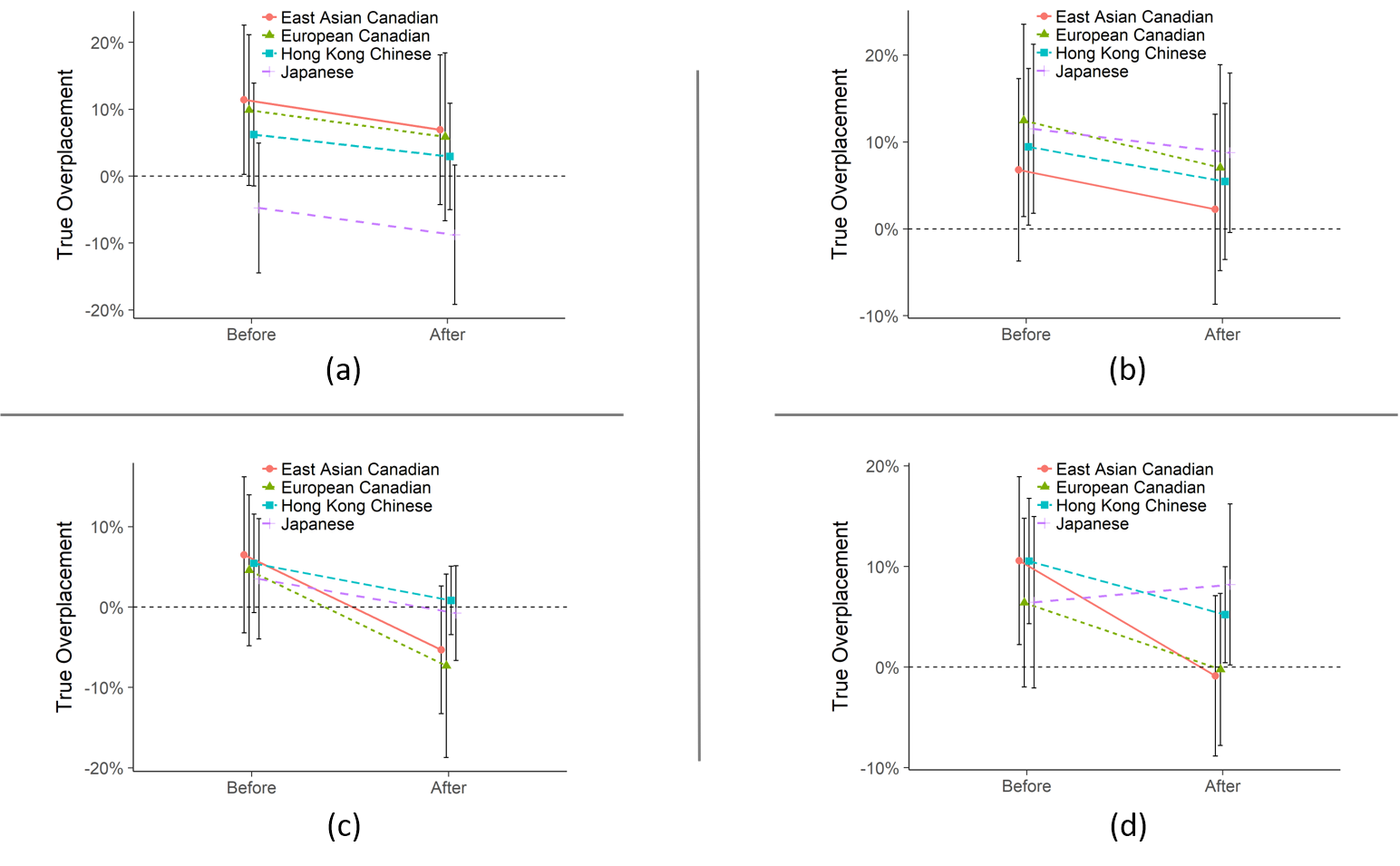

Supplement: S1 Fig — Error bars are 95% confidence intervals. Note that the y-axis range is different so as to better visualize the differences between lines. (TIF) [file pone.0202288.s008.tif]

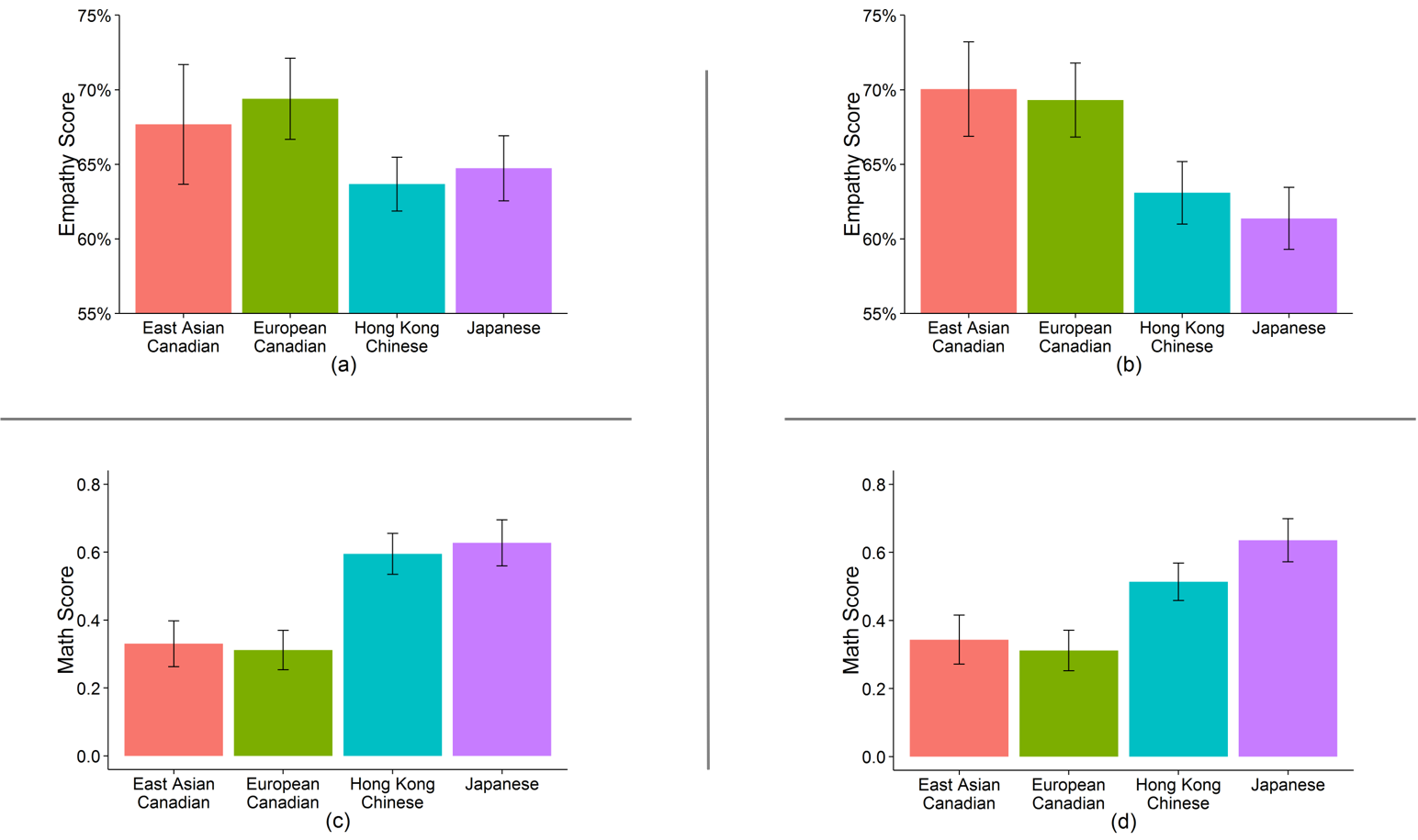

Supplement: S2 Fig — For the Empathy under (a) no incentives and (b) incentives and the Math test under (c) no incentives and (d) incentives. Error bars are 95% confidence intervals. (TIF) [file pone.0202288.s009.tif]
